# Lower Risk of Heart Failure and Death in Patients Initiated on Sodium-Glucose Cotransporter-2 Inhibitors Versus Other Glucose-Lowering Drugs: The CVD-REAL Study (Comparative Effectiveness of Cardiovascular Outcomes in New Users of Sodium-Glucose Cotransporter-2 Inhibitors)

**DOI:** 10.1161/CIRCULATIONAHA.117.029190

**Published:** 2017-05-18

**Authors:** Mikhail Kosiborod, Matthew A. Cavender, Alex Z. Fu, John P. Wilding, Kamlesh Khunti, Reinhard W. Holl, Anna Norhammar, Kåre I. Birkeland, Marit Eika Jørgensen, Marcus Thuresson, Niki Arya, Johan Bodegård, Niklas Hammar, Peter Fenici

**Affiliations:** From Saint Luke’s Mid America Heart Institute and University of Missouri–Kansas City (M.K.); University of North Carolina, Chapel Hill (M.A.C.); Georgetown University Medical Center, Washington, DC (A.Z.F.); University of Liverpool, United Kingdom (J.P.W.); University of Leicester, United Kingdom (K.K.); University of Ulm, Germany (R.W.H.); Karolinska Institutet, Stockholm, Sweden (A.N., N.H.); University of Oslo, Norway (K.I.B.); Oslo University Hospital, Norway (K.I.B.); Steno Diabetes Center, Copenhagen, Gentofte, Denmark (M.E.J.); National Institute of Public Health, Southern Denmark University, Copenhagen (M.E.J.); Statisticon AB, Uppsala, Sweden (M.T.); AstraZeneca, Gaithersburg, MD (N.A.); AstraZeneca, Oslo, Norway (J.B.); AstraZeneca Gothenburg, Sweden (N.H.); and AstraZeneca, Cambridge, United Kingdom (P.F.).

**Keywords:** canagliflozin, dapagliflozin, death, diabetes mellitus, empagliflozin, heart failure, sodium glucose transporter 2

## Abstract

**Background:**

Reduction in cardiovascular death and hospitalization for heart failure (HHF) was recently reported with the sodium-glucose cotransporter-2 inhibitor (SGLT-2i) empagliflozin in patients with type 2 diabetes mellitus who have atherosclerotic cardiovascular disease. We compared HHF and death in patients newly initiated on any SGLT-2i versus other glucose-lowering drugs in 6 countries to determine if these benefits are seen in real-world practice and across SGLT-2i class.

**Methods:**

Data were collected via medical claims, primary care/hospital records, and national registries from the United States, Norway, Denmark, Sweden, Germany, and the United Kingdom. Propensity score for SGLT-2i initiation was used to match treatment groups. Hazard ratios for HHF, death, and their combination were estimated by country and pooled to determine weighted effect size. Death data were not available for Germany.

**Results:**

After propensity matching, there were 309 056 patients newly initiated on either SGLT-2i or other glucose-lowering drugs (154 528 patients in each treatment group). Canagliflozin, dapagliflozin, and empagliflozin accounted for 53%, 42%, and 5% of the total exposure time in the SGLT-2i class, respectively. Baseline characteristics were balanced between the 2 groups. There were 961 HHF cases during 190 164 person-years follow-up (incidence rate, 0.51/100 person-years). Of 215 622 patients in the United States, Norway, Denmark, Sweden, and the United Kingdom, death occurred in 1334 (incidence rate, 0.87/100 person-years), and HHF or death in 1983 (incidence rate, 1.38/100 person-years). Use of SGLT-2i, versus other glucose-lowering drugs, was associated with lower rates of HHF (hazard ratio, 0.61; 95% confidence interval, 0.51–0.73; *P*<0.001); death (hazard ratio, 0.49; 95% confidence interval, 0.41–0.57; *P*<0.001); and HHF or death (hazard ratio, 0.54; 95% confidence interval, 0.48–0.60; *P*<0.001) with no significant heterogeneity by country.

**Conclusions:**

In this large multinational study, treatment with SGLT-2i versus other glucose-lowering drugs was associated with a lower risk of HHF and death, suggesting that the benefits seen with empagliflozin in a randomized trial may be a class effect applicable to a broad population of patients with type 2 diabetes mellitus in real-world practice.

**Clinical Trial Registration:**

URL: http://www.clinicaltrials.gov. Unique identifier: NCT02993614.

## Abstract 2

Supplemental Digital Content is available in the text.

**Editorial, see p 260**

Type 2 diabetes mellitus (T2D) remains a major risk factor for cardiovascular disease (CVD)^1,2^ and overall mortality,^3,4^ despite advances in treatment.^5–7^ Heart failure is an especially common complication of T2D,^8–10^ with particularly poor outcomes and 5-year survival rates of <25%.^11^ This highlights the need for novel treatments that not only improve glycemic control, but also reduce the risk of CVD, including heart failure.

Although higher hemoglobin A1c is associated with greater risk of CVD,^12^ intensive glucose control has failed to reduce the development of heart failure, and cardiovascular-related or all-cause death. However, the EMPA-REG OUTCOME trial (BI 10773 [Empagliflozin] Cardiovascular Outcome Event Trial in Type 2 Diabetes Mellitus Patients), a prospective randomized controlled trial in patients with T2D and established atherosclerotic CVD, demonstrated a substantial reduction in cardiovascular death and hospitalization for heart failure (HHF) with the sodium-glucose cotransporter-2 inhibitor (SGLT-2i), empagliflozin,^13^ within a short follow-up period. The mechanisms of these benefits, although unclear, were almost certainly not attributable to glucose lowering, given a very small difference in hemoglobin A1c levels between empagliflozin- and placebo-treated patients and early separation of the event curves.

Following the EMPA-REG OUTCOMES trial, several critical questions remain, with substantial clinical implications. First, the applicability of findings to real-world clinical practice (where patients receive standard of care with various other glucose-lowering drugs [oGLDs]) is unclear. Second, it is unknown whether the observed benefits are specific to empagliflozin, or represent a class effect. Finally, because EMPA-REG OUTCOME only included patients with established CVD, it remains to be seen if similar benefits can be expected in patients with T2D who have a broader cardiovascular risk profile.

Using data from multiple countries in the CVD-REAL study (Comparative Effectiveness of Cardiovascular Outcomes in New Users of SGLT-2 Inhibitors: NCT02993614), we compared the risk for HHF, death, and the combined end point of HHF or death in patients with T2D who were new users of SGLT-2i versus oGLDs in real-world practice.

## Methods

### Data Sources

Deidentified health records across 6 countries (United States, Germany, Sweden, Norway, Denmark, and the United Kingdom) were analyzed. In the United States, Truven Health MarketScan Claims and Encounters and linked Medicare Supplemental and Coordination of Benefits databases were used, which included enrollment and demographic information, inpatient and outpatient medical, and outpatient pharmacy claims from >300 large self-insured US employers and >25 US health plans. In Germany, the Diabetes Prospective Follow-Up initiative is a quality assessment registry for individuals with diabetes mellitus and uses standardized documentation and objective comparison of quality indicators, with 452 centers participating. In Sweden, Norway, and Denmark, mandatory national full-population registries of each respective country were used, with linked Prescribed Drug Registers covering all drugs dispensed, National Patient Registers covering all hospitalizations and specialized outpatient care, and Cause of Death Registers.^14–17^ In the United Kingdom, records from the Clinical Practice Research Datalink and The Health Improvement Network data sets were used, which included primary care data from >670 general practices linked with hospitalization and mortality registries. Additional details of the individual data sets can be found in the online-only Data Supplement Appendix.

### Patient Cohort

Patients with T2D (diagnosis codes in online-only Data Supplement Tables I and II) who were newly started on either SGLT-2i or oGLDs were selected from each data set beginning on the date of first prescription or pharmacy dispensation of an SGLT-2i or a new oGLD in each of the countries (start date ranged from November 2012 in the United Kingdom to July 2013 in Sweden). New users were defined as individuals prescribed/filling a prescription (as initial or add-on therapy) for any SGLT-2i (canagliflozin, dapagliflozin, or empagliflozin) or oGLD (any other oral or injectable medication), including fixed-dose combinations, with no issued prescriptions of that medicine class during the preceding year (in Germany, with no prior documentation in the medical record of using that medicine class within the previous 6 months). Additional inclusion criteria were age ≥18 years on the index date (defined as the prescription date for new SGLT-2i or new oGLD), and >1 year data history in the database before the index date. Patients with type 1 or gestational diabetes were excluded. Patients were followed from the index date until the end of the index treatment (for the on-treatment analysis), migration/leaving the practice/database, last date of data collection, outcome date, or censoring date (range from September 2015 in the United States to November 2016 in Sweden).

### Outcomes

Primary outcome was HHF assessed in all countries. In the United States, United Kingdom, and Germany, HHF was defined as hospital admissions for heart failure (defined using primary discharge diagnosis codes in the United States,^18^ primary discharge diagnosis codes and documentation from the electronic health records in the United Kingdom, as defined in the online-only Data Supplement Appendix, and documentation in the electronic health records in Germany). In the Nordic countries (Sweden, Norway, and Denmark), HHF was defined by any hospital visit, in- or outpatient (ie, prognostically equivalent outpatient heart failure [HF] event),^19^ with a registered primary diagnosis of HF (defined using diagnosis codes for HF events as detailed in the online-only Data Supplement Appendix, and validated independently in all 3 countries).^20–22^ Secondary outcomes included all-cause death, and a composite of HHF or all-cause death (time-to-first-event), evaluated in all countries, except Germany. In the United States, all-cause death was identified by using the MarketScan Mortality File in which information from the Social Security Administration is integrated with the insurance enrollment and claims data, supplemented by claims for in-hospital deaths, covering ≈61% of the overall US-based propensity-matched patient cohort. Characteristics of US patients with and without vital status were similar (online-only Data Supplement Table III), indicating data missing completely at random because of administrative reasons.

### Statistical Analysis

Baseline characteristics of patients in the SGLT-2i and oGLD groups were analyzed by using descriptive statistics. Categorical variables were described by frequencies and percentages, and continuous variables by using mean (± SD). For continuous variables such as age, the overall mean across all databases was a summary estimate of country-specific means, weighted according to the number of patients in each respective database.

For the SGLT-2i group, the percentage of individual agents and their respective contributions to the overall SGLT-2i exposure time, and for the oGLD group, the percentage of individual drug classes, were summarized by country/geographic region and overall.

A nonparsimonious propensity score was developed (separately within each country) for being initiated on an SGLT-2i to minimize confounding. Variables that may have affected treatment assignment or outcomes were included in the propensity score (online-only Data Supplement Table IV).^23^ Based on propensity scores, patients receiving SGLT-2i were matched 1:1 with those receiving oGLDs. Nearest-neighbor caliper width of 0.25 multiplied by the SD of the propensity score distribution was used for the matching.^23^ In Sweden, Norway, and Denmark, an automated balance optimization method using the function Match (in package Matching) in R and a caliper of 0.2 were used for matching. The adequacy of propensity matching was assessed by standardized differences of postmatch patient characteristics. A significant imbalance was considered to be present if a >10% standardized difference was present between the 2 groups after propensity match.^24^

Incidence analyses of HHF, death, and composite of HHF or death were conducted by treatment group. Only the first episode of each outcome was included, and the crude incidence rate (IR) in each group was calculated as the number of incident events divided by the total number of person-years at risk, and expressed per 100 person-years with 95% confidence interval (CI). Times to first event for the SGLT-2i and oGLD groups were compared using Cox proportional hazards models and presented as hazard ratios (HRs) and 95% CI for each outcome separately within each country.

The primary analysis used an on-treatment approach where patients were followed from the start of an index treatment and censored at the end of that treatment plus a grace period (duration of last issued prescription).

The HRs (95% CI) for each of the end points from each individual country were then pooled together for an overall weighted summary,^25^ in which random-effects models with inverse variance weighting for each country were implemented.^26^ Forest plots displaying the country-specific HRs (95% CI) along with the pooled overall HR (95% CI) were produced.

Multiple sensitivity analyses were conducted: first, the HR (95% CI) within each country, and for each outcome, were examined after adjusting the crude propensity-matched estimates for multiple covariates that may have confounded the relationship between treatment and outcome. The adjusted HRs (95% CIs) from each country were pooled and meta-analyzed using the same method as described above. Second, the analyses for each outcome were repeated using intent-to-treat analysis, in which patients were followed after discontinuation of index treatment.^27–29^ Third, the analyses for HHF were repeated after stepwise removal of specific oGLD classes from the comparator group, to examine whether a specific oGLD class contributed disproportionately to the results. Stepwise elimination was performed in the following sequence: thiazolidinediones, thiazolidinediones+insulin, thiazolidinediones+insulin+sulfonylureas. Fourth, HHF analyses were repeated after excluding patients treated with glucagon-like peptide-1 receptor agonist at baseline from SGLT-2i and oGLD groups. Fifth, primary analyses were repeated separately in the United States and Europe. Finally, the association between treatment with SGLT-2i and oGLD was reexamined separately for patients that had both in- and outpatient hospital visits for HF, and those that had only inpatient hospital visits for HF in Sweden (because these could not be separated in Norway and Denmark; and only inpatient HF visits were analyzed in other countries).

For power calculations, see the online-only Data Supplement Appendix. Because of the deidentified nature of patient records, informed consent was not obtained. Analyses of deidentified data were conducted in accordance with local laws and regulations, and received approvals from respective Scientific/Ethics/Data Protection Committees. Country-specific analyses were conducted by independent academic/statistical groups. The meta-analyses were conducted by Statisticon, and validated by the independent academic statisticians at Saint Luke’s Mid America Heart Institute.

## Results

### Study Population

**Figure 1.**
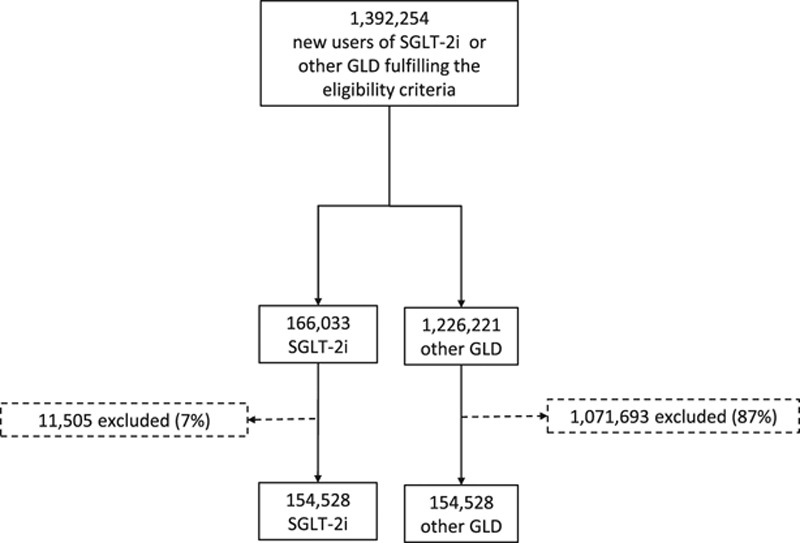
**Patient flow chart for all countries/databases combined.** A large number of patients were excluded from the other GLD group because of the protocol mandated 1:1 match, and given the smaller number of patients in the SGLT-2i group. GLD indicates glucose-lowering drug; and SGLT-2i, sodium-glucose cotransporter-2 inhibitor.

A total of 1 392 254 new SGLT-2i or oGLD users were identified; 166 033 SGLT-2i, and 1 226 221 oGLD overall and by country (Figure 1 and online-only Data Supplement Figure I). Before propensity match (online-only Data Supplement Table V) patients initiated on SGLT-2i were younger, less likely to have chronic kidney disease or cardiovascular complications, but more likely to have microvascular disease. Greater proportions of patients initiated on SGLT-2i versus oGLD received statins and antihypertensive drugs, and lower proportions received loop diuretics. Patients on SGLT-2i were more likely to be treated with other glucose-lowering medication classes at baseline. The overlap in propensity scores between groups before and after the propensity match is shown in online-only Data Supplement Figures II and III.

Baseline characteristics were well balanced between groups postmatching overall and by country (Table and online-only Data Supplement Table VI), with standardized differences for most variables being <10% (online-only Data Supplement Figure IV). Pre- and postmatch standardized differences are shown in online-only Data Supplement Table VII. Mean age was 57 years, 44% were women, and 13% had established CVD. Overall, 67% of patients received statins, 80% antihypertensive medications, 74% with angiotensin-converting enzyme inhibitors/angiotensin II receptor blockers, and 79% metformin.

**Table.**
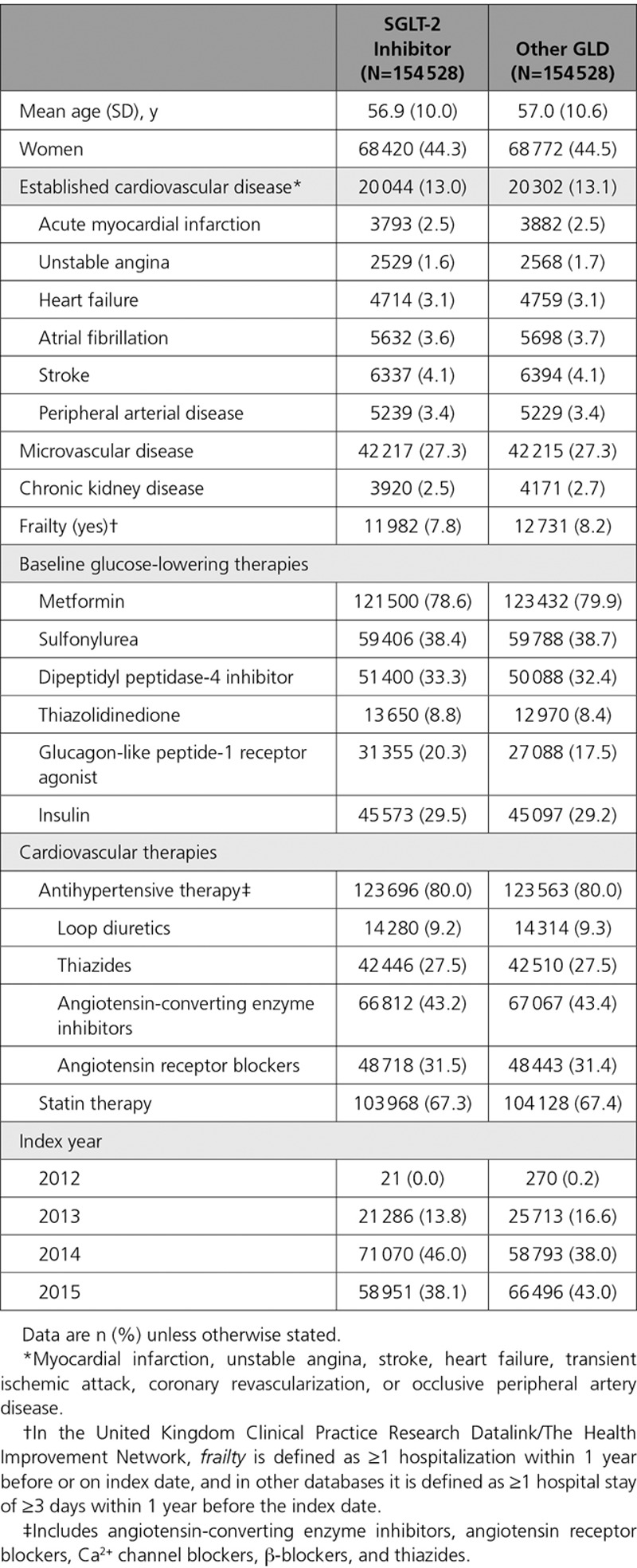
Baseline Characteristics for All Countries Combined

**Figure 2.**
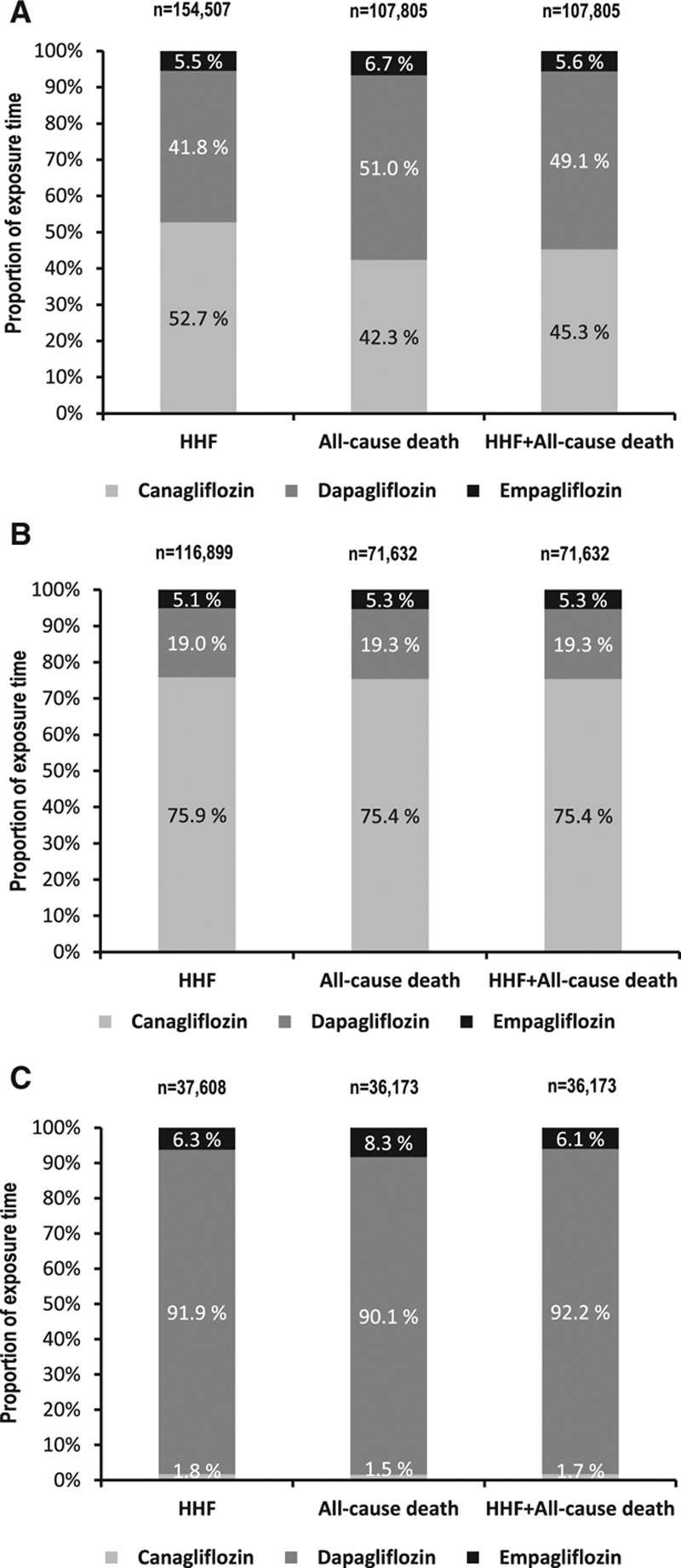
**Contribution of the SGLT-2 inhibitor class as a proportion of exposure time in the propensity-match cohorts. A**, All countries combined. **B**, United States only. **C**, European countries combined. HHF indicates hospitalization for heart failure; and SGLT-2, sodium-glucose cotransporter-2.

The composition of SGLT-2i agents is shown in online-only Data Supplement Table VIII, and the composition of the index medications in the oGLD group is shown in online-only Data Supplement Tables IX and X. The composition of SGLT-2i agents in terms of total exposure time was balanced between canagliflozin and dapagliflozin, with <7% total exposure attributable to empagliflozin for all outcomes (Figure 2).

### SGLT-2i and HHF

A total of 309 056 patients (154 528 in each group) were identified after propensity matching. Canagliflozin, dapagliflozin, and empagliflozin accounted for 53%, 42%, and 5% of the total exposure time in the SGLT-2i class, respectively (Figure 2A through 2C).

**Figure 3.**
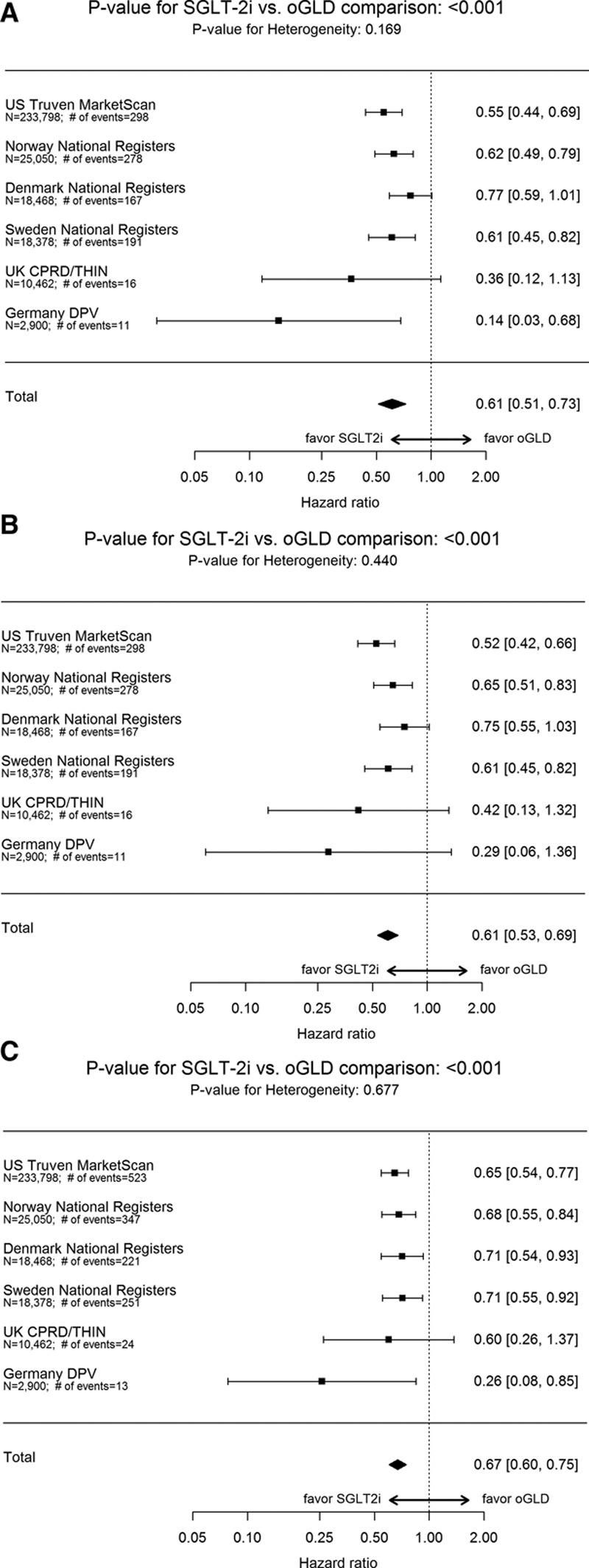
**Hazard ratios and 95% CI for the outcome of HHF.** **A**, On treatment, unadjusted. **B**, On treatment, adjusted (model adjusted for history of heart failure, age, sex, frailty, history of myocardial infarction, history of atrial fibrillation, hypertension, obesity/body mass index, duration of diabetes mellitus, ACE inhibitor or ARB use, β-blocker or α-blocker use, Ca2^+^ channel blocker use, loop diuretic use, thiazide diuretic use). **C**, Intent-to-treat, unadjusted. ACE indicates angiotensin-converting enzyme; ARB, angiotensin II receptor blocker; CI, confidence interval; CPRD, Clinical Practice Research Datalink; DPV, Diabetes Patientenverlaufsdokumentation (Diabetes Prospective Follow-Up); HHF, hospitalization for heart failure; oGLD, other glucose-lowering drugs; SGLT-2i, sodium-glucose cotransporter-2 inhibitor; and THIN, The Health Improvement Network.

Over 190 164 person-years follow-up, there were 961 HHF events (IR, 0.51/100 person-years; online-only Data Supplement Table XI; IR by treatment group in online-only Data Supplement Table XII). Mean duration of follow-up for HHF was 239 days in the SGLT-2i group and 211 days in the oGLD group (online-only Data Supplement Table XIII). Initiation of SGLT-2i versus oGLD was associated with a lower risk of HHF (pooled HR, 0.61; 95% CI, 0.51–0.73; *P*<0.001; Figure 3A). HRs favored SGLT-2i in each country (*P* value for heterogeneity 0.17).

### SGLT-2i and All-Cause Death

A total of 215 622 patients (107 811 in each group) were identified. Canagliflozin, dapagliflozin, and empagliflozin accounted for 42%, 51%, and 7% of SGLT-2i exposure time, respectively (Figure 2A through 2C).

**Figure 4.**
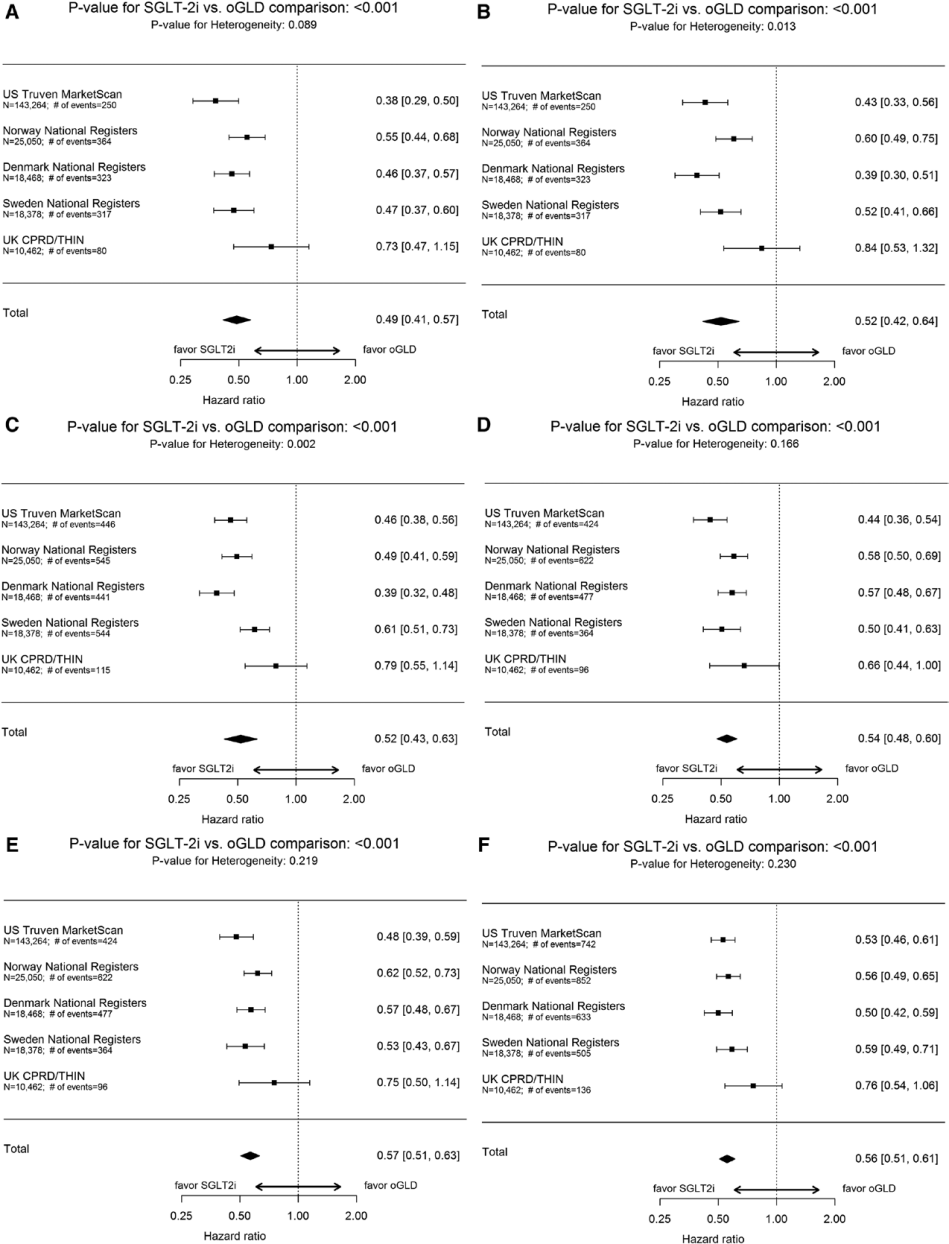
**Hazard ratios and 95% CI for the outcome of all-cause death and composite of hospitalization for heart failure or all-cause death.** **A**, All-cause death: on treatment, unadjusted. **B**, All-cause death: on treatment, adjusted (model adjusted for history of heart failure, age, sex, frailty, history of myocardial infarction, history of atrial fibrillation, hypertension, obesity/body mass index, duration of diabetes mellitus, ACE inhibitor or ARB use, β-blocker or α-blocker use, Ca2^+^ channel blocker use, loop diuretic use, thiazide diuretic use). **C**, All-cause death: intent-to-treat, unadjusted. **D**, Hospitalization for heart failure or all-cause death: on treatment, unadjusted. **E**, Hospitalization for heart failure or all-cause death: on treatment, adjusted (model adjusted for history of heart failure, age, sex, frailty, history of myocardial infarction, history of atrial fibrillation, hypertension, obesity/body mass index, duration of diabetes mellitus, ACE inhibitor or ARB use, β-blocker or α-blocker use, Ca2^+^ channel blocker use, loop diuretic use, thiazide diuretic use). **F**, Hospitalization for heart failure or all-cause death: intent-to-treat, unadjusted. ACE indicates angiotensin-converting enzyme; ARB, angiotensin II receptor blocker; CI, confidence interval; CPRD, Clinical Practice Research Datalink; DPV, Diabetes Patientenverlaufsdokumentation (Diabetes Prospective Follow-Up); oGLD, other glucose-lowering drugs; SGLT-2i, sodium-glucose cotransporter-2 inhibitor; and THIN, The Health Improvement Network.

Over 153 990 person-years of follow-up, there were 1334 events (IR, 0.87/100 person-years; online-only Data Supplement Table XI; IR by treatment group in online-only Data Supplement Table XII). Mean duration of follow-up was 271 days in the SGLT-2i group and 251 days in the oGLD group (online-only Data Supplement Table XIII). Initiation of SGLT-2i versus oGLD was associated with a lower risk of death (pooled HR, 0.49; 95% CI, 0.41–0.57; *P*<0.001; Figure 4A). HRs favored SGLT-2i in each country (*P* value for heterogeneity 0.09).

### SGLT-2i and Composite Outcome of HHF or Death

For the composite outcome, the number of patients was identical to the all-cause death analysis. Canagliflozin, dapagliflozin, and empagliflozin accounted for 45%, 49%, and 6% of SGLT-2i exposure time, respectively (Figure 2A through 2C).

Over 143 342 person-years of follow-up, there were 1983 events (IR, 1.38/100 person-years; online-only Data Supplement Table XI; IR by treatment group in online-only Data Supplement Table XII). Mean duration of follow-up was 253 days in the SGLT-2i group and from 233 days in the oGLD group (online-only Data Supplement Table XIII). Initiation of SGLT-2i versus oGLD was associated with a lower risk of HHF or death (pooled HR, 0.54; 95% CI, 0.48–0.60; *P*<0.001; Figure 4D). HRs favored SGLT-2i in each country (*P* value for heterogeneity 0.17).

### Sensitivity Analyses

For all 3 outcomes, similar results were found after multivariate adjustment (Figures 3B, 4B, and 4E), using an intent-to-treat approach (Figures 3C, 4C, and 4F) and stepwise removal of specific oGLD classes (online-only Data Supplement Figures V and VI). Comparisons within geographic regions yielded similar results (online-only Data Supplement Figure VII). The association between treatment with SGLT-2i versus oGLD and lower risk of HHF was consistent among patients that had both in- and outpatient hospital visits for HF, and those who had only inpatient hospital visits for HF in Sweden (online-only Data Supplement Table XIV).

## Discussion

In this large contemporary analysis of real-world clinical practice across 6 countries, within a well-matched sample of >300 000 patients with T2D and nearly 200 000 patient-years of observation, initiation of SGLT-2i versus oGLDs was associated with a 39% lower incidence of HHF. Since the overwhelming majority of patients did not have established CVD, this suggests that the benefits of SGLT-2i on the prevention of HF may extend to lower-risk patients than those enrolled in randomized trials so far. These findings were unchanged after additional multivariable adjustment, and in multiple sensitivity analyses. Specifically, the results were unchanged after sequential removal of several oGLD classes from the comparator group, suggesting that the differential outcomes observed are unlikely to reflect adverse effects of comparator drugs, but are rather associated with benefit from SGLT-2i. Furthermore, results were consistent across countries, regardless of variability in healthcare systems and use of specific SGLT-2i (predominantly canagliflozin in the United States; dapagliflozin in Europe), suggesting an association with the class rather than any single agent. Importantly, initiation of SGLT-2i versus oGLDs was also associated with a 51% lower rate of all-cause death, and a 46% lower rate of the combined end point of HHF or all-cause death.

Although intensive glucose lowering has, in randomized trials, failed to reduce what are arguably some of the most important outcomes in patients with T2D (all-cause death and incident HF), results from the EMPA-REG OUTCOME trial demonstrated that such benefits are achievable within a short time frame with an SGLT-2i, likely via nonglycemic mechanisms. Ultimately, the main goals of treating patients with T2D are to prolong life and improve quality of life. Given that CVD (including HF) is a leading cause of mortality/morbidity in T2D, the results of the recent cardiovascular outcomes trials suggest that the time has come to shift from the narrow focus on hemoglobin A1c to a more comprehensive focus in which treatments proven to improve important outcomes (especially mortality) are prioritized.

Our findings address several key unanswered questions with regard to the potential role of SGLT-2i in the management of T2D, with important clinical implications. First, our results demonstrate that the effects associated with the use of SGLT-2i in regard to HHF and all-cause death are remarkably similar in real-world practice to those seen in the EMPA-REG OUTCOME trial. Second, we found no significant heterogeneity in results across countries, despite geographic variations in the use of specific SGLT-2i, suggesting that the associated lower risks for cardiovascular outcomes are likely class related. Indeed, for all outcomes evaluated, empagliflozin contributed <7% of total exposure time. Third, we evaluated a broader cardiovascular risk population in general practice, where the overwhelming majority (87%) had no established CVD, suggesting that lower-risk patients may derive benefits with SGLT-2i similar to the patients with higher risk. If confirmed by data from ongoing trials (CANVAS^30^ [Canagliflozin Cardiovascular Assessment Study; NCT01032629]; DECLARE [Multicenter Trial to Evaluate the Effect of Dapagliflozin on the Incidence of Cardiovascular Events; NCT01730534]; and VERTIS [Cardiovascular Outcomes Following Ertugliflozin Treatment in Type 2 Diabetes Mellitus Participants With Vascular Disease, The VERTIS CV Study {MK-8835-004}; NCT01986881]), this would have substantial impact on clinical practice. In this regard, we see the data produced from carefully conducted, methodologically rigorous, large multicountry epidemiological studies, as complementary to those generated by clinical trials, as they help establish the real-world effectiveness of treatments in a broad population of patients from clinical practice. Indeed, the importance of such studies for a broad range of objectives, including evaluation of treatment effects and outcomes, and their potential for complementing the knowledge gained from clinical trials, is being increasingly recognized,^31^ and the terminology describing these as real-world evidence has recently been accepted by major international regulatory bodies.^32–34^

To our knowledge, CVD-REAL is the first large study addressing the real-world effectiveness (rather than efficacy) of SGLT-2i on specific outcomes of HHF and all-cause death across multiple countries. Given that SGLT-2i is a novel class, real-world experience is limited. Single-country data were previously reported with a specific SGLT-2i (dapagliflozin) in Sweden; however, that study was limited by the smaller number of patients, and focused on different outcomes (hypoglycemia and composite of CVD).^35^ The size of our sample and our ability to pool data from diverse sources allowed us to collect a large number of events, and examine the stability of results across various cardiovascular outcomes, multiple countries (with variable use of specific SGLT-2i), and perform numerous sensitivity analyses.

Our findings should be examined within the context of several potential limitations. First, given the observational nature of the study, and despite robust propensity-matching and multiple sensitivity analyses, a possibility of residual, unmeasured confounding, cannot be excluded. Second, we focused on HHF and all-cause death, and did not examine other events, such as myocardial infarction and stroke. However, HF is arguably the most lethal T2D complication^9,36^ and is associated with particularly poor survival.^11^ Third, we did not examine safety. Fourth, despite a large number of patient-years of follow up, SGLT-2i experience in real-world practice is still relatively limited; longer-term follow-up will be required to examine if effects are sustained over time. Fifth, there were differences in the definitions of HHF across countries; however, the results were consistent across countries and in sensitivity analyses specifically performed to examine these differences. Finally, our study did not address the mechanisms linking the use of SGLT-2i and associated cardiovascular benefits. However, this knowledge gap is being examined by mechanistic investigations across the class.^37–39^

## Conclusion

In this large multinational study, treatment with SGLT-2i versus oGLDs was associated with lower rates of HHF and death, suggesting that the benefits previously reported with empagliflozin in the context of a randomized trial may be applicable to a broad population of patients with T2D in real-world practice. The lack of heterogeneity in results across countries, despite geographic variations in the use of specific SGLT-2i, suggests a class effect for SGLT-2i.

## Acknowledgments

The authors acknowledge Betina T. Blak, Sara E. Dempster, Markus F. Scheerer, Karolina Andersson-Sundell, Kelly Bell, Eric T. Wittbrodt, Luis Alberto García Rodríguez, Lucia Cea Soriano, Oscar Fernándex Cantero, Ellen Riehle, Brian Murphy, Esther Bollow, Hanne Løvdal Gulseth, Bendix Carstensen, Fengming Tang, Kevin Kennedy, and Sheryl L Windsor for their tireless contribution to the country-level analyses, quality check validation, and results interpretation. Data validation was independently conducted by MAHI, an external academic institution. All authors had access to the full data package and are responsible for data interpretation and conclusions. Additional details on databases and sensitivity analyses are provided in the online-only Data Supplement. Editorial support was provided by Róisín O’Connor and Mark Davies, inScience Communications, Springer Healthcare, and funded by AstraZeneca.

## Sources of Funding

This work was supported by AstraZeneca.

## Disclosures

Dr Kosiborod has served advisory boards for AstraZeneca, Boehringer Ingelheim, Sanofi, Glytec, Novo Nordisk, ZS Pharma, GSK, Amgen, Eisai and Merck (Diabetes); is a consultant for AstraZeneca, Sanofi, and ZS Pharma; and received research grants from AstraZeneca and Boehringer Ingelheim. Dr Cavender received personal fees from Merck and AstraZeneca. Dr Fu received grants from AstraZeneca and Merck; and personal fees from Asclepius Analytics and Complete HEOR Services. Dr Wilding received lecture fees from Astellas, AstraZeneca, Boehringer Ingelheim, Janssen, Lilly, Novo Nordisk, Orexigen, Sanofi; and consultancy (Institutional) from AstraZeneca, Boehringer Ingelheim, Janssen, Lilly, and Orexigen; and grants to institution from Takeda, Novo Nordisk and AstraZeneca. Dr Khunti serves as consultant and speaker for AstraZeneca, Novartis, Novo Nordisk, Sanofi-Aventis, Lilly, Merck Sharp & Dohme, Janssen and Boehringer Ingelheim; and received grants in support of investigator and investigator initiated trials from AstraZeneca, Novartis, Novo Nordisk, Sanofi-Aventis, Lilly, Boehringer Ingelheim, Merck Sharp & Dohme and Roche; and served on advisory boards for AstraZeneca, Novartis, Novo Nordisk, Sanofi-Aventis, Lilly, Merck Sharp & Dohme, Janssen, and Boehringer Ingelheim. Dr Holl received grants from AstraZeneca. Dr Norhammar received personal fees from AstraZeneca for this study; and honoraria for lectures and advisory board meetings for Novo Nordisk, Boehringer Ingelheim, and Lilly. Dr Birkeland received grants to his institution from AstraZeneca for this study. Dr Jørgensen is a shareholder of Novo Nordisk, was employed by Steno Diabetes Center A/S until December 31, 2016, a research hospital working in the Danish National Health Service and owned by Novo Nordisk A/S; received grants from AstraZeneca and for lectures and consulting from Novo Nordisk, Sanofi, Lilly, Boehringer Ingelheim, and Merck Sharp & Dohme. Dr Thuresson is an employee of Statisticon who were under contract to AstraZeneca for this study. N. Arya and Drs Bodegard, Hammar, and Fenicii are employees of AstraZeneca.

## Supplementary Material

## Clinical Perspective

### What Is New?

- This is the first large real-world study of >300 000 patients with type 2 diabetes mellitus, both with and without established cardiovascular disease, from routine clinical practice across 6 countries, evaluating the outcomes of hospitalization for heart failure (HHF) and all-cause death in patients with type 2 diabetes mellitus treated with sodium-glucose cotransporter-2 inhibitors (SGLT-2i) versus other glucose-lowering drugs.
- The distribution of exposure time for the various SGLT-2i compounds (for HHF outcome) was 53% for canagliflozin, 42% for dapagliflozin, and ≈5% for empagliflozin, with substantial intercountry variability.

### What Are the Clinical Implications?

- Treatment with SGLT-2i versus other glucose-lowering drugs was associated with a 39% relative risk reduction in HHF, a 51% reduction in all-cause death, and a 46% reduction in the HHF or death composite, consistent with the effects previously reported in a randomized clinical trial of empagliflozin.
- Approximately 87% of patients did not have known cardiovascular disease, suggesting possible cardiovascular benefits for a broad population of patients with type 2 diabetes mellitus.
- The lower rates of HHF and death associated with SGLT-2i treatment are likely class related, as there was no significant heterogeneity across countries, despite geographic variations in the use of specific SGLT-2i (≈76% canagliflozin in the United States and ≈92% dapagliflozin in Europe).

## References

[1] Sarwar N, Gao P, Seshasai SR, Gobin R, Kaptoge S, Di Angelantonio E, Ingelsson E, Lawlor DA, Selvin E, Stampfer M, Stehouwer CD, Lewington S, Pennells L, Thompson A, Sattar N, White IR, Ray KK, Danesh J, Emerging Risk Factors Collaboration (2010). Diabetes mellitus, fasting blood glucose concentration, and risk of vascular disease: a collaborative meta-analysis of 102 prospective studies.. Lancet.

[2] Schocken DD, Benjamin EJ, Fonarow GC, Krumholz HM, Levy D, Mensah GA, Narula J, Shor ES, Young JB, Hong Y, American Heart Association Council on Epidemiology and Prevention; American Heart Association Council on Clinical Cardiology; American Heart Association Council on Cardiovascular Nursing; American Heart Association Council on High Blood Pressure Research; Quality of Care and Outcomes Research Interdisciplinary Working Group; Functional Genomics and Translational Biology Interdisciplinary Working Group (2008). Prevention of heart failure: a scientific statement from the American Heart Association Councils on Epidemiology and Prevention, Clinical Cardiology, Cardiovascular Nursing, and High Blood Pressure Research; Quality of Care and Outcomes Research Interdisciplinary Working Group; and Functional Genomics and Translational Biology Interdisciplinary Working Group.. Circulation.

[3] Di Angelantonio E, Kaptoge S, Wormser D, Willeit P, Butterworth AS, Bansal N, O’Keeffe LM, Gao P, Wood AM, Burgess S, Freitag DF, Pennells L, Peters SA, Hart CL, Håheim LL, Gillum RF, Nordestgaard BG, Psaty BM, Yeap BB, Knuiman MW, Nietert PJ, Kauhanen J, Salonen JT, Kuller LH, Simons LA, van der Schouw YT, Barrett-Connor E, Selmer R, Crespo CJ, Rodriguez B, Verschuren WM, Salomaa V, Svärdsudd K, van der Harst P, Björkelund C, Wilhelmsen L, Wallace RB, Brenner H, Amouyel P, Barr EL, Iso H, Onat A, Trevisan M, D’Agostino RB, Cooper C, Kavousi M, Welin L, Roussel R, Hu FB, Sato S, Davidson KW, Howard BV, Leening MJ, Leening M, Rosengren A, Dörr M, Deeg DJ, Kiechl S, Stehouwer CD, Nissinen A, Giampaoli S, Donfrancesco C, Kromhout D, Price JF, Peters A, Meade TW, Casiglia E, Lawlor DA, Gallacher J, Nagel D, Franco OH, Assmann G, Dagenais GR, Jukema JW, Sundström J, Woodward M, Brunner EJ, Khaw KT, Wareham NJ, Whitsel EA, Njølstad I, Hedblad B, Wassertheil-Smoller S, Engström G, Rosamond WD, Selvin E, Sattar N, Thompson SG, Danesh J, Emerging Risk Factors Collaboration (2015). Association of cardiometabolic multimorbidity with mortality.. JAMA.

[4] Kannel WB, Hjortland M, Castelli WP (1974). Role of diabetes in congestive heart failure: the Framingham study.. Am J Cardiol.

[5] IDF Diabetes Atlas Group (2013). Update of mortality attributable to diabetes for the IDF diabetes atlas: estimates for the year 2011.. Diabetes Res Clin Pract.

[6] NCD Risk Factor Collaboration (NCD-RisC) (2016). Worldwide trends in diabetes since 1980: a pooled analysis of 751 population-based studies with 4.4 million participants.. Lancet.

[7] Tancredi M, Rosengren A, Svensson AM, Kosiborod M, Pivodic A, Gudbjörnsdottir S, Wedel H, Clements M, Dahlqvist S, Lind M (2015). Excess mortality among persons with type 2 diabetes.. N Engl J Med.

[8] Cavender MA, Steg PG, Smith SC, Eagle K, Ohman EM, Goto S, Kuder J, Im K, Wilson PW, Bhatt DL, REACH Registry Investigators (2015). Impact of diabetes mellitus on hospitalization for heart failure, cardiovascular events, and death: outcomes at 4 years from the Reduction of Atherothrombosis for Continued Health (REACH) Registry.. Circulation.

[9] Fitchett D, Zinman B, Wanner C, Lachin JM, Hantel S, Salsali A, Johansen OE, Woerle HJ, Broedl UC, Inzucchi SE, EMPA-REG OUTCOME® trial investigators (2016). Heart failure outcomes with empagliflozin in patients with type 2 diabetes at high cardiovascular risk: results of the EMPA-REG OUTCOME® trial.. Eur Heart J.

[10] Green JB, Bethel MA, Armstrong PW, Buse JB, Engel SS, Garg J, Josse R, Kaufman KD, Koglin J, Korn S, Lachin JM, McGuire DK, Pencina MJ, Standl E, Stein PP, Suryawanshi S, Van de Werf F, Peterson ED, Holman RR (2015). Effect of sitagliptin on cardiovascular outcomes in type 2 diabetes.. N Engl J Med.

[11] Bertoni AG, Hundley WG, Massing MW, Bonds DE, Burke GL, Goff DC (2004). Heart failure prevalence, incidence, and mortality in the elderly with diabetes.. Diabetes Care.

[12] Matsushita K, Blecker S, Pazin-Filho A, Bertoni A, Chang PP, Coresh J, Selvin E (2010). The association of hemoglobin A1c with incident heart failure among people without diabetes: the atherosclerosis risk in communities study.. Diabetes.

[13] Zinman B, Wanner C, Lachin JM, Fitchett D, Bluhmki E, Hantel S, Mattheus M, Devins T, Johansen OE, Woerle HJ, Broedl UC, Inzucchi SE, EMPA-REG OUTCOME Investigators (2015). Empagliflozin, cardiovascular outcomes, and mortality in type 2 diabetes.. N Engl J Med.

[14] Håheim LL, Helgeland J (2014). Agreement between referral information and discharge diagnoses according to Norwegian elective treatment guidelines - a cross-sectional study.. BMC Health Serv Res.

[15] Ludvigsson JF, Andersson E, Ekbom A, Feychting M, Kim JL, Reuterwall C, Heurgren M, Olausson PO (2011). External review and validation of the Swedish national inpatient register.. BMC Public Health.

[16] Norhammar A, Bodegard J, Nystrom T, Thuresson M, Eriksson JW, Nathanson D (2016). Incidence, prevalence and mortality of type 2 diabetes requiring glucose-lowering treatment, and associated risks of cardiovascular complications: a nationwide study in Sweden, 2006–2013.. Diabetologia.

[17] Sundbøll J, Adelborg K, Munch T, Frøslev T, Sørensen HT, Bøtker HE, Schmidt M (2016). Positive predictive value of cardiovascular diagnoses in the Danish National Patient Registry: a validation study.. BMJ Open.

[18] Lee DS, Donovan L, Austin PC, Gong Y, Liu PP, Rouleau JL, Tu JV (2005). Comparison of coding of heart failure and comorbidities in administrative and clinical data for use in outcomes research.. Med Care.

[19] Okumura N, Jhund PS, Gong J, Lefkowitz MP, Rizkala AR, Rouleau JL, Shi VC, Swedberg K, Zile MR, Solomon SD, Packer M, McMurray JJ, PARADIGM-HF Investigators and Committees* (2016). Importance of clinical worsening of heart failure treated in the outpatient setting: evidence from the Prospective Comparison of ARNI With ACEI to Determine Impact on Global Mortality and Morbidity in Heart Failure Trial (PARADIGM-HF).. Circulation.

[20] Brynildsen J, Høiseth AD, Nygård S, Hagve TA, Christensen G, Omland T, Røsjø H (2015). [Diagnostic accuracy for heart failure – data from the Akershus Cardiac Examination 2 Study].. Tidsskr Nor Laegeforen.

[21] Kümler T, Gislason GH, Kirk V, Bay M, Nielsen OW, Køber L, Torp-Pedersen C (2008). Accuracy of a heart failure diagnosis in administrative registers.. Eur J Heart Fail.

[22] Ingelsson E, Arnlöv J, Sundström J, Lind L (2005). The validity of a diagnosis of heart failure in a hospital discharge register.. Eur J Heart Fail.

[23] Stuart EA (2010). Matching methods for causal inference: a review and a look forward.. Stat Sci.

[24] Normand ST, Landrum MB, Guadagnoli E, Ayanian JZ, Ryan TJ, Cleary PD, McNeil BJ (2001). Validating recommendations for coronary angiography following acute myocardial infarction in the elderly: a matched analysis using propensity scores.. J Clin Epidemiol.

[25] DerSimonian R, Laird N (1986). Meta-analysis in clinical trials.. Control Clin Trials.

[26] Viechtbauer W (2010). Conducting meta-analyses in r with the metafor package.. J Stat Softw.

[27] Hernán MA, Alonso A, Logan R, Grodstein F, Michels KB, Willett WC, Manson JE, Robins JM (2008). Observational studies analyzed like randomized experiments: an application to postmenopausal hormone therapy and coronary heart disease.. Epidemiology.

[28] Hernán MA, Hernández-Díaz S (2012). Beyond the intention-to-treat in comparative effectiveness research.. Clin Trials.

[29] Prentice RL, Pettinger M, Anderson GL (2005). Statistical issues arising in the Women’s Health Initiative.. Biometrics.

[30] Neal B, Perkovic V, Mahaffey KW, Fulcher G, Erondu N, Desai M, Shaw W, Law G, Walton MK, Rosenthal N, de Zeeuw D, Matthews DR, on behalf of the CANVAS Program collaborative group (2017). Optimising the analysis strategy for the canvas program – a pre-specified plan for the integrated analyses of the canvas and canvas-r trials.. Diabetes Obes Metab.

[31] Sherman RE, Anderson SA, Dal Pan GJ, Gray GW, Gross T, Hunter NL, LaVange L, Marinac-Dabic D, Marks PW, Robb MA, Shuren J, Temple R, Woodcock J, Yue LQ, Califf RM (2016). Real-world evidence - what is it and what can it tell us?. N Engl J Med.

[32] U.S. Department of Health and Human Services, Food and Drug Administration, Center for Devices and Radiological Health, Center for Biologics Evaluation and Research Use of Real-World Evidence to Support Regulatory Decision-Making for Medical Devices. Draft Guidance for Industry and Food and Drug Administration Staff.

[33] European Commission, Directorate-General for Health and Food Safety STAMP Commission Expert Group. Real world evidence.. http://ec.europa.eu/health//sites/health/files/files/committee/stamp/2016-03_stamp4/4_real_world_evidence_background_paper.pdf.

[34] Califf RM, Robb MA, Bindman AB, Briggs JP, Collins FS, Conway PH, Coster TS, Cunningham FE, De Lew N, DeSalvo KB, Dymek C, Dzau VJ, Fleurence RL, Frank RG, Gaziano JM, Kaufmann P, Lauer M, Marks PW, McGinnis JM, Richards C, Selby JV, Shulkin DJ, Shuren J, Slavitt AM, Smith SR, Washington BV, White PJ, Woodcock J, Woodson J, Sherman RE (2016). Transforming evidence generation to support health and health care decisions.. N Engl J Med.

[35] Nystrom T, Bodegard J, Nathanson D, Thuresson M, Norhammar A, Eriksson JW Novel oral glucose-lowering drugs compared to insulin are associated with lower risk of all-cause mortality, cardiovascular events and severe hypoglycemia in type 2 diabetes patients [published online ahead of print January 24, 2017].. Diabetes Obes Metab.

[36] Scirica BM, Bhatt DL, Braunwald E, Steg PG, Davidson J, Hirshberg B, Ohman P, Frederich R, Wiviott SD, Hoffman EB, Cavender MA, Udell JA, Desai NR, Mosenzon O, McGuire DK, Ray KK, Leiter LA, Raz I, SAVOR-TIMI 53 Steering Committee and Investigators (2013). Saxagliptin and cardiovascular outcomes in patients with type 2 diabetes mellitus.. N Engl J Med.

[37] Heerspink HJ, Perkins BA, Fitchett DH, Husain M, Cherney DZ (2016). Sodium glucose cotransporter 2 inhibitors in the treatment of diabetes mellitus: cardiovascular and kidney effects, potential mechanisms, and clinical applications.. Circulation.

[38] Marx N, McGuire DK (2016). Sodium-glucose cotransporter-2 inhibition for the reduction of cardiovascular events in high-risk patients with diabetes mellitus.. Eur Heart J.

[39] Sattar N, McLaren J, Kristensen SL, Preiss D, McMurray JJ (2016). SGLT2 Inhibition and cardiovascular events: why did EMPA-REG Outcomes surprise and what were the likely mechanisms?. Diabetologia.

