# Supplementary material for: Lower Risk of Heart Failure and Death in Patients Initiated on Sodium-Glucose Cotransporter-2 Inhibitors Versus Other Glucose-Lowering Drugs: The CVD-REAL Study (Comparative Effectiveness of Cardiovascular Outcomes in New Users of Sodium-Glucose Cotransporter-2 Inhibitors)
Source: Circulation. 2017 May 18;136(3):249–59. doi: 10.1161/CIRCULATIONAHA.117.029190 (PMC5515629; doi:10.1161/CIRCULATIONAHA.117.029190)
Supplement: Supplementary file 1 [file cir-136-249-s001.pdf]

SUPPLEMENTAL MATERIAL

Table of Contents

CVD-REAL Investigator and Study Group 4

Characteristics of the databases 5

Statistical power calculations 8

SUPPLEMENTARY TABLES

Table S1. ICD and Read codes for Type 2 diabetes, Type 1 diabetes and gestational diabetes 9

Table S2. Read codes for history of cardiovascular events 17

Table S3. Comparison of post-propensity match baseline characteristics between the US Truven MarketScan mortality subset and US Truven MarketScan total population 36

Table S4. List of variables used to develop propensity score 38

Table S5. Baseline characteristics for all countries/databases pre-match 40

Table S6. Baseline characteristics for all countries/databases post-match 42

Table S7: Standardized differences in baseline characteristics between SGLT-2 inhibitor and other GLD treatment groups pre- and post-propensity match by country 44

Table S8. Composition of SGLT-2 inhibitor class in propensity matched cohorts 48

Table S9. Index glucose-lowering medication classes for patients in the other GLD group: all countries combined (HHF analysis) 49

Table S10. Index glucose-lowering medication classes for patients in the other GLD group: all countries combined (all-cause death analysis and HHF or all-cause death analysis) 50

Table S11: Number of patients, person-years at risk (on treatment), events and events/100 person-years (incidence rates) for each of the endpoints 51

Table S12. Pooled event rates by treatment groups 52

Table S13. Mean follow-up time (in days) by country and endpoint 53

Table S14. Sensitivity analyses examining association between treatment with SGLT-2i vs. oGLD and outcomes of HHF in Sweden, among patients with both in- and outpatient hospital visits with primary diagnosis of heart failure, and those with only inpatient hospital visits with the primary diagnosis of heart failure 54

SUPPLEMENTARY FIGURES

Figure S1: Patient selection flow-charts for each country 55

A. US Truven MarketScan

B. Norway National Registers
C. Denmark National Registers
D. Sweden National Registers
E. UK CPRD/THIN
F. Germany

Figure S2: Propensity score distribution by country pre-match 58

A. US Truven MarketScan
B. Norway National Registers
C. Denmark National Registers
D. Sweden National Registers
E. UK CPRD/THIN
F. Germany

Figure S3: Propensity score distribution by country post-match 59

A. US Truven MarketScan
B. Norway National Registers
C. Denmark National Registers
D. Sweden National Registers
E. UK CPRD/THIN
F. Germany

Figure S4: Standardized differences between SGLT-2 inhibitor and other GLD treatment groups by country 60

A. US Truven MarketScan
B. Norway National Registers
C. Denmark National Registers
D. Sweden National Registers
E. UK CPRD/THIN
F. Germany

Figure S5: Stepwise sensitivity analysis (sequentially removing comparators): Outcome of hospitalization for heart failure 66

A. TZD removed
B. TZD and insulin removed
C. TZD, insulin and SU removed

Figure S6: Outcome of hospitalization for heart failure excluding patients with baseline GLP-1 RAs 69

Figure S7: Outcomes of hospitalization for heart failure, all-cause death, and hospitalization for heart failure or all-cause for the SGLT-2 inhibitor versus other GLD treatment groups: 70

A. US only
B. European countries combined

References for the supplementary appendices 71

CVD-REAL Investigator and Study Group

Executive Scientific Committee (Academic Members and Investigators):

Mikhail Kosiborod, MD, Saint Luke's Mid America Heart Institute and University of Missouri-Kansas City, Kansas City, MO, United States; **Matthew A. Cavender, MD MPH**, University of North Carolina, Chapel Hill, NC, United States; **Alex Z. Fu, PhD**, Georgetown University Medical Center, Washington DC, United States; **John P. Wilding MD, PhD**, University of Liverpool, Liverpool, United; Kingdom; **Kamlesh Khunti, MD PhD**, University of Leicester, Leicester, United Kingdom; **Anna Norhammar, MD**, Cardiology Unit, Department of Medicine, Karolinska Institutet, Stockhom, Sweden; **Kåre Birkeland, MD PhD**, Department of Endocrinology, University of Oslo, Norway; **Marit Eika Jørgensen, MD PhD**, cand.med, Steno Diabetes Center, Gentofte, Denmark; **Reinhard W. Holl MD PhD**, Institute of Epidemiology, University of Ulm, Ulm, Germany;

Executive Scientific Committee (AstraZeneca Members):

Niklas Hammar, PhD, Senior Director Epidemiology, AZ Gothenburg, Sweden and Institute of Environmental Medicine, Karolinska Institutet, Stockholm, Sweden (Study Principal Investigator); **Peter Fenici, MD, PhD**, Global Medical Affairs Senior Leader, AZ, Academy House, Cambridge, United Kingdom (Study Leader); **Niki Arya**, **MSc**, Principal Statistician, Biometrics and Information Sciences, AZ Gaithersburg, MD, United States (Study Statistician); **Kyle Nahrebne, MSc**, Global Publication Leader, AZ Gaithersburg, MD, United (Study Publication Leader);

All Members of the Executive Steering Committee have contributed to data interpretation and approved this manuscripts analyses and conclusions. Executive Scientific Committee Academic Members MK, MAC AZF, JPW, KK, AN have also reviewed and input the study protocol and amendment, together with the Executive Scientific Committee AZ Members NH, PF, NA, KN and with the Study Core Team Members, KAS, JB, BTB, SED, KB, MFS. Study meta analyses have been conducted by Niki Arya (AstraZeneca) and Marcus Thuresson (Statisticon).

***Study Core Team (AstraZeneca Members)*:**

Karolina Andersson-Sundell, MSc PhD, Assoc .Dir. Epidemiology, MEOR, AZ Gothenburg, Sweden; **Johan Bodegård, MD PhD**, Medical Evidence Scientific Lead Nordics/Baltic, AZ Oslo, Norway; **Betina T. Blak, MSc PhD** RWE Scientific Leader, AZ Luton, United Kingdom; **Sara E. Dempster, PhD** Associate Principal Informatics Scientist, AZ Waltham, MA, United States; **Kelly Bell PharmD, MSPhr,** HEOR Director, AZ Wilmington, DE, United States; **Eric T. Wittbrodt, PharmD**, **MPH**, HEOR Director, AZ Wilmington, DE, United States; **Markus F. Scheerer, MSc PhD**, Scientific Advisor Diabetes, AZ Wedel, Germany

All Study Core Team Members have contributed to country level analyses and local coordination with databases analysts in their respective country. Study Core Team Members, KAS, JB, BTB, SED, KB, MFS have also reviewed and input the study protocol design and amendments.

External Investigators and Analysts:

Hanne Løvdal Gulseth, MD, PhD, Department of Endocrinology, Oslo University Hospital, Aker, Norway; **Bendix Carstensen, PhD**, Diabetes Center, Gentofte, Denmark; **Marcus Thuresson, PhD** Statistician (Study Statistician), Statisticon AB, Uppsala, Sweden; **Esther Bollow**, Institute of Epidemiology, University of Ulm, Ulm, Germany; **Luis Alberto García Rodríguez, MD**, CEIFE - Centro Español de Investigación Farmacoepidemiológica, Madrid, Spain; **Lucia Cea Soriano, PharmD, PhD**, CEIFE – Centro Español de Investigación Farmacoepidemiológica and Department of Preventive Medicine and Public Health, Faculty of Medicine,
Complutense University of Madrid, Spain; **Oscar Fernándex Cantero**, CEIFE – Centro Español de Investigación Farmacoepidemiológica; **Ellen Riehle, MPH**, Senior Research Analyst, Truven Health Analytics, an IBM Company, Ann Arbor, MI; **Brian Murphy, MS**, Senior Data Analyst, Evidera, Waltham, MA.

All other External Investigators and Analysts Members have contributed to country level data analyses, quality check and validation and results interpretation in their respective country.

Characteristics of the databases

Truven Health MarketScan® Commercial Claims and Encounters (Commercial) and Medicare Supplemental and Coordination of Benefits (Medicare Supplemental) databases comprise enrolment information, demographic information and inpatient medical, outpatient medical and outpatient pharmacy claims data from over 300 large self-insured US employers and over 25 US health plans[1]. These study databases satisfy the conditions set forth in Sections 164.514 (a)-(b)1ii of the Health Insurance Portability and Accountability Act of 1996 privacy rule regarding the determination and documentation of statistically de-identified data Thus, the study did not require external IRB or ethics review. The Truven MarketScan database has been used for comparative effectiveness research since 1989, with over 1100 peer-reviewed publications overall and is considered to be nationally representative in the US for the included patient populations.[2] Truven MarketScan is highly representative of the US commercially insured population aged <65 years, without contributing any specific bias to create differences from the commercially insured population as a whole. Comparisons of age, gender, and region for Truven MarketScan with US Census data (2010) demonstrate close similarities between the two datasets (data on file).

Clinical Practice Research Datalink (CPRD) holds anonymized longitudinal primary care patient records collected from over 670 general practices across the UK, covering >11.3 million patients. It includes diagnoses, issued drug prescriptions, clinical measures taken within the general practice, lab tests and referrals to specialist care.[3] Hospitalisation information and specialist care notes are generally recorded by the general practitioner into the primary care patient records. Of the UK practices included in CPRD, 58% are linked to the Hospital Episode Statistics (HES) dataset[3] with detailed hospitalisation information (excluding drug use) on all hospitalisation episodes in England, and to death certificates from the Office of National Statistics (ONS) to derive estimates of all-cause and cause-specific death. CPRD includes approximately 6.9% of the UK population, and patients are broadly representative of the UK general population.[3] Herrett et al [3] assesses the representativeness of CPRD by comparing the age and sex distribution within CPRD to the UK Census in 2011. They found that CPRD was broadly representative of the UK population with respect to age and sex. In addition, Herrett et al 2010 [4] published a literature review assessing the validation and validity of diagnoses in CPRD (formerly known as GPRD). They concluded that overall, estimates of validity were high. A detailed description of CPRD can be found in a paper by Williams et al.[5] This generalizability of CPRD to the UK population has resulted in CPRD being used in over 1000 publications.[3]

The Health Improvement Network (THIN) includes data from >580 UK practices, with similar data to CPRD.[6] THIN coverage of the UK population by 2013 was 5.67%[7] and the representativeness of THIN was described by Blak et al by comparing the distribution of deprivation, morbidities (Quality and Outcomes Framework [QOF] conditions) and demographics to national statistics and national QOF 2006/2007 data.[8] They demonstrated that THIN is representative of the UK population with respect to morbidities, demographics, and mortality rates. Furthermore, in THIN, it was found that between 1990 and 2009 the standardized mortality ratio ranged from 0.81 (95% CI: 0.39–1.49; 1990) to 0.93 (95% CI: 0.48–1.64; 1995). Adjusting for demographics/deprivation, the

2006 THIN death rate was 9.08/1000 population, which is consistent with the national death rate of 9.4/1000 population. When Blak et al. was originally published, THIN included 532 general practices. Currently there are approximately 600 general practices. A description of the development of THIN is described in Bourke et al.[9]

Lewis et al validated THIN by comparing drug-disease associations found in the original GPRD practices included in THIN, to drug-disease associations found in the new practices included in THIN.[10] They concluded that: '*THIN data that are collected outside of the GPRD appear as valid as the data collected as part of the GPRD*'. Other validation studies of THIN are also available,[11, 12] and similar to CPRD, THIN has been used for numerous publications (~400 to date; http://www.epic-uk.org/our-data/statistics.shtml.), some of which compare THIN data results to external measures, demonstrating similarities.[13-18]

Some of the general practices providing data to CPRD are also providing data to THIN. Therefore, there is an overlap and a potential to combine the two databases in order to increase patient numbers. A recent study within diabetes has shown that 61% of CPRD patients are also included in THIN.[6] This overlap in patients has been taken into account in the present study so that no individual is double counted. The duplicated practices list between CPRD and THIN was obtained by looking for coincidences of patients by practices between both databases. The parameters used for the match were sex, year of birth, family id, and date and Read code of diabetes.[7, 19] For the analyses all THIN practices were retained and the non-overlapping CPRD HES and ONS linked practices. In CPRD, HHF was identified in linked HES with International Statistical Classification of Diseases and Related Health Problems (ICD-system) 10 codes I50.x and all-cause death was identified in linked ONS death data. HHF and all-cause death was identified in the general practice medical records in THIN. For HFF, free text from THIN was collected within +-365 days of event and reviewed to ascertain and validate the cause of hospitalisation and the event date. The reviewers were blinded to the index medication. Patients were selected from THIN and/or CPRD from November 2012 to September 2015 (THIN) and to January 2016 (CPRD). This study was approved by the Scientific Review Committee (SRC) of THIN; protocol approval number: 16THIN027A1. Further, the Independent Scientific Advisory Committee (ISAC) of CPRD approved it; protocol approval number: 16_064RAR.

The Diabetes-Patienten-Verlaufsdokumentation (DPV; or "Diabetes Prospective Follow-up") initiative involves more than 400 clinical centres predominantly from Germany and Austria, documenting data pertaining to diabetes. Similar to an electronic medical record, relevant data are documented only once and are available for numerous purposes: graphical and tabular description, medical report, treatment plan, diabetes passport, reminder on upcoming visits (watchdog), certification of centers and type 2 diabetes program (DMP) documentation. Every 6 months, anonymized data are sent to the University of Ulm, Germany.[20] Analyses are conducted separately for pediatric and adult patients either anonymised or centre-based after prior written informed consent for regional quality circles. Data of the anonymised DPV registry are used for treatment research in order to investigate practice-oriented questions. A publication list can be found on the website under 'publications' (www.d-p-v.eu). Data from this registry have been used extensively in multi-center outcomes

research, with 452 centers participating. The DPV-initiative is the multicenter benchmark database for patients with diabetes in Germany, includes all levels of diabetes care for the German population of patients with T2D, and has been used in over 370 peer-reviewed publications.[20] The DPV Type 2 diabetes population is consistent with that observed in the national German Health Interview and Examination Surveys [21] when considering the inclusion and exclusion criteria of the CVD-REAL study. In addition, demographic characteristics of the DPV population are consistent with other large data collections of patients with diabetes from Germany, such as the DMP data from Northrhine (https://www.kvno.de/downloads/quali/qualbe_dmp14.pdf) or the DIVE registry (http://www.dive-register.de/).

The National Prescribed Drug Registers in Sweden, Norway and Denmark have full coverage of each country's population. Patients were followed with regard to outcome in the National Patient Register and National Cause of Death Register. The Swedish national database[22] includes information from linkage of three national Swedish registries held by the Swedish National Board of Health and Welfare, with full coverage of the Swedish population: 1) The Prescribed Drug Register July 1, 2005 to December 2016, covering all drug prescriptions filled using Anatomical Therapeutic Chemical codes; 2) The Cause of Death Register 1961–2015; 3) The National Patient Register covering all hospital admissions and discharge diagnoses in 1987–2015, discharge diagnoses, specialized care and open patient clinic visits in 2001–2015. Diagnoses are recorded according to the International Statistical Classification of Diseases and Related Health Problems (ICD) system. Similarly, the Norwegian national database[23] includes type 2 diabetes patient information from three national Norwegian registries with full coverage of the Norwegian population: the Norwegian Prescription Database (July 2004 to July 2016) covering all filled drug prescriptions using ATC codes; the Norwegian Cause of Death Register (1958 to 2014); and the Norwegian Patient Register covering all hospital out-patient clinic visit and discharge diagnoses and all hospital discharge diagnoses for the years 2008 to July 2016. Diagnoses are recorded according to the ICD-system. Data linkage is performed by the Norwegian Institute of Public Health. The Danish national database[24] also has a similar structure, and includes T2D patient information from three national Danish registries with full coverage of the Danish population: the Prescribed Drug Register (1990 to 2015) covering all filled drug prescriptions using ATC codes; the Cause of Death Register (1952– to 2014); and the National Patient Register covering all open patient clinic visit diagnoses for 2000 to 2015 and all hospital discharge diagnoses for the years 1980 to 2015, and discharge diagnoses and. Diagnoses are recorded according to the ICD-system. All three registers are held by the Statistics Denmark. Data from Statistics Denmark were made available following an application to Statistics Denmark. The Danish study was approved by the Danish Data Protection Agency (Datatilsynet, registration number 2015-41-4148).

Data were anonymised, and the requirement for informed consent was therefore waived according to standard analytical procedures with each database owner.

Statistical power calculations

For the primary outcome (HHF) a risk reduction of 20% for SGLT-2i versus oGLD was considered clinically meaningful. For 85% power to detect a risk reduction of 20% with a two-sided α-level of 0.05 and a 1:1 treatment allocation (SGLT-2i vs. oGLD), a total of 730 events across the matched treatment groups in all the datasets was required. As there were a total of 961 HHF events within the matched cohorts, we had sufficient power to perform the HHF analysis.

Analyses were conducted using R- version 3.2.3[25, 26] in Sweden, Norway and Denmark, STATA version 12.0 (StataCorp LP, College Station, TX, US) in the UK, STATA version 12.0 (StataCorp LP, College Station, TX, US) and SAS version 9.4 (Cary, NC) in the US, and SAS version 9.4 (TS1M1) (Cary, NC, US) in Germany.

SUPPLEMENTARY TABLES

Table S1. ICD and Read codes for Type 2 diabetes, Type 1 diabetes and gestational diabetes

**Type 2 diabetes**	
ICD codes	
ICD-9 250.X0, 250.X2	
ICD-10 codes E11 and 024.1	
Read codes	
66A4.00	Diabetic on oral treatment
66Ao.00	Diabetes type 2 review
66At100	Type II diabetic dietary review
66At111	Type 2 diabetic dietary review
66AV.00	Diabetic on insulin and oral treatment
C100100	Diabetes mellitus, adult onset, no mention of complication
C100111	Maturity onset diabetes
C100112	Non-insulin dependent diabetes mellitus
C101100	Diabetes mellitus, adult onset, with ketoacidosis
C102100	Diabetes mellitus, adult onset, with hyperosmolar coma
C103100	Diabetes mellitus, adult onset, with ketoacidotic coma
C104100	Diabetes mellitus, adult onset, with renal manifestation
C105100	Diabetes mellitus, adult onset, + ophthalmic manifestation
C106100	Diabetes mellitus, adult onset, + neurological manifestation
C107100	Diabetes mellitus, adult, + peripheral circulatory disorder
C107200	Diabetes mellitus, adult with gangrene
C107400	NIDDM with peripheral circulatory disorder
C109.00	Non-insulin dependent diabetes mellitus
C109.11	NIDDM - Non-insulin dependent diabetes mellitus

C109.12	Type 2 diabetes mellitus
C109.13	Type II diabetes mellitus
C109000	Non-insulin-dependent diabetes mellitus with renal comps
C109011	Type II diabetes mellitus with renal complications
C109012	Type 2 diabetes mellitus with renal complications
C109100	Non-insulin-dependent diabetes mellitus with ophthalm comps
C109111	Type II diabetes mellitus with ophthalmic complications
C109112	Type 2 diabetes mellitus with ophthalmic complications
C109200	Non-insulin-dependent diabetes mellitus with neuro comps
C109211	Type II diabetes mellitus with neurological complications
C109212	Type 2 diabetes mellitus with neurological complications
C109300	Non-insulin-dependent diabetes mellitus with multiple comps
C109400	Non-insulin dependent diabetes mellitus with ulcer
C109411	Type II diabetes mellitus with ulcer
C109412	Type 2 diabetes mellitus with ulcer
C109500	Non-insulin dependent diabetes mellitus with gangrene
C109511	Type II diabetes mellitus with gangrene
C109512	Type 2 diabetes mellitus with gangrene
C109600	Non-insulin-dependent diabetes mellitus with retinopathy
C109611	Type II diabetes mellitus with retinopathy
C109612	Type 2 diabetes mellitus with retinopathy
C109700	Non-insulin dependent diabetes mellitus - poor control
C109711	Type II diabetes mellitus - poor control
C109712	Type 2 diabetes mellitus - poor control
C109900	Non-insulin-dependent diabetes mellitus without complication
C109A00	Non-insulin dependent diabetes mellitus with mononeuropathy
C109A11	Type II diabetes mellitus with mononeuropathy

C109B00	Non-insulin dependent diabetes mellitus with polyneuropathy
C109B11	Type II diabetes mellitus with polyneuropathy
C109C00	Non-insulin dependent diabetes mellitus with nephropathy
C109C11	Type II diabetes mellitus with nephropathy
C109C12	Type 2 diabetes mellitus with nephropathy
C109D00	Non-insulin dependent diabetes mellitus with hypoglyca coma
C109D11	Type II diabetes mellitus with hypoglycaemic coma
C109D12	Type 2 diabetes mellitus with hypoglycaemic coma
C109E00	Non-insulin depend diabetes mellitus with diabetic cataract
C109E11	Type II diabetes mellitus with diabetic cataract
C109E12	Type 2 diabetes mellitus with diabetic cataract
C109F00	Non-insulin-dependent d m with peripheral angiopath
C109F11	Type II diabetes mellitus with peripheral angiopathy
C109F12	Type 2 diabetes mellitus with peripheral angiopathy
C109G00	Non-insulin dependent diabetes mellitus with arthropathy
C109G11	Type II diabetes mellitus with arthropathy
C109G12	Type 2 diabetes mellitus with arthropathy
C109H00	Non-insulin dependent d m with neuropathic arthropathy
C109H11	Type II diabetes mellitus with neuropathic arthropathy
C109H12	Type 2 diabetes mellitus with neuropathic arthropathy
C109J00	Insulin treated Type 2 diabetes mellitus
C109J11	Insulin treated non-insulin dependent diabetes mellitus
C109J12	Insulin treated Type II diabetes mellitus
C109K00	Hyperosmolar non-ketotic state in type 2 diabetes mellitus
C10C.11	Maturity onset diabetes in youth
C10D.00	Diabetes mellitus autosomal dominant type 2
C10D.11	Maturity onset diabetes in youth type 2

C10F.00	Type 2 diabetes mellitus
C10F.11	Type II diabetes mellitus
C10F000	Type 2 diabetes mellitus with renal complications
C10F011	Type II diabetes mellitus with renal complications
C10F100	Type 2 diabetes mellitus with ophthalmic complications
C10F111	Type II diabetes mellitus with ophthalmic complications
C10F200	Type 2 diabetes mellitus with neurological complications
C10F211	Type II diabetes mellitus with neurological complications
C10F300	Type 2 diabetes mellitus with multiple complications
C10F311	Type II diabetes mellitus with multiple complications
C10F400	Type 2 diabetes mellitus with ulcer
C10F411	Type II diabetes mellitus with ulcer
C10F500	Type 2 diabetes mellitus with gangrene
C10F511	Type II diabetes mellitus with gangrene
C10F600	Type 2 diabetes mellitus with retinopathy
C10F611	Type II diabetes mellitus with retinopathy
C10F700	Type 2 diabetes mellitus - poor control
C10F711	Type II diabetes mellitus - poor control
C10F900	Type 2 diabetes mellitus without complication
C10F911	Type II diabetes mellitus without complication
C10FA00	Type 2 diabetes mellitus with mononeuropathy
C10FA11	Type II diabetes mellitus with mononeuropathy
C10FB00	Type 2 diabetes mellitus with polyneuropathy
C10FB11	Type II diabetes mellitus with polyneuropathy
C10FC00	Type 2 diabetes mellitus with nephropathy
C10FC11	Type II diabetes mellitus with nephropathy
C10FD00	Type 2 diabetes mellitus with hypoglycaemic coma

C10FD11	Type II diabetes mellitus with hypoglycaemic coma
C10FE00	Type 2 diabetes mellitus with diabetic cataract
C10FE11	Type II diabetes mellitus with diabetic cataract
C10FF00	Type 2 diabetes mellitus with peripheral angiopathy
C10FF11	Type II diabetes mellitus with peripheral angiopathy
C10FG00	Type 2 diabetes mellitus with arthropathy
C10FG11	Type II diabetes mellitus with arthropathy
C10FH00	Type 2 diabetes mellitus with neuropathic arthropathy
C10FJ00	Insulin treated Type 2 diabetes mellitus
C10FJ11	Insulin treated Type II diabetes mellitus
C10FK00	Hyperosmolar non-ketotic state in type 2 diabetes mellitus
C10FL00	Type 2 diabetes mellitus with persistent proteinuria
C10FL11	Type II diabetes mellitus with persistent proteinuria
C10FM00	Type 2 diabetes mellitus with persistent microalbuminuria
C10FM11	Type II diabetes mellitus with persistent microalbuminuria
C10FN00	Type 2 diabetes mellitus with ketoacidosis
C10FP00	Type 2 diabetes mellitus with ketoacidotic coma
C10FQ00	Type 2 diabetes mellitus with exudative maculopathy
C10FR00	Type 2 diabetes mellitus with gastroparesis
C10K.00	Type A insulin resistance
C10K000	Type A insulin resistance without complication
C10z100	Diabetes mellitus, adult onset, + unspecified complication
L180600	Pre-existing diabetes mellitus, non-insulin-dependent
**Type 1 diabetes**	
ICD codes	
ICD-9 codes 250.x1, 250.X3	
ICD-10 codes E10 and O24	

Read codes	
66An.00	Diabetes type 1 review
C108D11	Type I diabetes mellitus with nephropathy
66An.00	Diabetes type 1 review
66At000	Type I diabetic dietary review
66At011	Type 1 diabetic dietary review
C100011	Insulin dependent diabetes mellitus
C101000	Diabetes mellitus, juvenile type, with ketoacidosis
C102000	Diabetes mellitus, juvenile type, with hyperosmolar coma
C103000	Diabetes mellitus, juvenile type, with ketoacidotic coma
C104000	Diabetes mellitus, juvenile type, with renal manifestation
C105000	Diabetes mellitus, juvenile type, + ophthalmic manifestation
C106000	Diabetes mellitus, juvenile, + neurological manifestation
C107000	Diabetes mellitus, juvenile +peripheral circulatory disorder
C107300	IDDM with peripheral circulatory disorder
C108.00	Insulin dependent diabetes mellitus
C108.11	IDDM-Insulin dependent diabetes mellitus
C108.12	Type 1 diabetes mellitus
C108.13	Type I diabetes mellitus
C108000	Insulin-dependent diabetes mellitus with renal complications
C108011	Type I diabetes mellitus with renal complications
C108012	Type 1 diabetes mellitus with renal complications
C108100	Insulin-dependent diabetes mellitus with ophthalmic comps
C108112	Type 1 diabetes mellitus with ophthalmic complications
C108200	Insulin-dependent diabetes mellitus with neurological comps
C108211	Type I diabetes mellitus with neurological complications
C108212	Type 1 diabetes mellitus with neurological complications

C108300	Insulin dependent diabetes mellitus with multiple complicatn
C108400	Unstable insulin dependent diabetes mellitus
C108411	Unstable type I diabetes mellitus
C108412	Unstable type 1 diabetes mellitus
C108500	Insulin dependent diabetes mellitus with ulcer
C108511	Type I diabetes mellitus with ulcer
C108512	Type 1 diabetes mellitus with ulcer
C108600	Insulin dependent diabetes mellitus with gangrene
C108700	Insulin dependent diabetes mellitus with retinopathy
C108711	Type I diabetes mellitus with retinopathy
C108712	Type 1 diabetes mellitus with retinopathy
C108800	Insulin dependent diabetes mellitus - poor control
C108811	Type I diabetes mellitus - poor control
C108812	Type 1 diabetes mellitus - poor control
C108900	Insulin dependent diabetes maturity onset
C108911	Type I diabetes mellitus maturity onset
C108912	Type 1 diabetes mellitus maturity onset
C108A00	Insulin-dependent diabetes without complication
C108A11	Type I diabetes mellitus without complication
C108B00	Insulin dependent diabetes mellitus with mononeuropathy
C108B11	Type I diabetes mellitus with mononeuropathy
C108C00	Insulin dependent diabetes mellitus with polyneuropathy
C108D00	Insulin dependent diabetes mellitus with nephropathy
C108D11	Type I diabetes mellitus with nephropathy
C108E00	Insulin dependent diabetes mellitus with hypoglycaemic coma
C108E11	Type I diabetes mellitus with hypoglycaemic coma
C108E12	Type 1 diabetes mellitus with hypoglycaemic coma

C108F00	Insulin dependent diabetes mellitus with diabetic cataract
C108F11	Type I diabetes mellitus with diabetic cataract
C108G00	Insulin dependent diab mell with peripheral angiopathy
C108H00	Insulin dependent diabetes mellitus with arthropathy
C108H11	Type I diabetes mellitus with arthropathy
C108J00	Insulin dependent diab mell with neuropathic arthropathy
C108J11	Type I diabetes mellitus with neuropathic arthropathy
C108J12	Type 1 diabetes mellitus with neuropathic arthropathy
C10C.12	Maturity onset diabetes in youth type 1
C10E.00	Type 1 diabetes mellitus
C10E.11	Type I diabetes mellitus
C10E.12	Insulin dependent diabetes mellitus
C10E000	Type 1 diabetes mellitus with renal complications
C10E012	Insulin-dependent diabetes mellitus with renal complications
C10E100	Type 1 diabetes mellitus with ophthalmic complications
C10E111	Type I diabetes mellitus with ophthalmic complications
C10E112	Insulin-dependent diabetes mellitus with ophthalmic comps
C10E200	Type 1 diabetes mellitus with neurological complications
C10E212	Insulin-dependent diabetes mellitus with neurological comps
C10E300	Type 1 diabetes mellitus with multiple complications
C10E311	Type I diabetes mellitus with multiple complications
C10E312	Insulin dependent diabetes mellitus with multiple complicat
C10E400	Unstable type 1 diabetes mellitus
C10E411	Unstable type I diabetes mellitus
C10E412	Unstable insulin dependent diabetes mellitus
C10E500	Type 1 diabetes mellitus with ulcer
C10E511	Type I diabetes mellitus with ulcer

C10E512	Insulin dependent diabetes mellitus with ulcer
C10E600	Type 1 diabetes mellitus with gangrene
C10E611	Type I diabetes mellitus with gangrene
C10E700	Type 1 diabetes mellitus with retinopathy
C10E711	Type I diabetes mellitus with retinopathy
C10E712	Insulin dependent diabetes mellitus with retinopathy
C10E800	Type 1 diabetes mellitus - poor control
C10E812	Insulin dependent diabetes mellitus - poor control
C10E900	Type 1 diabetes mellitus maturity onset
C10E911	Type I diabetes mellitus maturity onset
C10E912	Insulin dependent diabetes maturity onset
C10EA00	Type 1 diabetes mellitus without complication
C10EA11	Type I diabetes mellitus without complication
C10EA12	Insulin-dependent diabetes without complication
C10EB00	Type 1 diabetes mellitus with mononeuropathy
C10EC00	Type 1 diabetes mellitus with polyneuropathy
C10EC11	Type I diabetes mellitus with polyneuropathy
C10EC12	Insulin dependent diabetes mellitus with polyneuropathy
C10ED00	Type 1 diabetes mellitus with nephropathy
C10ED12	Insulin dependent diabetes mellitus with nephropathy
C10EE00	Type 1 diabetes mellitus with hypoglycaemic coma
C10EE12	Insulin dependent diabetes mellitus with hypoglycaemic coma
C10EF00	Type 1 diabetes mellitus with diabetic cataract
C10EF12	Insulin dependent diabetes mellitus with diabetic cataract
C10EG00	Type 1 diabetes mellitus with peripheral angiopathy
C10EH00	Type 1 diabetes mellitus with arthropathy
C10EJ00	Type 1 diabetes mellitus with neuropathic arthropathy

C10EK00	Type 1 diabetes mellitus with persistent proteinuria
C10EL00	Type 1 diabetes mellitus with persistent microalbuminuria
C10EL11	Type I diabetes mellitus with persistent microalbuminuria
C10EM00	Type 1 diabetes mellitus with ketoacidosis
C10EM11	Type I diabetes mellitus with ketoacidosis
C10EN00	Type 1 diabetes mellitus with ketoacidotic coma
C10EN11	Type I diabetes mellitus with ketoacidotic coma
C10EP00	Type 1 diabetes mellitus with exudative maculopathy
C10EP11	Type I diabetes mellitus with exudative maculopathy
C10EQ00	Type 1 diabetes mellitus with gastroparesis
C10z000	Diabetes mellitus, juvenile type, + unspecified complication
L180500	Pre-existing diabetes mellitus, insulin-dependent
8Hj3.00	Referral to DAFNE diabetes structured education programme
8Hj4.00	Referral to DESMOND diabetes structured education programme
8Hj5.00	Referral to XPERT diabetes structured education programme
8I82.00	Did not complete DAFNE diabetes structured education program
8I83.00	Did not complete DESMOND diabetes structured educat program
8I84.00	Did not complete XPERT diabetes structured education program
9NiC.00	Did not attend DAFNE diabetes structured education programme
9NiD.00	Did not attend DESMOND diabetes structured education program
9NiE.00	Did not attend XPERT diabetes structured education programme
9OLG.00	Attended XPERT diabetes structured education programme
9OLH.00	Attended DAFNE diabetes structured education programme
9OLJ.00	DAFNE diabetes structured education programme completed
9OLK.00	DESMOND diabetes structured education programme completed
9OLL.00	XPERT diabetes structured education programme completed
**Gestational Diabetes**	

ICD codes	
ICD-9 codes 648.8	
ICD-10:O24.4	
Read codes	
ZC2CB00	Dietary advice for gestational diabetes
ZV13F00	[V]Personal history of gestational diabetes mellitus
L180900	Gestational diabetes mellitus
L180811	Gestational diabetes mellitus
8CE0000	Gestational diabetes information leaflet given
66Ay.00	Gestational diabetes mellitus annual review
8CE0000	Gestational diabetes information leaflet given
Q44B.00	Syndrome of infant of mother with gestational diabetes
66AX.00	Diabetes: shared care in pregnancy - diabetol and obstet
6761.00	Diabetic pre-pregnancy counselling
L180.00	Diabetes mellitus during pregnancy/childbirth/puerperium
L180000	Diabetes mellitus - unspec whether in pregnancy/puerperium
L180100	Diabetes mellitus during pregnancy - baby delivered
L180300	Diabetes mellitus during pregnancy - baby not yet delivered
L180800	Diabetes mellitus arising in pregnancy
L180z00	Diabetes mellitus in pregnancy/childbirth/puerperium NOS

Table S2. Read codes for history of cardiovascular events

**Myocardial infarction**	
G30..00	Acute myocardial infarction
G30..13	Cardiac rupture following myocardial infarction (MI)
G300.00	Acute anterolateral infarction
G301.00	Other specified anterior myocardial infarction
G301000	Acute anteroapical infarction
G30..11	Attack - heart
G30..14	Heart attack
G30..15	MI - acute myocardial infarction
G301100	Acute anteroseptal infarction
G301z00	Anterior myocardial infarction NOS
G302.00	Acute inferolateral infarction
G303.00	Acute inferoposterior infarction
G304.00	Posterior myocardial infarction NOS
G305.00	Lateral myocardial infarction NOS
G306.00	True posterior myocardial infarction
G307.00	Acute subendocardial infarction
G307000	Acute non-Q wave infarction
G308.00	Inferior myocardial infarction NOS
G309.00	Acute Q-wave infarct
G30B.00	Acute posterolateral myocardial infarction
G30X.00	Acute transmural myocardial infarction of unspecif site
G30X000	Acute ST segment elevation myocardial infarction
G30y.00	Other acute myocardial infarction
G30y000	Acute atrial infarction
G30y100	Acute papillary muscle infarction
G30y200	Acute septal infarction
G30yz00	Other acute myocardial infarction NOS
G30z.00	Acute myocardial infarction NOS
G31..00	Other acute and subacute ischaemic heart disease
G311500	Acute coronary syndrome
G310.00	Postmyocardial infarction syndrome

G31y100	Microinfarction of heart
G31y200	Subendocardial ischaemia
G35..00	Subsequent myocardial infarction
G350.00	Subsequent myocardial infarction of anterior wall
G351.00	Subsequent myocardial infarction of inferior wall
G353.00	Subsequent myocardial infarction of other sites
G35X.00	Subsequent myocardial infarction of unspecified site
3232.00	ECG: old myocardial infarction
3235.00	ECG: subendocardial infarct
323Z.00	ECG: myocardial infarct NOS
889A.00	Diab mellit insulin-glucose infus acute myocardial infarct
G307100	Acute non-ST segment elevation myocardial infarction
G312.00	Coronary thrombosis not resulting in myocardial infarction
G36..00	Certain current complication follow acute myocardial infarct
G360.00	Haemopericardium/current comp folow acut myocard infarct
G364.00	Ruptur chordae tendinae/curr comp fol acute myocard infarct
G365.00	Rupture papillary muscle/curr comp fol acute myocard infarct
G384.00	Postoperative subendocardial myocardial infarction
Gyu3100	[X]Other current complicatns following acute myocard infarct
Gyu3400	[X]Acute transmural myocardial infarction of unspecif site
**Unstable Angina**	
G311.11	Crescendo angina
G311.13	Unstable angina
G311.14	Angina at rest
G311100	Unstable angina
G311200	Angina at rest
G311300	Refractory angina
G311400	Worsening angina
G330.00	Angina decubitus
G330000	Nocturnal angina
G330z00	Angina decubitus NOS
G331.00	Prinzmetal's angina
G331.11	Variant angina pectoris

**Ischemic Stroke**	
14A7.00	H/O: CVA/stroke
14A7.11	H/O: CVA
14A7.12	H/O: stroke
14AK.00	H/O: Stroke in last year
662M100	Stroke 6 month review
662M200	Stroke initial post discharge review
8HBJ.00	Stroke / transient ischaemic attack referral
G63..11	Infarction - precerebral
G63y000	Cerebral infarct due to thrombosis of precerebral arteries
G63y100	Cerebral infarction due to embolism of precerebral arteries
G64..11	CVA - cerebral artery occlusion
G64..12	Infarction - cerebral
G64..13	Stroke due to cerebral arterial occlusion
G640.00	Cerebral thrombosis
G640000	Cerebral infarction due to thrombosis of cerebral arteries
G641.00	Cerebral embolism
G641.11	Cerebral embolus
G641000	Cerebral infarction due to embolism of cerebral arteries
G64z.00	Cerebral infarction NOS
G64z.11	Brainstem infarction NOS
G64z.12	Cerebellar infarction
G64z000	Brainstem infarction
G64z100	Wallenberg syndrome
G64z111	Lateral medullary syndrome
G64z200	Left sided cerebral infarction
G64z300	Right sided cerebral infarction
G64z400	Infarction of basal ganglia
G654.00	Multiple and bilateral precerebral artery syndromes
G66..00	Stroke and cerebrovascular accident unspecified
G66..11	CVA unspecified
G66..12	Stroke unspecified
G66..13	CVA - Cerebrovascular accident unspecified

G660.00	Middle cerebral artery syndrome
G661.00	Anterior cerebral artery syndrome
G662.00	Posterior cerebral artery syndrome
G663.00	Brain stem stroke syndrome
G664.00	Cerebellar stroke syndrome
G665.00	Pure motor lacunar syndrome
G666.00	Pure sensory lacunar syndrome
G667.00	Left sided CVA
G668.00	Right sided CVA
G675.00	Moyamoya disease
G676.00	Nonpyogenic venous sinus thrombosis
G676000	Cereb infarct due cerebral venous thrombosis, nonpyogenic
G678.00	Cereb autosom dominant arteriop subcort infarcts leukoenceph
G683.00	Sequelae of cerebral infarction
G68X.00	Sequelae of stroke,not specfd as h'morrhage or infarction
G6W..00	Cereb infarct due unsp occlus/stenos precerebr arteries
G6X..00	Cerebrl infarctn due/unspcf occlusn or sten/cerebrl artrs
Gyu6300	[X]Cerebrl infarctn due/unspcf occlusn or sten/cerebrl artrs
Gyu6400	[X]Other cerebral infarction
Gyu6C00	[X]Sequelae of stroke,not specfd as h'morrhage or infarction
Gyu6G00	[X]Cereb infarct due unsp occlus/stenos precerebr arteries
L440.12	Stroke in the puerperium
ZLEP.00	Discharge from stroke serv
ZV12511	[V]Personal history of stroke
ZV12512	[V]Personal history of cerebrovascular accident (CVA)
**Hemostroke**	
7004100	Evacuation of haematoma from temporal lobe of brain
7004200	Evacuation of haematoma from cerebellum
7004300	Evacuation of intracerebral haematoma NEC
7008200	Aspiration of haematoma of brain tissue
G61..00	Intracerebral haemorrhage
G61..11	CVA - cerebrovascular accid due to intracerebral haemorrhage
G61..12	Stroke due to intracerebral haemorrhage

G610.00	Cortical haemorrhage
G611.00	Internal capsule haemorrhage
G612.00	Basal nucleus haemorrhage
G613.00	Cerebellar haemorrhage
G614.00	Pontine haemorrhage
G615.00	Bulbar haemorrhage
G616.00	External capsule haemorrhage
G617.00	Intracerebral haemorrhage, intraventricular
G618.00	Intracerebral haemorrhage, multiple localized
G619.00	Lobar cerebral haemorrhage
G61X.00	Intracerebral haemorrhage in hemisphere, unspecified
G61X000	Left sided intracerebral haemorrhage, unspecified
G61X100	Right sided intracerebral haemorrhage, unspecified
G61z.00	Intracerebral haemorrhage NOS
G681.00	Sequelae of intracerebral haemorrhage
Gyu6200	[X]Other intracerebral haemorrhage
Gyu6F00	[X]Intracerebral haemorrhage in hemisphere, unspecified
7017000	Evacuation of subdural haematoma
7034.00	Drainage of subdural space
7034y00	Other specified drainage of subdural space
7034z00	Drainage of subdural space NOS
G621.00	Subdural haemorrhage - nontraumatic
G622.00	Subdural haematoma - nontraumatic
G623.00	Subdural haemorrhage NOS
S62..13	Subdural haemorrhage following injury
S622.00	Closed traumatic subdural haemorrhage
S622000	Subdural h'ge inj no open intracranial wnd + unspec consc
S622100	Subdural h'ge inj no open intracranial wound+no loss consc
S622200	Subdural h'ge inj no open intracranial wound+<1hr loss consc
S622300	Subdural h'ge inj no open intracran wnd+1-24hr loss consc
S622400	Subdural h'ge inj no open intracranial wnd+>24 LOC +recovery
S622500	Subdural h'ge inj no open intracran wnd+>24hr LOC -restored
S622600	Subdural h'ge inj no open intracran wnd+LOC unspec duration

S622z00	Subdural h'ge inj no open intracran wound+concussion unspec
S623.00	Open traumatic subdural haemorrhage
S623000	Subdural h'ge inj + open intracranial wound + unspec consc
S623100	Subdural h'ge inj + open intracranial wound+no loss consc
S623200	Subdural h'ge inj + open intracranial wound+<1hr loss consc
S623300	Subdural h'ge inj + open intracranial wnd+1-24hr loss consc
S623400	Subdural h'ge inj + open intracran wound+>24hr LOC +recovery
S623500	Subdural h'ge inj + open intracran wnd+>24hr LOC -restored
S623600	Subdural h'ge inj + open intracran wnd+LOC unspec duration
S623z00	Subdural h'ge inj + open intracranial wnd+concussion unspec
S628.00	Traumatic subdural haemorrhage
S629.00	Traumatic subdural haematoma
S629000	Traumatic subdural haematoma without open intracranial wound
S629100	Traumatic subdural haematoma with open intracranial wound
G60..00	Subarachnoid haemorrhage
G601.00	Subarachnoid haemorrhage from carotid siphon and bifurcation
G602.00	Subarachnoid haemorrhage from middle cerebral artery
G603.00	Subarachnoid haemorrhage from anterior communicating artery
G604.00	Subarachnoid haemorrhage from posterior communicating artery
G605.00	Subarachnoid haemorrhage from basilar artery
G606.00	Subarachnoid haemorrhage from vertebral artery
G60X.00	Subarachnoid haemorrh from intracranial artery, unspecif
G60z.00	Subarachnoid haemorrhage NOS
G680.00	Sequelae of subarachnoid haemorrhage
Gyu6000	[X]Subarachnoid haemorrhage from other intracranial arteries
Gyu6100	[X]Other subarachnoid haemorrhage
Gyu6E00	[X]Subarachnoid haemorrh from intracranial artery, unspecif
S62..12	Subarachnoid haemorrhage following injury
S620.00	Closed traumatic subarachnoid haemorrhage
S620000	Subarachnoid h'ge inj no open intracran wound + unspec consc
S620100	Subarachnoid h'ge inj no open intracran wnd+no loss consc
S620200	Subarachnoid h'ge inj no open intracran wnd+<1hr loss consc
S620300	Subarachnoid h'ge inj no open intracran wound + 1-24hr LOC

S620400	Subarachnoid h'ge inj no open intracran wnd+>24 LOC+recovery
S620500	Subarach h'ge inj no open intracran wnd+>24hrs LOC-restored
S620600	Subarach h'ge inj no open intracran wnd+LOC unspec duration
S620z00	Subarach h'ge inj no open intracran wnd + concussion unspec
S621.00	Open traumatic subarachnoid haemorrhage
S621000	Subarachnoid h'ge inj + open intracran wound + unspec consc
S621100	Subarachnoid h'ge inj + open intracranial wound + no LOC
S621200	Subarachnoid h'ge inj + open intracran wound+<1hr loss consc
S621300	Subarachnoid h'ge inj + open intracran wnd+1-24hr loss consc
S621400	Subarach h'ge inj + open intracran wnd +>24hr LOC + recovery
S621500	Subarach h'ge inj + open intracran wnd+>24hr LOC -restored
S621600	Subarach h'ge inj + open intracran wnd+LOC unspec duration
S621z00	Subarachnoid h'ge inj + open intracran wnd+concussion unspec
S627.00	Traumatic subarachnoid haemorrhage
7032000	Evacuation of extradural haematoma
G600.00	Ruptured berry aneurysm
G62..00	Other and unspecified intracranial haemorrhage
G620.00	Extradural haemorrhage - nontraumatic
G62z.00	Intracranial haemorrhage NOS
G682.00	Sequelae of other nontraumatic intracranial haemorrhage
Gyu6B00	[X]Sequelae of other nontraumatic intracranial haemorrhage
S62..00	Cerebral haemorrhage following injury
S62..11	Extradural haemorrhage following injury
S62..14	Traumatic cerebral haemorrhage
S620.11	Middle meningeal haemmorhage following injury
S624.00	Closed traumatic extradural haemorrhage
S624.11	Epidural haematoma following injury
S624000	Extradural h'ge inj no open intracranial wnd + unspec consc
S624100	Extradural h'ge inj no open intracranial wnd + no loss consc
S624200	Extradural h'ge inj no open intracranial wnd+<1hr loss consc
S624300	Extradural h'ge inj no open intracran wnd+1-24hr loss consc
S624400	Extradural h'ge inj no open intracran wnd+>24hr LOC+recovery
S624500	Extradural h'ge inj no open intracran wnd+>24hr LOC-restored

S624600	Extradural h'ge inj no open intracra wnd+LOC unspec duration
S624z00	Extradural h'ge inj no open intracran wnd+concussion unspec
S625.00	Open traumatic extradural haemorrhage
S625000	Extradural h'ge inj + open intracranial wnd + unspec consc
S625100	Extradural h'ge inj + open intracranial wound+no loss consc
S625200	Extradural h'ge inj + open intracranial wnd+<1hr loss consc
S625300	Extradural h'ge inj + open intracran wnd+1-24hr loss consc
S625400	Extradural h'ge inj + open intracran wnd+>24hr LOC+recovery
S625500	Extradural h'ge inj + open intracran wnd+>24hr LOC -restored
S625600	Extradural h'ge inj + open intracran wnd+LOC unspec duration
S625z00	Extradural h'ge inj + open intracran wnd+concussion unspec
S626.00	Epidural haemorrhage
S62A.00	Traumatic extradural haematoma
S62A000	Traumatic extradural haemat without open intracranial wound
S62A100	Traumatic extradural haematoma with open intracranial wound
S62z.00	Cerebral haemorrhage following injury NOS
S63..00	Other cerebral haemorrhage following injury
S630.00	Other cerebral h'ge after injury no open intracranial wound
S630.11	Cerebral compression due to injury
S630.12	Intracranial haematoma following injury
S630000	Oth cerebral h'ge inj no open intracran wnd+unspec consc
S630100	Oth cerebral h'ge inj no open intracranial wnd+no loss consc
S630200	Oth cerebral h'ge inj no open intracran wnd+<1hr loss consc
S630300	Oth cerebral h'ge inj no open intracran wnd+1-24hr LOC
S630400	Oth cereb h'ge inj no open intracran wnd+>24hr LOC +recovery
S630500	Oth cereb h'ge inj no open intracran wnd+>24hr LOC -restored
S630600	Oth cereb h'ge inj no open intracran wnd+LOC unspec duration
S630z00	Oth cereb h'ge inj no open intracran wnd+concussion unspec
S631.00	Other cerebral h'ge after injury + open intracranial wound
S631000	Oth cerebral h'ge inj + open intracran wnd + unspec consc
S631100	Oth cerebral h'ge inj + open intracranial wnd+no loss consc
S631200	Oth cerebral h'ge inj + open intracran wnd+<1hr loss consc
S631300	Oth cerebral h'ge inj + open intracran wnd+1-24hr loss consc

S631400	Oth cereb h'ge inj + open intracran wnd+>24hr LOC + recovery
S631500	Oth cereb h'ge inj + open intracran wnd+>24hr LOC -restored
S631600	Oth cereb h'ge inj + open intracran wnd+LOC unspec duration
S631z00	Oth cereb h'ge inj + open intracran wnd+concussion unspec
S63z.00	Other cerebral haemorrhage following injury NOS
**Heart Failure**	
14A6.00	H/O: heart failure
14AM.00	H/O: Heart failure in last year
1O1..00	Heart failure confirmed
388D.00	New York Heart Assoc classification heart failure symptoms
662f.00	New York Heart Association classification - class I
662g.00	New York Heart Association classification - class II
662h.00	New York Heart Association classification - class III
662i.00	New York Heart Association classification - class IV
662p.00	Heart failure 6 month review
662T.00	Congestive heart failure monitoring
662W.00	Heart failure annual review
679X.00	Heart failure education
67D4.00	Heart failure information given to patient
8B29.00	Cardiac failure therapy
8CL3.00	Heart failure care plan discussed with patient
8H2S.00	Admit heart failure emergency
8HBE.00	Heart failure follow-up
8Hg8.00	Discharge from practice nurse heart failure clinic
8HHb.00	Referral to heart failure nurse
8HHz.00	Referral to heart failure exercise programme
8Hk0.00	Referred to heart failure education group
8HTL.00	Referral to heart failure clinic
9N0k.00	Seen in heart failure clinic
9N2p.00	Seen by community heart failure nurse
9N4s.00	Did not attend practice nurse heart failure clinic
9N6T.00	Referred by heart failure nurse specialist
9Or..00	Heart failure monitoring administration

9Or0.00	Heart failure review completed
9Or1.00	Heart failure monitoring telephone invite
9Or2.00	Heart failure monitoring verbal invite
9Or3.00	Heart failure monitoring first letter
9Or4.00	Heart failure monitoring second letter
9Or5.00	Heart failure monitoring third letter
G232.00	Hypertensive heart&renal dis wth (congestive) heart failure
G234.00	Hyperten heart&renal dis+both(congestv)heart and renal fail
G1yz100	Rheumatic left ventricular failure
G5y4z00	Post cardiac operation heart failure NOS
G5yy900	Left ventricular systolic dysfunction
G5yyA00	Left ventricular diastolic dysfunction
L09y200	Cardiac failure following abortive pregnancy
Q48y100	Congenital cardiac failure
Q490.00	Neonatal cardiac failure
SP08400	Heart transplant failure and rejection
SP08500	Heart-lung transplant failure and rejection
SP11100	Cardiac insufficiency as a complication of care
SP11111	Heart failure as a complication of care
G58..00	Heart failure
G58..11	Cardiac failure
G580.00	Congestive heart failure
G580.11	Congestive cardiac failure
G580.12	Right heart failure
G580.13	Right ventricular failure
G580.14	Biventricular failure
G580000	Acute congestive heart failure
G580100	Chronic congestive heart failure
G580200	Decompensated cardiac failure
G580300	Compensated cardiac failure
G580400	Congestive heart failure due to valvular disease
G581.00	Left ventricular failure
G581.11	Asthma - cardiac

G581.12	Pulmonary oedema - acute
G581.13	Impaired left ventricular function
G581000	Acute left ventricular failure
G582.00	Acute heart failure
G583.00	Heart failure with normal ejection fraction
G583.11	HFNEF - heart failure with normal ejection fraction
G583.12	Heart failure with preserved ejection fraction
G584.00	Right ventricular failure
G58z.00	Heart failure NOS
G58z.11	Weak heart
G58z.12	Cardiac failure NOS
**Transient ischemic attack**	
8HBJ.00	Stroke / transient ischaemic attack referral
Fyu5500	[X]Other transnt cerebral ischaemic attacks+related syndroms
G65..00	Transient cerebral ischaemia
G65..11	Drop attack
G65..12	Transient ischaemic attack
G65..13	Vertebro-basilar insufficiency
G650.00	Basilar artery syndrome
G650.11	Insufficiency - basilar artery
G651.00	Vertebral artery syndrome
G651000	Vertebro-basilar artery syndrome
G652.00	Subclavian steal syndrome
G653.00	Carotid artery syndrome hemispheric
G654.00	Multiple and bilateral precerebral artery syndromes
G655.00	Transient global amnesia
G656.00	Vertebrobasilar insufficiency
G65y.00	Other transient cerebral ischaemia
G65z.00	Transient cerebral ischaemia NOS
G65z000	Impending cerebral ischaemia
G65z100	Intermittent cerebral ischaemia
G65zz00	Transient cerebral ischaemia NOS
**Coronary artery bypass graft**	

7922z00	Allograft replacement of coronary artery NOS
7922300	Allograft replacement of four or more coronary arteries
7922000	Allograft replacement of one coronary artery
7922200	Allograft replacement of three coronary arteries
7922100	Allograft replacement of two coronary arteries
7921300	Autograft replacement of four of more coronary arteries NEC
7921000	Autograft replacement of one coronary artery NEC
7921200	Autograft replacement of three coronary arteries NEC
7921100	Autograft replacement of two coronary arteries NEC
7925z00	Connection of mammary artery to coronary artery NOS
7925y00	Connection of mammary artery to coronary artery OS
7926z00	Connection of other thoracic artery to coronary artery NOS
7926y00	Connection of other thoracic artery to coronary artery OS
792..11	Coronary artery bypass graft operations
7926000	Double anastom thoracic arteries to coronary arteries NEC
7925000	Double anastomosis of mammary arteries to coronary arteries
7925100	Double implant of mammary arteries into coronary arteries
7926100	Double implant thoracic arteries into coronary arteries NEC
SP00300	Mechanical complication of coronary bypass
7921z00	Other autograft replacement of coronary artery NOS
7921y00	Other autograft replacement of coronary artery OS
792D.00	Other bypass of coronary artery
792Dz00	Other bypass of coronary artery NOS
792C.00	Other replacement of coronary artery
7922y00	Other specified allograft replacement of coronary artery
792Dy00	Other specified other bypass of coronary artery
7923y00	Other specified prosthetic replacement of coronary artery
792Cy00	Other specified replacement of coronary artery
7924y00	Other specified revision of bypass for coronary artery
7928200	Percut translum balloon angioplasty bypass graft coronary a
7923z00	Prosthetic replacement of coronary artery NOS
7923300	Prosthetic replacement of four or more coronary arteries
7923000	Prosthetic replacement of one coronary artery

7923200	Prosthetic replacement of three coronary arteries
7923100	Prosthetic replacement of two coronary arteries
792C000	Replacement of coronary arteries using multiple methods
792Cz00	Replacement of coronary artery NOS
7924z00	Revision of bypass for coronary artery NOS
7924300	Revision of bypass for four or more coronary arteries
7924000	Revision of bypass for one coronary artery
7924200	Revision of bypass for three coronary arteries
7924100	Revision of bypass for two coronary arteries
7924400	Revision of connection of thoracic artery to coronary artery
7920z00	Saphenous vein graft replacement coronary artery NOS
7920y00	Saphenous vein graft replacement of coronary artery OS
7920300	Saphenous vein graft replacement of four+ coronary arteries
7920000	Saphenous vein graft replacement of one coronary artery
7920200	Saphenous vein graft replacement of three coronary arteries
7920100	Saphenous vein graft replacement of two coronary arteries
7925200	Single anast mammary art to left ant descend coronary art
7925300	Single anastomosis of mammary artery to coronary artery NEC
7926200	Single anastomosis of thoracic artery to coronary artery NEC
7925400	Single implantation of mammary artery into coronary artery
7926300	Single implantation thoracic artery into coronary artery NEC
7927200	Transection of muscle bridge of coronary artery
7927300	Transposition of coronary artery NEC
ZV45700	[V]Presence of aortocoronary bypass graft
ZV45K00	[V]Presence of coronary artery bypass graft
ZV45K00	[V]Presence of coronary artery bypass graft
ZV45K11	[V]Presence of coronary artery bypass graft - CABG
ZV45K11	[V]Presence of coronary artery bypass graft - CABG
7925011	LIMA sequential anastomosis
7925012	RIMA sequential anastomosis
7925311	LIMA single anastomosis
7925312	RIMA single anastomosis
7935600	Perc trans three dimen electroanat mapping conduct sys heart

7927000	Repair of arteriovenous fistula of coronary artery
7927100	Repair of aneurysm of coronary artery
7927y00	Other specified other open operation on coronary artery
7927z00	Other open operation on coronary artery NOS
792B000	Endarterectomy of coronary artery NEC
792B.00	Repair of coronary artery NEC
792By00	Other specified repair of coronary artery
792Bz00	Repair of coronary artery NOS
792y.00	Other specified operations on coronary artery
792z.00	Coronary artery operations NOS
7927.00	Other open operations on coronary artery
7927.00	Other open operations on coronary artery
7924.00	Revision of bypass for coronary artery
7923.00	Prosthetic replacement of coronary artery
7923.11	Prosthetic bypass of coronary artery
7921.00	Other autograft replacement of coronary artery
7925.11	Creation of bypass from mammary artery to coronary artery
7925.00	Connection of mammary artery to coronary artery
7926.00	Connection of other thoracic artery to coronary artery
7922.00	Allograft replacement of coronary artery
**Percutaneous coronary intervention**	
7927500	Open angioplasty of coronary artery
7928000	Percut transluminal balloon angioplasty one coronary artery
7928.00	Transluminal balloon angioplasty of coronary artery
7928100	Percut translum balloon angioplasty mult coronary arteries
7928.11	Percutaneous balloon coronary angioplasty
7928y00	Transluminal balloon angioplasty of coronary artery OS
7928z00	Transluminal balloon angioplasty of coronary artery NOS
7929000	Percutaneous transluminal laser coronary angioplasty
7929.00	Other therapeutic transluminal operations on coronary artery
7929100	Percut transluminal coronary thrombolysis with streptokinase
7929111	Percut translum coronary thrombolytic therapy- streptokinase
7929200	Percut translum inject therap subst to coronary artery NEC

7929300	Rotary blade coronary angioplasty
7929400	Insertion of coronary artery stent
7929y00	Other therapeutic transluminal op on coronary artery OS
7929z00	Other therapeutic transluminal op on coronary artery NOS
ZV45800	[V]Presence of coronary angioplasty implant and graft
ZV45L00	[V]Status following coronary angioplasty NOS
793G.00	Perc translumin balloon angioplasty stenting coronary artery
793K.00	Transluminal operations internal mammary artery side branch
7933.00	Transluminal heart assist operations
7934.00	Other therapeutic transluminal operations on heart
7935.00	Diagnostic transluminal operations on heart
793Gy00	OS perc translumina balloon angioplast stenting coronary art
793Gz00	Perc translum balloon angioplasty stenting coronary art NOS
793Ky00	OS transluminal operations internal mammary art side branch
793Kz00	Transluminal operations internal mammary art side branch NOS
793G000	Perc translum ball angio insert 1-2 drug elut stents cor art
793H000	Percutaneous transluminal balloon dilation cardiac conduit
793K000	Translum occlusion left internal mammary artery side branch
793G100	Perc tran ball angio ins 3 or more drug elut stents cor art
793G200	Perc translum balloon angioplasty insert 1-2 stents cor art
793G300	Percutaneous cor balloon angiop 3 more stents cor art NEC
7934y00	Other therapeutic transluminal operation on heart OS
7934z00	Other therapeutic transluminal operation on heart NOS
7935y00	Other specified diagnostic transluminal operation on heart
7935z00	Diagnostic transluminal operation on heart NOS
7928300	Percut translum cutting balloon angioplasty coronary artery
7929500	Insertion of drug-eluting coronary artery stent
7929600	Percutaneous transluminal atherectomy of coronary artery
7933000	Transluminal insertion of pulsation balloon into aorta
7928200	Percut translum balloon angioplasty bypass graft coronary a
**Peripheral artery disease**	
700.11	Aorto-iliac disease
G702.00	Extremity artery atheroma

G702z00	Extremity artery atheroma NOS
G73..11	Peripheral ischaemic vascular disease
G73..12	Ischaemia of legs
G73..13	Peripheral ischaemia
G730100	Raynaud's phenomenon
G731000	Buerger's disease
G731100	Presenile gangrene
G732.00	Peripheral gangrene
G732000	Gangrene of toe
G732100	Gangrene of foot
G733.00	Ischaemic foot
G734.00	Peripheral arterial disease
G73y.00	Other specified peripheral vascular disease
G73y000	Diabetic peripheral angiopathy
G73y100	Peripheral angiopathic disease EC NOS
G73yz00	Other specified peripheral vascular disease NOS
G73z.00	Peripheral vascular disease NOS
G73z000	Intermittent claudication
G73z011	Claudication
G73z012	Vascular claudication
G73z100	Spasm of peripheral artery
G73zz00	Peripheral vascular disease NOS
G74..00	Arterial embolism and thrombosis
G74..11	Arterial embolus and thrombosis
G74..12	Thrombosis - arterial
G74..13	Arterial embolic and thrombotic occlusion
G740.12	Aortoiliac obstruction
G740.13	Leriche's syndrome
G742400	Embolism and thrombosis of the femoral artery
G742500	Embolism and thrombosis of the popliteal artery
G742600	Embolism and thrombosis of the anterior tibial artery
G742700	Embolism and thrombosis of the dorsalis pedis artery
G742800	Embolism and thrombosis of the posterior tibial artery

G742900	Embolism and thrombosis of a leg artery NOS
G742B00	Post radiological embolism of lower limb artery
G742z00	Peripheral arterial embolism and thrombosis NOS
G74y000	Embolism and/or thrombosis of the common iliac artery
G74y100	Embolism and/or thrombosis of the internal iliac artery
G74y200	Embolism and/or thrombosis of the external iliac artery
G74y300	Embolism and thrombosis of the iliac artery unspecified
G74z.00	Arterial embolism and thrombosis NOS
G761.00	Stricture of artery
G765.00	Necrosis of artery
G76A.00	Arterial insufficiency
G76z.00	Disorders of arteries and arterioles NOS
G76z000	Iliac artery occlusion
G76z100	Femoral artery occlusion
G76z200	Popliteal artery occlusion
G7y..00	Other specified arterial, arteriole or capillary disease
G7z..00	Arterial, arteriole and capillary diseases NOS
Rango 14NB.00	H/O: Peripheral vascular disease procedure
2I16.00	O/E - gangrene
R054.00	[D]Gangrene
R054200	[D]Gangrene of toe in diabetic
R054300	[D]Widespdiabetic foot gangrene
8HIP.00	Referred for peripheral artery disease assessment
7A12100	Bypass bifurc aorta by anastom aorta to femoral artery NEC
7A12300	Bypass bifurcation aorta by anastom aorta to iliac artery
7A4..00	Iliac and femoral artery operations
7A41.00	Other bypass of iliac artery
7A41.11	Other bypass of iliac artery by anastomosis
7A41100	Bypass iliac artery by iliac/femoral artery anastomosis NEC
7A41200	Emerg bypass iliac artery by femoral/femoral art anast NEC
7A41300	Bypass iliac artery by femoral/femoral art anastomosis NEC
7A41500	Emerg bypass iliac artery by aorta/ext iliac art anast NEC

7A41600	Emerg bypass leg artery by aorta/com fem art anastomosis NEC
7A41700	Emerg bypass leg artery by aorta/deep fem anastomosis NEC
7A41800	Emerg bypass iliac artery by iliac/iliac art anastomosis NEC
7A41900	Bypass common iliac artery by aorta/com iliac art anast NEC
7A41A00	Bypass iliac artery by aorta/ext iliac art anastomosis NEC
7A41B00	Bypass leg artery by aorta/com femoral art anastomosis NEC
7A41C00	Bypass leg artery by aorta/deep femoral art anastomosis NEC
7A41D00	Bypass iliac artery by iliac/iliac artery anastomosis NEC
7A41E00	Emergency bypass of iliac artery by unspecified anastomosis
7A41y00	Other specified other bypass of iliac artery
7A41z00	Other bypass of iliac artery NOS
7A42.00	Reconstruction of iliac artery
7A42.11	Reconstruction of common iliac artery
7A42000	Endarterectomy and patch repair of iliac artery
7A42011	Endarterectomy and patch repair of common iliac artery
7A42100	Endarterectomy of iliac artery NEC
7A42111	Endarterectomy of common iliac artery NEC
7A42y00	Other specified reconstruction of iliac artery
7A42z00	Reconstruction of iliac artery NOS
7A43.00	Other open operations on iliac artery
7A43.11	Other open operations on common iliac artery
7A43000	Repair of iliac artery NEC
7A43011	Repair of common iliac artery NEC
7A43100	Open embolectomy of iliac artery
7A43111	Open embolectomy of common iliac artery
7A43300	Open insertion of iliac artery stent
7A43y00	Other specified other open operation on iliac artery
7A43z00	Other open operation on iliac artery NOS
7A44.00	Transluminal operations on iliac artery
7A44.11	Transluminal operations on common iliac artery
7A44000	Percutaneous transluminal angioplasty of iliac artery
7A44100	Percutaneous transluminal embolectomy of iliac artery
7A44200	Arteriography of iliac artery

7A44211	Arteriography of common iliac artery
7A44300	Insertion of iliac artery stent
7A44400	Percutaneous transluminal insertion of iliac artery stent
7A44y00	Other specified transluminal operation on iliac artery
7A44z00	Transluminal operation on iliac artery NOS
7A47.00	Other emergency bypass of femoral artery or popliteal artery
7A47.11	Other emerg bypass femoral or popliteal art by anastomosis
7A47.12	Other emergency bypass of common femoral artery
7A47.13	Other emergency bypass of deep femoral artery
7A47.14	Other emergency bypass of popliteal artery
7A47.15	Other emergency bypass of superficial femoral artery
7A47.16	Other emergency bypass of femoral artery
7A47100	Emerg bypass popliteal art by pop/pop art anast c prosth NEC
7A47500	Emerg bypass popliteal art by pop/tib art anast c prosth NEC
7A47700	Emerg bypass pop art by pop/tib art anast c vein graft NEC
7A47900	Emerg bypass popliteal art by pop/peron a anast c prosth NEC
7A47C00	Emerg bypass femoral artery by fem/fem art anastomosis NEC
7A47D00	Emerg bypass popliteal artery by pop/fem art anastomosis NEC
7A47y00	Other emergency bypass of femoral or popliteal artery OS
7A47z00	Other emergency bypass of femoral or popliteal artery NOS
7A48.00	Other bypass of femoral artery or popliteal artery
7A48.11	Other bypass of femoral or popliteal artery by anastomosis
7A48.12	Other bypass of common femoral artery
7A48.13	Other bypass of deep femoral artery
7A48.14	Other bypass of femoral artery
7A48.15	Other bypass of popliteal artery
7A48000	Bypass femoral artery by fem/pop art anast c prosthesis NEC
7A48100	Bypass popliteal artery by pop/pop a anast c prosthesis NEC
7A48200	Bypass femoral artery by fem/pop art anast c vein graft NEC
7A48300	Bypass popliteal artery by pop/pop a anast c vein graft NEC
7A48400	Bypass femoral artery by fem/tib art anast c prosthesis NEC
7A48500	Bypass popliteal artery by pop/tib a anast c prosthesis NEC
7A48600	Bypass femoral artery by fem/tib art anast c vein graft NEC

7A48700	Bypass popliteal artery by pop/tib a anast c vein graft NEC
7A48800	Bypass femoral artery by fem/peron a anast c prosthesis NEC
7A48900	Bypass popliteal artery by pop/peron art anast c prosth NEC
7A48A00	Bypass femoral artery by fem/peron a anast c vein graft NEC
7A48B00	Bypass popliteal art by pop/peron art anast c vein graft NEC
7A48C00	Bypass femoral artery by femoral/femoral art anastomosis NEC
7A48D00	Bypass popliteal artery by pop/fem artery anastomosis NEC
7A48y00	Other bypass of femoral artery or popliteal artery OS
7A48z00	Other bypass of femoral artery or popliteal artery NOS
7A49.00	Reconstruction of femoral artery or popliteal artery
7A49.11	Reconstruction of common femoral artery
7A49.12	Reconstruction of deep femoral artery
7A49.13	Reconstruction of femoral artery
7A49.14	Reconstruction of popliteal artery
7A49.15	Reconstruction of superficial femoral artery
7A49000	Endarterectomy and patch repair of femoral artery
7A49100	Endarterectomy and patch repair of popliteal artery
7A49200	Endarterectomy of femoral artery NEC
7A49300	Endarterectomy of popliteal artery NEC
7A49400	Profundoplasty femoral artery & patch repair deep fem artery
7A49500	Profundoplasty and patch repair of popliteal artery
7A49600	Profundoplasty of femoral artery NEC
7A49700	Profundoplasty of popliteal artery NEC
7A49800	Reconstruction of femoral artery with vein graft
7A49900	Reconstruction of popliteal artery with vein graft
7A49y00	Reconstruction of femoral or popliteal artery OS
7A49z00	Reconstruction of femoral or popliteal artery NOS
7A4A.00	Other open operations on femoral artery or popliteal artery
7A4A.11	Other open operations on common femoral artery
7A4A.12	Other open operations on deep femoral artery
7A4A.13	Other open operations on popliteal artery
7A4A.14	Other open operations on superficial femoral artery
7A4A000	Repair of femoral artery NEC

7A4A100	Repair of popliteal artery NEC
7A4A200	Open embolectomy of femoral artery
7A4A211	Open thrombectomy of femoral artery
7A4A300	Open embolectomy popliteal artery
7A4A311	Open thrombectomy of popliteal artery
7A4A600	Operation on popliteal artery NEC
7A4A700	Repair of femoral artery with temporary silastic shunt
7A4A800	Repair of popliteal artery with temporary silastic shunt
7A4Ay00	Other open operation on femoral or popliteal artery OS
7A4Az00	Other open operation on femoral or popliteal artery NOS
7A4B.00	Transluminal operations on femoral or popliteal artery
7A4B.11	Transluminal procedure on common femoral artery
7A4B.12	Transluminal procedure on deep femoral artery
7A4B.13	Transluminal procedure on femoral artery
7A4B.14	Transluminal procedure on popliteal artery
7A4B.15	Transluminal procedure on superficial femoral artery
7A4B000	Percutaneous transluminal angioplasty of femoral artery
7A4B100	Percutaneous transluminal angioplasty of popliteal artery
7A4B200	Percutaneous transluminal embolectomy of femoral artery
7A4B300	Percutaneous transluminal embolectomy of popliteal artery
7A4B400	Percutaneous transluminal embolisation of femoral artery
7A4B500	Percutaneous transluminal embolisation of popliteal artery
7A4B900	Percutaneous transluminal insertion of stent femoral artery
7A4By00	Transluminal operation on femoral or popliteal artery OS
7A4Bz00	Transluminal operation on femoral or popliteal artery NOS
7A4y.00	Other specified operations on iliac and femoral artery
7A4z.00	Iliac and femoral artery operations NOS
7A50100	Revision of reconstruction involving iliac artery
7A50200	Revision of reconstruction involving femoral artery
7A50300	Revision of reconstruction of popliteal artery
7A53400	Operation on aneurysm of artery NEC
7A56.00	Other therapeutic transluminal operations on artery
7A56000	Percutaneous transluminal arterial thrombolysis reconstruct

7A56100	Percutaneous transluminal stent reconstruction of artery
7A56400	Percutaneous transluminal balloon angioplasty of artery
7A56600	Percutaneous transluminal placement peripheral stent artery
7A56y00	Other specified other therapeutic transluminal operat artery
7A56z00	Other therapeutic transluminal operations on artery NOS
7M1D300	Transluminal approach to organ through femoral artery
7M25M00	Deep circumflex iliac artery flap
7N46000	[SO]Common iliac artery
7N46100	[SO]Internal iliac artery
7N46200	[SO]Common femoral artery
7N46300	[SO]Deep femoral artery
7N46311	[SO]Profunda femoris artery
7N46400	[SO]Superficial femoral artery
7N46500	[SO]Popliteal artery
7N46600	[SO]Tibial artery
7N46700	[SO]External iliac artery
7N46800	[SO]Deep circumflex iliac artery
7N46B00	[SO]Other artery of thigh
7N46C00	[SO]Anterior tibial artery
7N46D00	[SO]Posterior tibial artery
7N46F00	[SO]Medial plantar artery
7N46G00	[SO]Lateral plantar artery
7N46H00	[SO]Dorsalis pedis artery
7N46J00	[SO]Digital artery of toe
7NB8000	[SO]Anterior tibial artery
7NB8100	[SO]Posterior tibial artery
7NB8300	[SO]Dorsalis pedis artery
7NB8400	[SO]External iliac artery
7NB8500	[SO]Iliac artery NEC
7NB8600	[SO]Tibial artery NEC
F336.00	Phantom limb syndrome
F336000	Phantom limb syndrome with pain
F336100	Phantom limb syndrome without pain

Table S3. Comparison of post-propensity match baseline characteristics between the US Truven MarketScan mortality subset and US Truven MarketScan total population

	US Truven MarketScan Mortality subset				US Truven MarketScan Total data				Difference Mortality vs Total	
	SGLT-2i (N=71,632)		oGLD (N=71,632)		SGLT-2i (N=116,899)		oGLD (N=116,899)		SGLT-2i	oGLD
	n	%	n	%	n	%	n	%	%	%
**Age in years, mean (SD)**	55.8 (9.5)		56.0 (10.2)		55.76 (9.68)		55.79 (10.24)			
**Women**	31,715	44.3	32,151	44.9	53,293	45.6	53,806	46.0	-1.3	-1.1
**Frailty (yes)**	2,869	4.0	3,295	4.6	5,340	4.6	5,382	4.6	-0.6	0.0
**Established CVD***	6,452	9.0	6,918	9.7	11,218	9.6	11,292	9.7	-0.6	0.0
**Acute MI**	576	0.8	630	0.9	1,052	0.9	1,036	0.9	-0.1	0.0
**Unstable angina**	711	1.0	756	1.1	1,222	1.0	1,227	1.0	0.0	0.1
**Heart failure**	1,638	2.3	1,845	2.6	3,025	2.6	3,039	2.6	-0.3	0.0
**Atrial fibrillation**	1,892	2.6	2,085	2.9	3,308	2.8	3,330	2.8	-0.2	0.1
**Stroke**	2,413	3.4	2,615	3.7	4,165	3.6	4,219	3.6	-0.2	0.1
**PAD**	1,835	2.6	1,884	2.6	3,119	2.7	3,128	2.7	-0.1	-0.1
**Microvascular disease**	18,673	26.1	18,896	26.4	31,454	26.9	31,548	27.0	-0.8	-0.6
**CKD**	1,742	2.4	2,112	2.9	3,305	2.8	3,501	3.0	-0.4	-0.1
**Statin therapy**	48,567	67.8	48,120	67.2	78,061	66.8	77,992	66.7	1.0	0.5
**Antihypertensive therapy**	59,172	82.6	58,711	82.0	95,336	81.6	95,247	81.5	1.0	0.5
**Loop diuretics**	5,581	7.8	5,768	8.1	9,504	8.1	9,452	8.1	-0.3	0.0
**Metformin**	59,007	82.4	57,210	79.9	92,569	79.2	93,369	79.9	3.2	0.0
**SU**	29,943	41.8	28,973	40.4	47,582	40.7	47,771	40.9	1.1	-0.5
**DPP-4 inhibitor**	26,876	37.5	24,681	34.5	41,970	35.9	40,683	34.8	1.6	-0.3
**TZD**	7,890	11.0	7,282	10.2	12,497	10.7	11,872	10.2	0.3	0.0
**GLP-1RA**	14,880	20.8	11,916	16.6	23,513	20.1	19,843	17.0	0.7	-0.4
**Insulin**	21,548	30.1	20,411	28.5	34,844	29.8	34,251	29.3	0.3	-0.8
**Index year:**										
**2013**	9,266	12.9	12,811	17.9	16,426	14.1	21,068	18.0	-1.2	-0.1
**2014**	34,832	48.6	27,152	37.9	58,305	49.9	46,303	39.6	-1.3	-1.7
**2015**	27,534	38.4	31,669	44.2	42,168	36.1	49,528	42.4	2.3	1.8
**Index medication:**										
**Amylin analog**	NA	NA	78	0.10	NA	NA	132	0.10		0.0
**Metformin**	NA	NA	8,335	11.6	NA	NA	13,624	11.7		-0.1
**SU**	NA	NA	12,781	17.8	NA	NA	20,725	17.7		0.1

**DPP-4i**	NA	NA	11,525	16.1	NA	NA	18,350	15.7		0.4
**TZD**	NA	NA	4,200	5.9	NA	NA	6,954	5.9		0.0
**GLP-1RA**	NA	NA	9,940	13.9	NA	NA	16,337	14.0		-0.1
**Insulin**	NA	NA	23,438	32.7	NA	NA	38,529	33.0		-0.3
**Meglitinides**	NA	NA	986	1.4	NA	NA	1,623	1.4		0.0
**Acarbose,**	NA	NA	349	0.5	NA	NA	625	0.5		0.0
**Dapagliflozin**	16,091	22.5	NA	NA	25,940	0.222	NA	NA	0.3	
**Empagliflozin**	5,783	8.1	NA	NA	9,097	0.078	NA	NA	0.3	
**Canagliflozin**	49,758	69.5	NA	NA	81,862	0.7	NA	NA	-0.5	

*Defined as myocardial infarction, unstable angina, stroke, heart failure, transient ischemic attack, coronary revascularization (coronary artery bypass grafting or percutaneous coronary intervention) or occlusive peripheral artery disease on or ever prior to start; CKD=chronic kidney disease; CVD=cardiovascular disease; DPP-4=dipeptidyl peptidase-4; oGLD=other glucose-lowering drug; GLP-1RA= Glucagon-like peptide-1 receptor agonists; MI=myocardial infarction; NA=not applicable; PAD=peripheral arterial disease; SGLT-2i=sodium-glucose cotransporter-2 inhibitor; SU=sulfonylurea; TZD=thiazolidinedione; US=United States

Table S4. List of variables used to develop propensity score

Gender
Age
Race (Caucasian, Black, Asian, Other)
Migrant
Frailty (yes)
Index year
Duration of Type 2 diabetes (years)
Cardiovascular history
Smoker
HbA1c >7% (UK only)
Body mass index ≥30 kg/m^2 (UK only)
Duration of Type 2 diabetes
Estimated Glomerular Filtration Rate <60 mL/min/1.73 m^2 (UK only)
Acute myocardial infarction
Stroke
Heart failure
Unstable angina
Atrial fibrillation
Peripheral artery disease
Hypertension
Coronary revascularization
Coronary artery bypass grafting
Percutaneous coronary intervention
Carotid intervention
Chronic kidney disease
Microvascular disease
Nephropathy
Peripheral neuropathy

Retinopathy
Bariatric surgery
Metformin
Sulfonylurea
DPP-4 inhibitor
thiazolidinedione
GLP-1 receptor agonist
Insulin
Statin therapy
Antihypertensive therapy
P2Y12 inhibitiors
Antiplatelets
Warfarin
Anticoagulants
Low dose acetylsalicylic acid
Beta-blocker
Loop diuretics
Thiazides
Aldosterone antagonists
Weight loss drugs

DPP-4=dipeptidyl peptidase-4; GLP-1= Glucagon-like peptide-1.

The full list of variables for each individual country is dependent on the variables available in each database

Table S5. Baseline characteristics for all countries/databases pre-match

	US Truven MarketScan		Norway national registers		Denmark national registers		Sweden national registers		UK CPRD/THIN		Germany DPV	
	SGLT-2i N=123,648	oGLD N=712,426	SGLT-2i N=14,438	oGLD N=96,947	SGLT-2i N=9522	oGLD N=119,137	SGLT-2i N=9337	oGLD N=200,284	SGLT-2i N=7556	oGLD N=73,436	SGLT-2i N=1532	oGLD N=23,991
Age, years, mean	55.7	58.1	59.7	61.2	60.3	62.3	61.4	65.4	58.5	63.6	61.5	69.5
Women	45.5	46.7	39.4	43.6	39.5	44.6	37.7	42.5	41.2	42.8	42.0	47.6
Established CVD*	9.5	15.3	19.3	22.4	29.6	32.0	24.5	30.3	15.2	22.1	35.1	48.2
Acute MI	0.9	1.9	5.9	6.6	7.6	8.4	9.4	10.4	5.9	8.2	10.0	11.1
Unstable angina	1.0	1.5	3.4	3.0	3.5	3.5	4.8	4.7	1.5	1.7	3.4	5.3
HF	2.5	6.1	4.1	6.9	3.9	5.3	6.2	9.8	2.3	5.1	5.3	9.4
AF	2.8	5.5	6.1	9.3	5.6	7.7	7.5	11.6	3.7	7.7	5.2	8.2
Stroke	3.5	6.1	2.9	4.4	7.2	9.5	5.3	8.7	3.1	5.5	5.3	9.3
PAD	2.6	3.9	5.6	6.5	5.0	6.4	4.7	5.8	3.0	4.3	21.5	31.5
Microvascular disease	27.2	23.6	31.2	22.6	34.9	19.0	13.7	12.0	34.4	23.4	50.8	49.5
CKD	2.7†	5.9†	1.5	5.5	0.5	2.5	0.9	3.5	0.7†	2.3†	19.6	46.5
Frailty(yes)	4.4	11.5	11.3	21.7	30.9	31.3	11.5	22.2	17.2‡	22.7‡	19.8	47.7
Glucose-lowering therapies												
MET	80.2	41.5	70.6	35.3	78.9	36.2	80.2	37.4	90.5	51.1	71.3	45.3
SU	41.2	22.9	36.8	15.0	28.4	12.6	23.2	11.9	51.6	27.0	10.1	14.5
DPP-4i	36.9	13.1	22.4	6.1	20.8	4.6	25.4	5.1	44.1	10.9	31.2	20.4
TZD	11.1	4.9	2.3	0.6	0.5	0.1	3.2	0.6	10.7	5.7	4.8	0.9
GLP-1RA	22.4	4.6	15.8	2.5	35.3	5.0	21.6	2.2	22.9	3.2	17.5	4.0
Insulin	30.8	17.2	19.0	18.4	28.7	16.5	44.4	25.1	21.6	5.1	48.4	61.8
Cardiovascular therapies												
Antihypertensive	81.8	77.3	69.9	62.2	79.1	64.5	79.4	72.2	75.7	73.6	65.3	56.7

**therapies§**												
**Loop diuretics**	8.1	11.5	10.1	15.0	13.5	18.2	15.2	18.5	11.6	14.1	5.2	7.1
**Thiazide diuretics**	–	–	1.9	2.4	16.3	15.0	7.4	8.0	17.5	18.1	27.7	30.0
**Statin therapy**	67.2	58.7	62.4	49.4	75.6	52.1	68.6	53.5	82.1	73.4	39.4	29.8
**Index year**												
**2012**	–	–	–	–	0.2	2.8	–	–	0	6.74	0.07	21.8
**2013**	13.5	38.2	9.0	24.8	20.1	31.7	8.2	16.8	11.9	38.1	10.5	38.5
**2014**	49.1	39.5	32.7	28.4	31.4	31.1	33.4	38.5	41.8	31.8	24.0	19.2
**2015**	37.4	22.4	35.6	30.1	48.2	34.5	58.5	44.8	45.9	23.1	33.5	13.7
**2016**	–	–	22.7	16.7	–	–	–	–	0.4	0.2	32.0	6.8

Data are % unless otherwise stated; *Defined as myocardial infarction, unstable angina, stroke, heart failure, transient ischemic attack, coronary revascularization (coronary artery bypass grafting or percutaneous coronary intervention) or occlusive peripheral artery disease on or ever prior to start †CKD in the indicated database does not include end stage renal disease; ‡Frailty in the indicated database is defined as ≥1 hospitalization within 1 year prior to or on index date; §Includes angiotensin converting enzyme inhibitors, angiotensin receptor blockers, Ca^{2+} channel blockers β-blockers, thiazides; AF=atrial fibrillation; CKD=chronic kidney disease; CPRD, Clinical Practice Research Datalink; CVD=cardiovascular disease; DPP-4i=dipeptidyl peptidase-4 inhibitor; DPV, Diabetes Patientenverlaufsdokumentation; eGFR=estimated Glomerular Filtration Rate; HF=heart failure; oGLD=other glucose-lowering drug; GLP-1RA= Glucagon-like peptide-1 receptor agonists; MET=metformin; MI, myocardial infarction; PAD=peripheral arterial disease; SGLT-2i=sodium-glucose cotransporter-2 inhibitor; SU=sulfonylurea; THIN, The Health Improvement Network; TZD, thiazolidinedione

Table S6. Baseline characteristics for all countries/databases post-match

	US Truven MarketScan		Norway national registers		Denmark national registers		Sweden national registers		UK CPRD/THIN		Germany DPV	
	SGLT-2i N=116,899	oGLD N=116,899	SGLT-2i N=12,525	oGLD N=12,525	SGLT-2i N=9234	oGLD N=9234	SGLT-2i N=9189	oGLD N=9189	SGLT-2i N=5231	oGLD N=5231	SGLT-2i N=1450	oGLD N=1450
Age, years, mean (SD)	55.8 (9.7)	55.8 (10.2)	60.4 (11.3)	60.3 (12.7)	60.4 (10.8)	60.9 (12.4)	61.5 (10.2)	61.4 (11.5)	58.8 (10.5)	58.8 (10.8)	61.7 (11.2)	61.7 (11.7)
Women	45.6	46.0	41.4	39.8	39.7	39.8	37.8	37.8	41.9	42.4	42.7	42.3
Established CVD*	9.6	9.7	20.0	20.4	29.6	30.4	24.5	24.6	15.7	16.2	35.5	37.1
Acute MI	0.9	0.9	5.9	6.1	7.7	8.0	9.3	9.3	5.6	6.4	9.8	10.3
Unstable angina	1.0	1.0	3.4	3.4	3.4	3.7	4.7	4.7	1.4	1.6	3.6	3.8
HF	2.6	2.6	4.4	4.4	3.9	4.2	6.2	6.2	2.4	2.5	5.5	5.7
AF	2.8	2.8	6.6	6.7	5.7	5.9	7.5	7.7	4.1	3.6	5.2	5.5
Stroke	3.6	3.6	4.6	4.4	7.3	7.5	7.3	7.2	3.3	3.7	5.6	5.9
PAD	2.7	2.7	5.9	5.8	5.1	5.3	4.8	4.6	3.0	2.8	21.7	21.3
Microvascular disease	26.9	27.0	31.4	32.0	34.3	33.0	13.5	12.8	32.5	32.4	49.7	51.6
CKD	2.8†	3.0†	1.8	1.7	0.5	0.4	0.9	0.8	0.8†	0.8†	19.5	28.4
Frailty(yes)	4.6	4.6	12.2	12.9	30.7	30.7	11.6	11.7	17.5‡	20.5‡	20.0	51.7
Glucose-lowering therapies												
MET	79.2	79.9	69.2	73.4	78.3	81.4	80.0	82.8	88.9	89.8	71.2	72.1
SU	40.7	40.9	34.4	34.2	28.0	29.2	23.0	24.1	50.9	51.4	10.3	9.6
DPP-4i	35.9	34.8	21.0	21.5	20.1	19.3	25.0	24.9	41.8	41.1	31.6	33.2
TZD	10.7	10.2	2.1	2.0	–	–	3.0	2.6	10.8	10.8	3.4	3.1
GLP-1RA	20.1	17.0	13.6	12.7	33.6	31.0	20.6	18.3	17.4	17.2	16.0	15.2
Insulin	29.8	29.3	20.0	21.6	27.8	28.6	43.8	42.7	17.5	16.6	49.3	48.6
Cardiovascular therapies												
Antihypertensive therapy§	81.6	81.5	70.9	70.8	78.9	78.5	79.6	79.7	75.4	75.4	64.5	65.0
Loop diuretics	8.1	8.1	11.0	11.3	13.6	14.1	15.2	14.9	12.8	13.2	5.2	5.5
Thiazide diuretics	33.1	33.1	2.0	2.1	16.3	16.3	7.4	7.6	16.9	16.9	27.9	28.5

**ACE inhibitors**	46.6	46.6	18.9	19.8	39.7	40.6	36.5	36.6	49.0	49.7	30.0	32.0
**ARBs**	30.0	29.8	45.3	44.6	34.8	35.1	38.2	38.4	19.5	18.9	19.2	15.8
**Statin therapy**	66.8	66.7	62.4	62.6	75.3	77.8	68.4	68.5	82.1	81.2	38.3	38.8
**Aspirin**	NA	NA	38.1	37.9	37.4	38.4	34.6	34.4	31.2	32.4	18.3	19.5
**Index year**												
**2012**	–	–	–	–	0.2	1.2	–	–	0	1.6	0.1	5.1
**2013**	14.1	18.0	10.0	9.9	20.6	20.0	8.2	7.9	15.0	13.1	10.8	10.6
**2014**	49.9	39.6	32.9	32.5	31.6	29.8	33.5	34.0	43.6	44.1	24.8	16.1
**2015**	36.1	42.4	35.1	35.3	47.5	48.9	58.3	58.1	41.2	41.0	33.9	38.0
**2016**	–	–	21.9	22.4	–	–	–	–	0.2	0.3	30.5	30.3

Data are % unless otherwise stated; *Defined as myocardial infarction, unstable angina, stroke, heart failure, transient ischemic attack, coronary revascularization (coronary artery bypass grafting or percutaneous coronary intervention) or occlusive peripheral artery disease on or ever prior to start †CKD in the indicated database does not include end stage renal disease; ‡Frailty in the indicated database is defined as ≥1 hospitalization within 1 year prior to or on index date; §Includes ACE inhibitors, ARBs, Ca^{2+} channel blockers β-blockers, thiazides; ACE=angiotensin converting enzyme; ARB=angiotensin receptor blocker; AF=atrial fibrillation; CKD=chronic kidney disease; CPRD, Clinical Practice Research Datalink; CV=cardiovascular; CVD=cardiovascular disease; DPP-4i=dipeptidyl peptidase-4 inhibitor; DPV, Diabetes Patientenverlaufsdokumentation; eGFR=estimated Glomerular Filtration Rate; HF=heart failure; oGLD=other glucose-lowering drug; GLP-1RA= Glucagon-like peptide-1 receptor agonists; MET=metformin; MI, myocardial infarction; PAD=peripheral arterial disease; SGLT-2i=sodium-glucose cotransporter-2 inhibitor; SU=sulfonylurea; THIN, The Health Improvement Network; TZD, thiazolidinedione

Table S7. Standardized differences in baseline characteristics between SGLT-2i and oGLD treatment groups pre- and post-propensity match by country

	Standardized Difference (%)											
	US Truven MarketScan (N=233,798)		Norway national registers (N=25,050)		Denmark national registers (N=18,468)		Sweden national registers (N=18,378)		UK CPRD/THIN (N=10,462)		Germany DPV (N=2900)	
	Pre-match	Post-match	Pre-match	Post-match	Pre-match	Post-match	Pre-match	Post-match	Pre-match	Post-match	Pre-match	Post-match
Age	21.1	0.3	11.3	0.8	15.3	4.3	33.2	0.6	43.1	0	27.2	0.1
Women	2.4	0.9	6.8	2.6	8.5	0.2	7.9	0	3.4	1.0	4.3	0.2
Established CVD*	NA	NA	6.0	0.8	4.3	1.4	10.4	0.1	17.7	1.5	10.4	0.9
Acute MI	8.7	0.1	2.4	0.7	2.4	0.9	2.9	0.1	8.9	3.6	1.3	0.4
Unstable angina	4.5	0	2.0	0.1	0	1.3	0.2	0	2.0	1.7	4.0	0.3
Heart failure	17.5	0.1	9.8	0.3	5.6	1.3	10.5	0.2	14.9	0.1	6.8	0.2
Stroke	11.9	0.2	6.3	0.9	6.9	0.6	10.7	0.9	12.0	2.0	6.7	0.3
PAD	6.8	0	3.1	0.4	5.0	0.7	3.8	0.5	6.6	1.0	9.2	0.3
Coronary revascularization	NA	NA	2.0	0.7	NA	NA	NA	NA	6.6	2.2	2.6	0.1
CABG	5.8	0.1	0.7	0.9	NA	NA	0.9	0.4	5.6	0.1	NA	NA
PCI	3.1	0.2	2.0	1.3	NA	NA	2.9	0.3	3.3	1.9	NA	NA
Carotid intervention	2.3	0.1	NA	NA	NA	NA	NA	NA	4.3	NA	0.4	0.4
Atrial fibrillation	13.5	0.1	9.6	0.6	7.1	0.7	11.0	0.6	17.3	2.3	4.9	0.2
Microvascular disease	NA	NA	16.2	0.9	28.8	2.2	4.1	1.8	24.3	0	1.0	1.0

**CKD†**	15.5	1.0	16.3	0.4	15.3	1.2	13.2	1.4	12.7	0	NA	NA
**Frailty(yes)‡**	26.4	0.2	22.2	1.6	0.7	0	22.7	0.3	13.8	7.7	26.2	18.9
**Migrant**	NA	NA	NA	NA	NA	NA	NA	NA	NA	NA	8.0	0.6
**Smoker (yes)**	6.5	0.6	NA	NA	NA	NA	NA	NA	3.6	0.1	4.3	2.0
**HbA1c (mean)**	NA	NA	NA	NA	NA	NA	NA	NA	19.1	11.0	7.3	3.1
**HbA1c >7%**	NA	NA	NA	NA	NA	NA	NA	NA	39.1	1.5	10.15	1.8
**BMI (mean)**	NA	NA	NA	NA	NA	NA	NA	NA	42.4	10.9	14.55	2.35
**BMI≥30**	NA	NA	NA	NA	NA	NA	NA	NA	44.6	1.3	14.8	4.6
**Total cholesterol**	NA	NA	NA	NA	NA	NA	NA	NA	14.8	0	5.3	2.1
**eGFR (mean)**	NA	NA	NA	NA	NA	NA	NA	NA	24.2	10.9	27.1	5.0
**eGFR <60**	NA	NA	NA	NA	NA	NA	NA	NA	46.8	0.1	25.2	5.7
**Nephropathy**	3.6	0.5	NA	NA	NA	NA	NA	NA	0.1	0.3	NA	NA
**Peripheral neuropathy**	6.1	0.1	NA	NA	NA	NA	NA	NA	6.4	0.8	5.1	0.5
**Retinopathy**	7.4	0.1	NA	NA	NA	NA	NA	NA	23.8	0.1	0.2	0.5
**Hypertension**	9.4	0	NA	NA	NA	NA	NA	NA	1.0	2.1	NA	NA
**Microalbuminuria**	NA	NA	NA	NA	NA	NA	NA	NA	NA	NA	0.4	0.4
**Weight-loss drugs**	8.2	0.8	3.2	0.4	2.1	0	6.3	0.4	7.1	0.9	1.2	0
**Bariatric surgery**	NA	NA	NA	NA	NA	NA	1.7	0.2	4.5	2.9	2.1	0
**Time from start**	NA	NA	NA	NA	NA	NA	NA	NA	90.9	0	55.2	0.6
**T2D duration**	NA	NA	67.4	1.7	9.4	15.5	77.9	1.6	43.8	1.7	8.9	0.4
**Glucose-lowering therapies**												

**Metformin**	86.2	1.7	61.3	7.7	80.4	6.3	76.5	6.0	96.0	2.7	21.7	0.5
**Sulfonylurea**	40.0	0.3	44.2	0.5	31.1	2.2	76.5	2.0	52.1	1.1	5.5	0.6
**DPP-4 inhibitor**	57.1	2.3	43.1	1.0	37.5	1.6	25.6	0.1	79.9	1.4	8.9	0.9
**TZD**	22.9	1.8	13.0	0.7	NA	NA	17.9	2.1	18.0	0.1	NA	NA
**GLP-1 RA**	54.2	8.1	44.3	2.3	60.3	4.5	59.8	4.8	61.3	0.6	13.8	0.6
**Insulin**	32.3	1.1	1.1	3.2	23.3	1.5	34.6	1.8	49.8	2.3	10.2	0.4
**Cardiovascular therapies**												
**Antihypertensive therapy§**	11.0	0.2	13.2	0.3	27.6	0.8	14.2	0.1	5.0	0	6.9	0.3
**Loop diuretics**	11.8	0.2	11.8	0.9	10.7	1.2	7.1	0.7	7.5	1.1	3.1	0.3
**Thiazide diuretics**	5.1	0	2.7	0.5	2.9	0	1.7	0.8	1.7	0.1	1.9	0.3
**β-blocker**	6.7	0.3	0.5	1.3	0.9	1.6	2.8	0.5	9.4	0.2	1.0	1.2
**Statin therapy**	17.8	0.1	21.4	0.3	42.3	4.8	25.2	0.2	21.1	2.2	7.4	0.2
**Aspirin**	NA	NA	7.2	0.4	13.6	1.7	5.6	0.4	2.1	2.5	0.2	0.8
**PY12 inhibitors**	NA	NA	0.6	0.4	3.5	0.7	1.0	NA	NA	0.8	1.9	0.5
**Antiplatelets**	6.0	0.2	NA	NA	NA	NA	NA	NA	0.2	2.1	NA	NA
**Warfarin**	NA	NA	10.1	0.8	8.3	1.2	8.1	0.7	13.4	2.5	1.6	0.7
**Anticoagulants**	10.6	0.3	NA	NA	NA	NA	NA	NA	14.2	1.8	NA	NA
**Aldosterone antagonists**	3.2	0	2.0	0.4	2.2	1.1	0.8	0.5	8.2	1.0	3.4	0.7

*Defined as myocardial infarction, unstable angina, stroke, heart failure, transient ischemic attack, coronary revascularization (coronary artery bypass grafting or percutaneous coronary intervention) or occlusive peripheral artery disease on or ever prior to start ; †CKD in UK CPRD/THIN and US Truven MarketScan does not include end stage renal disease; ‡Frailty in UK CPRD/THIN is defined as ≥1 hospitalization within 1 year prior to index date;§Includes angiotensin converting enzyme inhibitors, angiotensin receptor blockers, Ca^{2+} channel blockers β-blockers, thiazides; BMI=body mass index; CABG=Coronary artery bypass

grafting; CKD=chronic kidney disease; CPRD, Clinical Practice Research Datalink; CV=cardiovascular; DPP-4=dipeptidyl peptidase-4; DPV=Diabetes Patientenverlaufsdokumentation; eGFR=estimated Glomerular Filtration Rate; GLP-1RA= Glucagon-like peptide-1 receptor agonist; MI=myocardial infarction; OAD=oral antidiabetic drugs; PAD=peripheral arterial disease; PCI=percutaneous coronary intervention; T2D=Type 2 diabetes; THIN=The Health Improvement Network; TZD=thiazolidinedione; UK=United Kingdom; US=United States; NA=not applicable (some characteristics were not available in every country)

Table S8. Composition of SGLT-2 inhibitor class in propensity matched cohorts

	SGLT-2 inhibitor patients, N	Proportions by agent		
		Canagliflozin, %	Dapagliflozin, %	Empagliflozin, %
**Hospitalization for heart failure analysis**				
**US Truven MarketScan**	116,899	70	22	8
**Norway national registers**	12,525	0	83	17
**Denmark national registers**	9234	4	79	17
**Sweden national registers**	9189	0	83	17
**UK CPRD/THIN**	5231	2	86	12
**Germany DPV**	1450	2	64	33
**Total**	**154,528**	**53**	**37**	**10**
**All-cause death analysis, and composite (HHF or all-cause death) analysis**				
**US Truven MarketScan**	71,632	69	22	8
**Norway national registers**	12,525	0	83	17
**Denmark national registers**	9234	4	79	17
**Sweden national registers**	9189	0	83	17
**UK CPRD/THIN**	5231	2	86	12
**Total**	**107,811**	**47**	**43**	**11**

Table S9. Index glucose-lowering medication classes for patients in the other GLD group: all countries combined (HHF analysis)

Variable	Other GLD (N=154,528)
**Index medication*, n (%)**	
**MET**	17,899 (11.6)
**SU**	26,203 (17.0)
**DPP-4 inhibitor**	26,957 (17.4)
**TZD**	7496 (4.9)
**GLP-1 receptor agonist**	21,199 (13.7)
**Insulin**	51,838 (33.5)
**Acarbose**	708 (0.5)
**Amylin analog**	132 (0.1)
**Meglitinides**	2096 (1.4)

*In cases of ≥2 medications, one was randomly selected; DPP-4=dipeptidyl peptidase-4; GLD=glucose-lowering drug; GLP-1= Glucagon-like peptide-1; HHF=hospitalization for heart failure; MET=metformin; SU=sulfonylurea; TZD= thiazolidinedione

Table S10. Index glucose-lowering medication classes for patients in the other GLD group: all countries combined (all-cause death analysis and HHF or all-cause death analysis)

Variable	Other GLD (N=107,811)
**Index medication*, n (%)**	
**MET**	12,134 (11.3)
**SU**	18,203 (16.9)
**DPP-4 inhibitor**	19,665 (18.2)
**TZD**	4731 (4.4)
**GLP-1 receptor agonist**	14,743 (13.7)
**Insulin**	36,432 (33.8)
**Acarbose**	424 (0.4)
**Amylin analog**	78 (0.1)
**Meglitinides**	1401 (1.3)

*In cases of ≥2 medications, one was randomly selected; DPP-4=dipeptidyl peptidase-4; GLD=glucose-lowering drug; GLP-1= Glucagon-like peptide-1; HHF=hospitalization for heart failure; MET=metformin; SU=sulfonylurea; TZD= thiazolidinedione

Table S11: Number of patients, person-years at risk (on treatment), events and events/100 person-years (incidence rates) for each of the endpoints

Country/database	Patients, N	Person-years	Events, n	Events / 100 person-years
**Hospitalization for heart failure**				
**US Truven MarketScan**	233,798	125,904	298	0.24
**Norway national registers**	25,050	25,166	278	1.10
**Denmark national registers**	18,468	17,159	167	0.97
**Sweden national registers**	18,378	13,688	191	1.40
**UK CPRD/THIN**	10,462	6833	16	0.23
**Germany DPV**	2900	1414	11	0.78
**Total**	309,056	190,164	961	0.51
**All-cause death**				
**US Truven MarketScan**	143,264	80,556	250	0.31
**Norway national registers**	25,050	25,368	364	1.43
**Denmark national registers**	18,468	17,267	323	1.87
**Sweden national registers**	18,378	23,946	317	1.32
**UK CPRD/THIN**	10,462	6853	80	1.17
**Total**	215,622	153,990	1334	0.87
**Composite of hospitalization for heart failure or all-cause death**				
**US Truven MarketScan**	143,264	80,496	424	0.53
**Norway national registers**	25,050	25,166	622	2.47
**Denmark national registers**	18,468	17,159	477	2.78
**Sweden national registers**	18,378	13,688	364	2.66
**UK CPRD/THIN**	10,462	6833	96	1.40
**Total**	215,622	143,342	1983	1.38

CPRD= Clinical Practice Research Datalink; DPV= Diabetes Patientenverlaufsdokumentation; SGLT-2=sodium-glucose cotransporter-2; THIN=The Health Improvement Network; UK=United Kingdom; US=United States

Table S12. Pooled event rates by treatment groups

	SGLT-2i			oGLD		
	Events	Person-years	Events/100 person-years	Events	Person-years	Events/100 person-years
**Hospitalization for heart failure**	367	100,952	0.36	594	89,212	0.67
**All-cause death**	412	79,888	0.52	922	74,102	1.24
**Hospitalization for heart failure and all-cause death**	667	74,665	0.89	1316	68,677	1.92

Table S13. Mean on treatment follow-up time (in days) by country and endpoint

	HHF		ACD		HHF/ACD	
	SGLT-2i	oGLD	SGLT-2i	oGLD	SGLT-2i	oGLD
US MarketScan/Truven	217	176	225	186	225	186
UK-CPRD/THIN	225	252	226	253	225	252
Sweden	261	283	463	489	261	283
Norway	364	370	366	374	364	370
Denmark	328	351	330	353	328	351
Germany	236	124				
Total	239	211	271	251	253	233

ACD=all-cause death; HHF=hospitalization for heart failure; oGLD=other glucose-lowering drug; SGLT-2i=sodium-glucose cotransporter-2

Table S14. Sensitivity analyses examining association between treatment with SGLT-2i vs. oGLD and outcomes of HHF in Sweden, among patients with both in- and outpatient hospital visits with primary diagnosis of heart failure, and those with only inpatient hospital visits with the primary diagnosis of heart failure.

	oGLD<br>Events, n (events/100PY)	SGLT-2i<br>Events, n (events/100PY)	Hazard ratio	95% CI	P-value
**On treatment, both in- and outpatient**	122 (17.1)	69 (10.5)	0.61	0.45-0.82	0.001
**On treatment, only inpatient**	70 (9.8)	30 (4.6)	0.46	0.30-0.71	<0.001
**ITT, both in- and outpatient**	150 (17.1)	101 (12.3)	0.72	0.56-0.93	0.012
**ITT, only inpatient**	89 (10.1)	49 (5.9)	0.59	0.42-0.84	0.003

CI=confidence interval; HHF=hospitalization for heart failure; oGLD=other glucose-lowering drug; PY=person-years; SGLT-2i=sodium-glucose cotransporter-2.

SUPPLEMENTARY FIGURES

Figure S1: Patient selection flow-charts for each country

A. US Truven MarketScan

Step	SGLT2i Patients	Other GLD Patients
New users of glucose-lowering drugs*	179,581	5,492,777
↓		
Excluded for not meeting the study eligibility criteria	55,933	4,780,351
↓		
Patients used in propensity score 1:1 matching	123,648	712,426
↓		
Excluded because match was not available	6749	595,527
↓		
Final cohort (after 1:1 match)	116,899	116,899
↓ For HHF analysis	116,899	116,899
↓ For all-cause death analysis; For HHF or all-cause death analysis	71,632	71,632

*Any users of glucose-lowering drugs

B. Norway National Registers

Step	SGLT2i Patients	Other GLD Patients
New users of glucose-lowering drugs	14,438	96,947
↓		
Excluded for not meeting the study eligibility criteria*	N/A	N/A
↓		
Patients used in propensity score 1:1 matching	14,438	96,947
↓		
Excluded because match was not available	1913	84,422
↓		
Final cohort (after 1:1 match)	12,525	12,525
↓ For HHF analysis	12,525	12,525
↓ For all-cause death analysis; For HHF or all-cause death analysis	12,525	12,525

*Inclusion/exclusion criteria were already applied prior to the first step

C. Denmark National Registers

Step	SGLT2i Patients	Other GLD Patients
New users of glucose-lowering drugs	9522	119,137
↓		
Excluded for not meeting the study eligibility criteria*	N/A	N/A
↓		
Patients used in propensity score 1:1 matching	9522	119,137
↓		
Excluded because match was not available	288	109,903
↓		
Final cohort (after 1:1 match)	9234	9234
↓ For HHF analysis	9234	9234
↓ For all-cause death analysis; For HHF or all-cause death analysis	9234	9234

*Inclusion/exclusion criteria were already applied prior to the first step

D. Sweden National Registers

Step	SGLT2i Patients	Other GLD Patients
New users of glucose-lowering drugs	9337	200,284
↓		
Excluded for not meeting the study eligibility criteria*	N/A	N/A
↓		
Patients used in propensity score 1:1 matching	9337	200,284
↓		
Excluded because match was not available	148	191,095
↓		
Final cohort (after 1:1 match)	9189	9189
↓ For HHF analysis	9189	9189
↓ For all-cause death analysis; For HHF or all-cause death analysis	9189	9189

*Inclusion/exclusion criteria were already applied prior to the first step

E. UK CPRD/THIN

Step	SGLT2i Patients	Other GLD Patients
New users of glucose-lowering drugs	7556	73,436
↓		
Excluded for not meeting the study eligibility criteria*	N/A	N/A
↓		
Patients used in the propensity score model	7556	73,436
↓		
Patients used in propensity score 1:1 matching**	6298	65007
↓		
Excluded because match was not available	1067	59776
↓		
Final cohort (after 1:1 match)	5231	5231
↓ For HHF analysis	5231	5231
↓ For all-cause death analysis; For HHF or all-cause death analysis	5231	5231

*Inclusion/exclusion criteria were already applied prior to the first step; **Matching between cohorts was conducted within THIN patients and within CPRD patients with Hospital Episode Statistics (HES) and mortality data linkage (not overlapping with THIN)

F. Germany DPV

Step	SGLT2i Patients	Other GLD Patients
New users of glucose-lowering drugs	1532	23,991
↓		
Excluded for not meeting the study eligibility criteria*	N/A	N/A
↓		
Patients used in propensity score 1:1 matching	1532	23,991
↓		
Excluded because match was not available	87	22,546
↓		
Final cohort (after 1:1 match)	1450	1450
↓		
For HHF analysis	1450	1450

*Inclusion/exclusion criteria were already applied prior to the first step

CPRD=Clinical Practice Research Datalink; DPV= Diabetes Patientenverlaufsdokumentation; GLD=glucose-lowering drugs; HHF=hospitalization for heart failure; SGLT-2i=sodium-glucose cotransporter-2 inhibitors; THIN=The Health Improvement Network; UK=United Kingdom; US=United States

Figure S2: Propensity score distribution by country pre-match

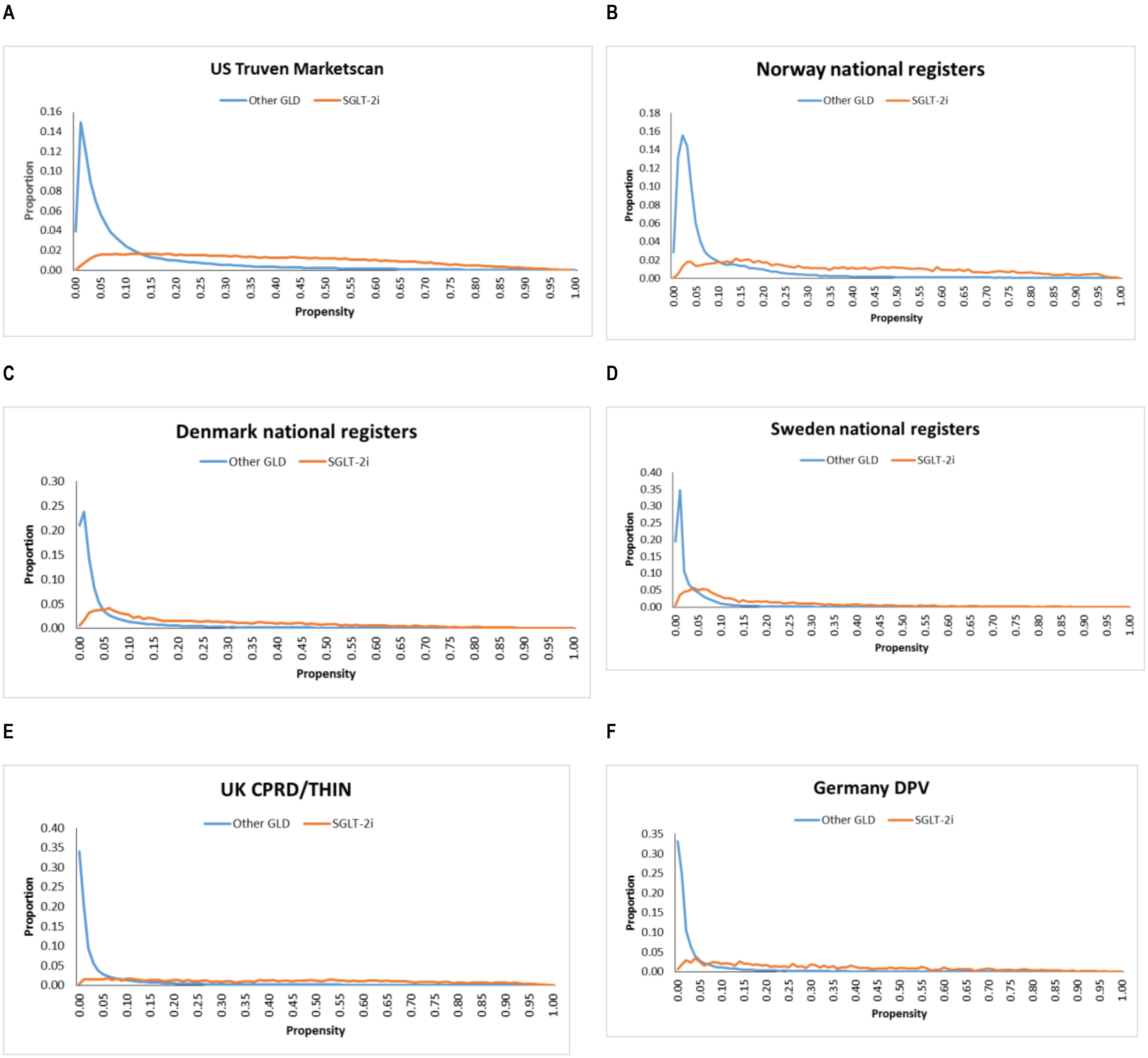

CPRD=Clinical Practice Research Datalink; DPV=Diabetes Patientenverlaufsdokumentation; SGLT-2=sodium-glucose cotransporter-2; THIN=The Health Improvement Network; UK=United Kingdom; US=United States

Figure S3: Propensity score distribution by country post-match

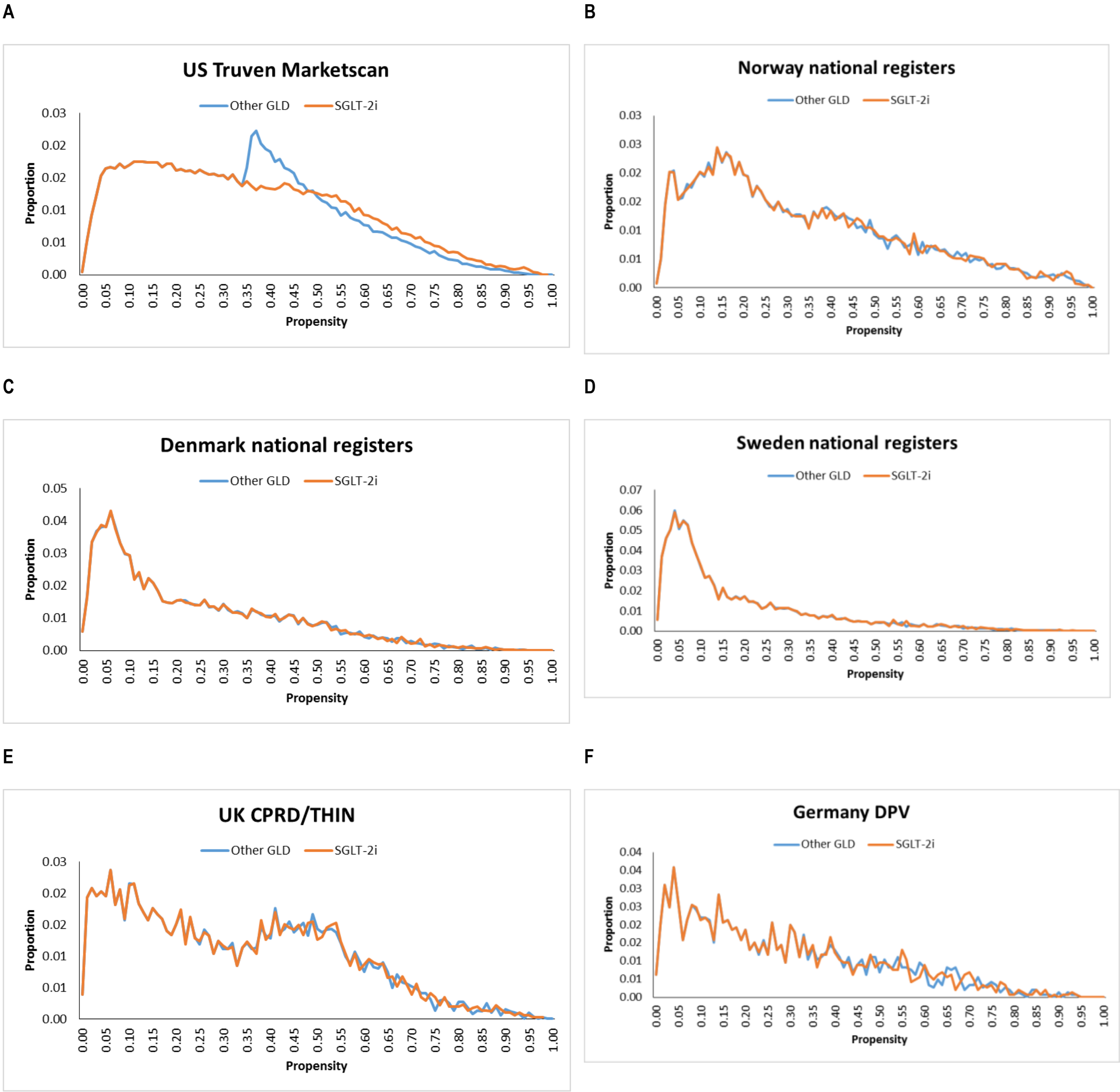

CPRD=Clinical Practice Research Datalink; DPV=Diabetes Patientenverlaufsdokumentation; SGLT-2=sodium-glucose cotransporter-2; THIN=The Health Improvement Network; UK=United Kingdom; US=United States

Figure S4: Standardized difference between SGLT-2 inhibitor and other GLD treatment groups by country

A. US Truven MarketScan

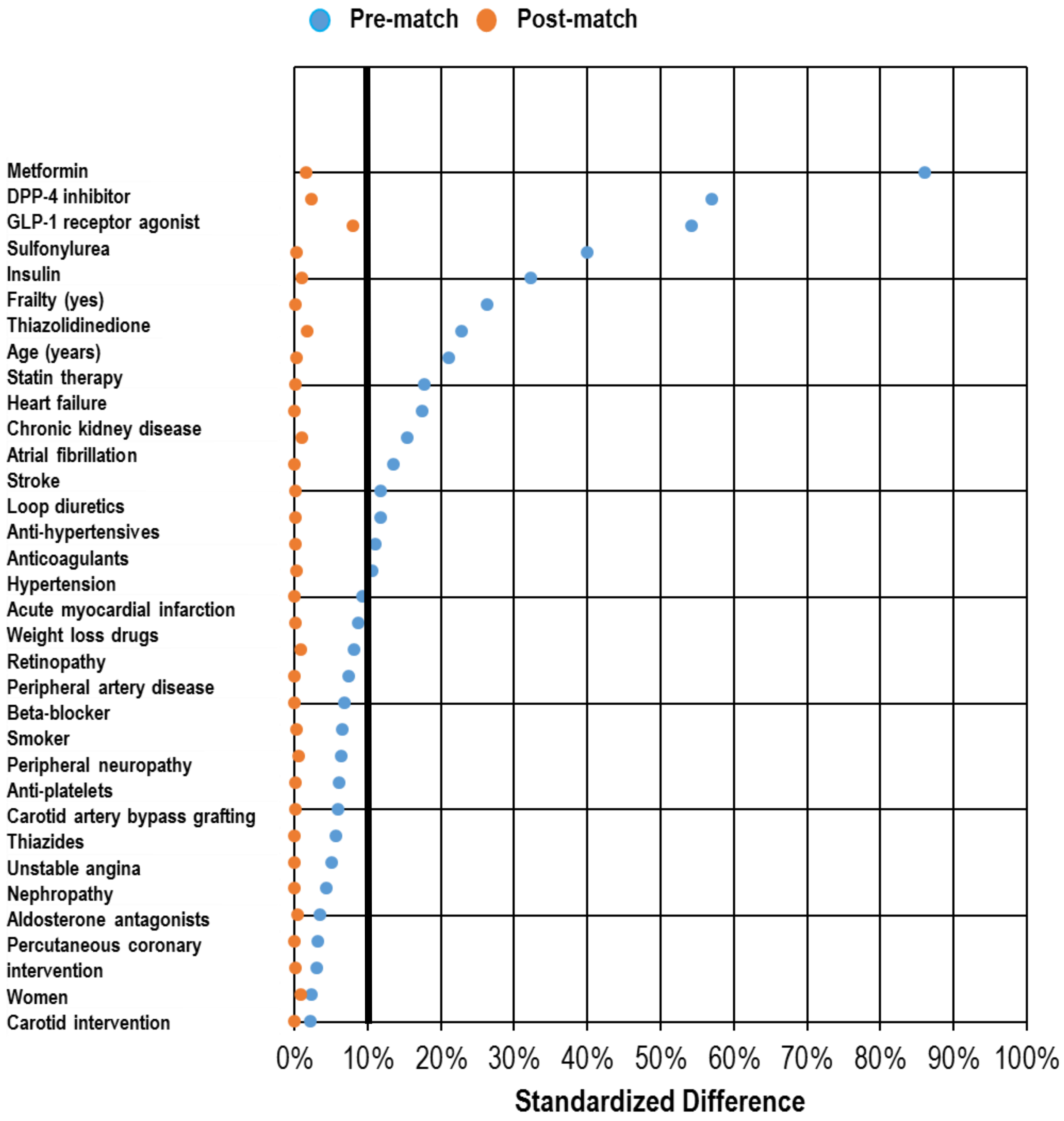

B. Norway National Registers

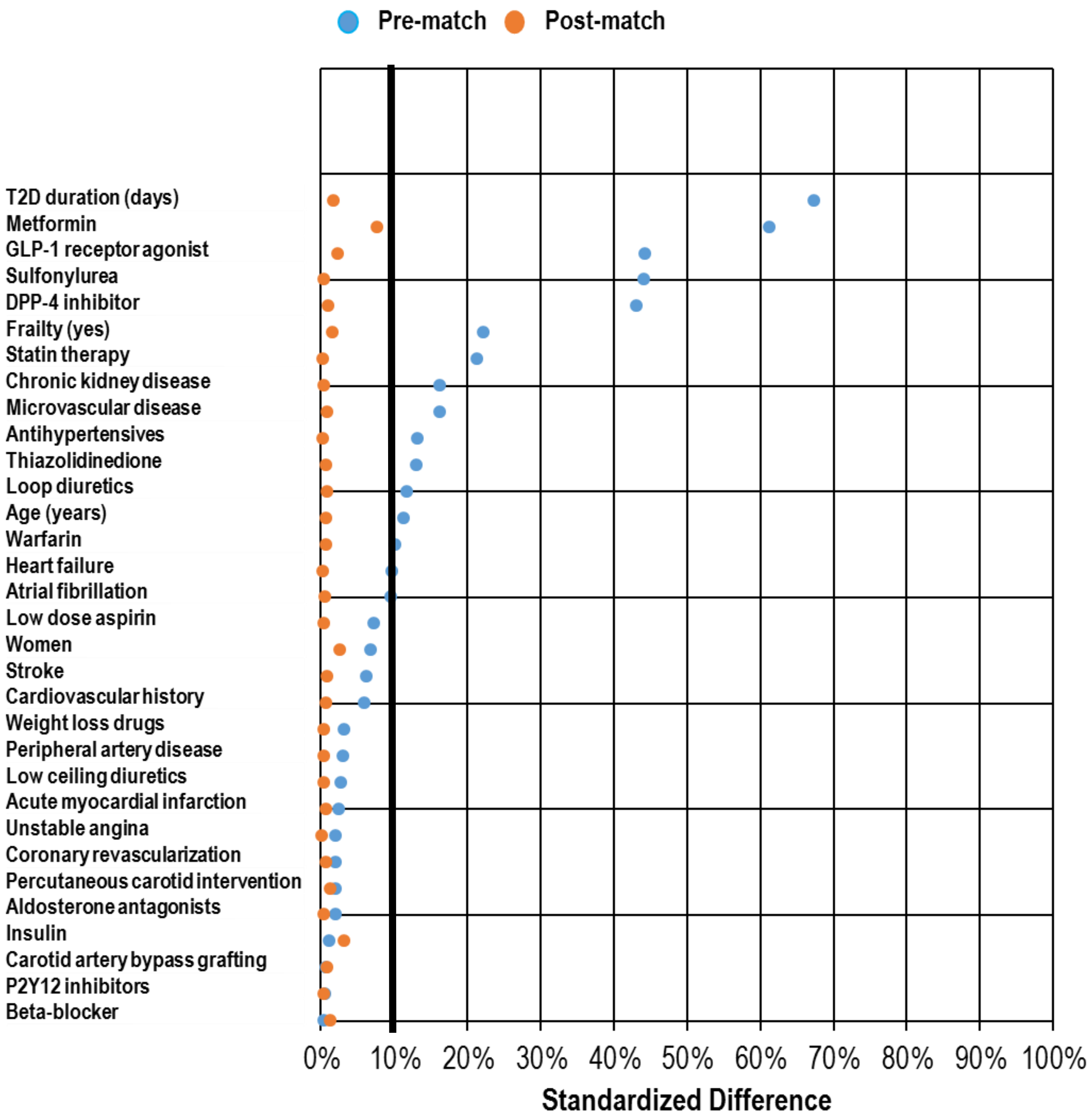

C. Denmark National Registers

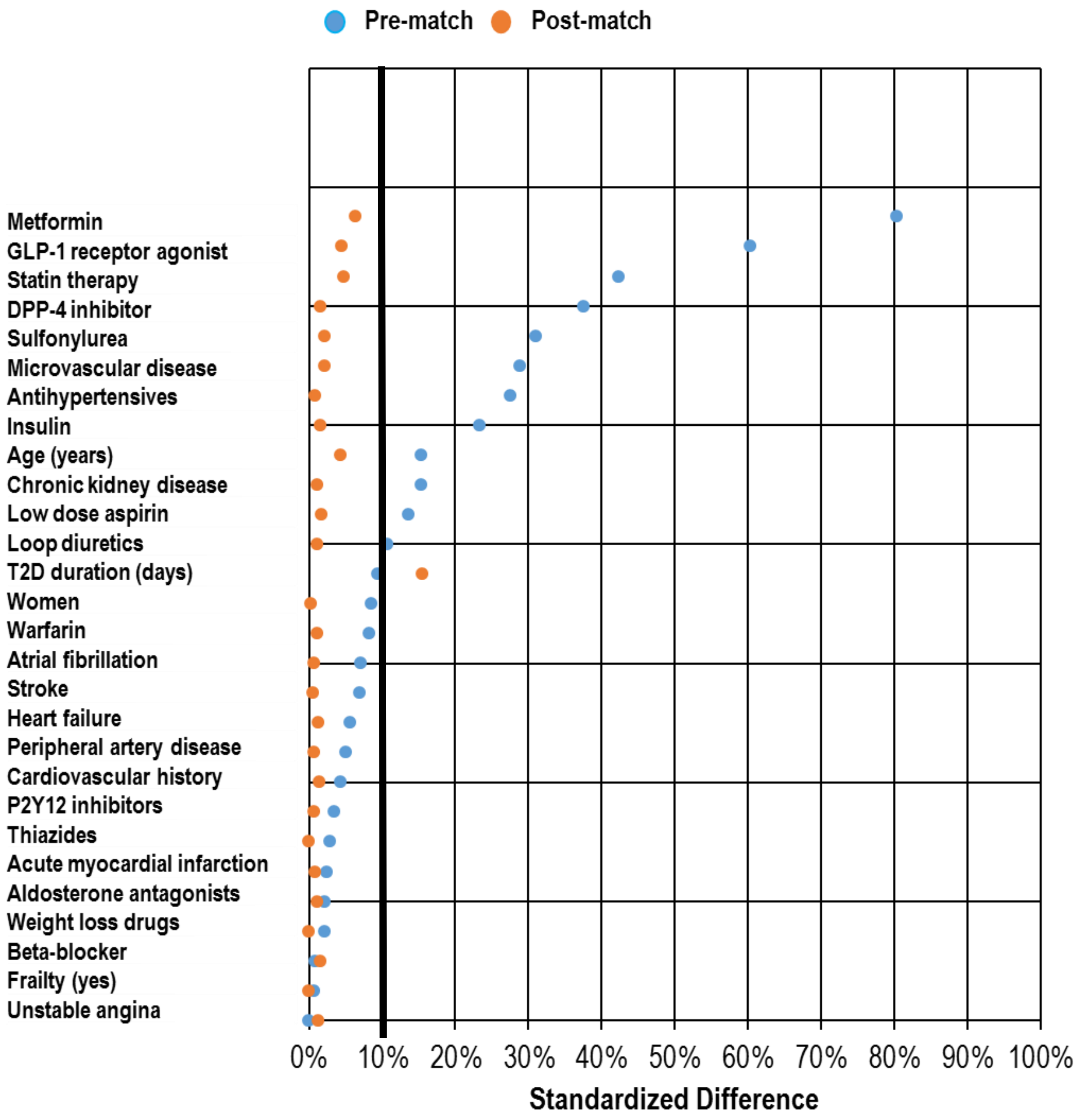

D. Sweden National Registers

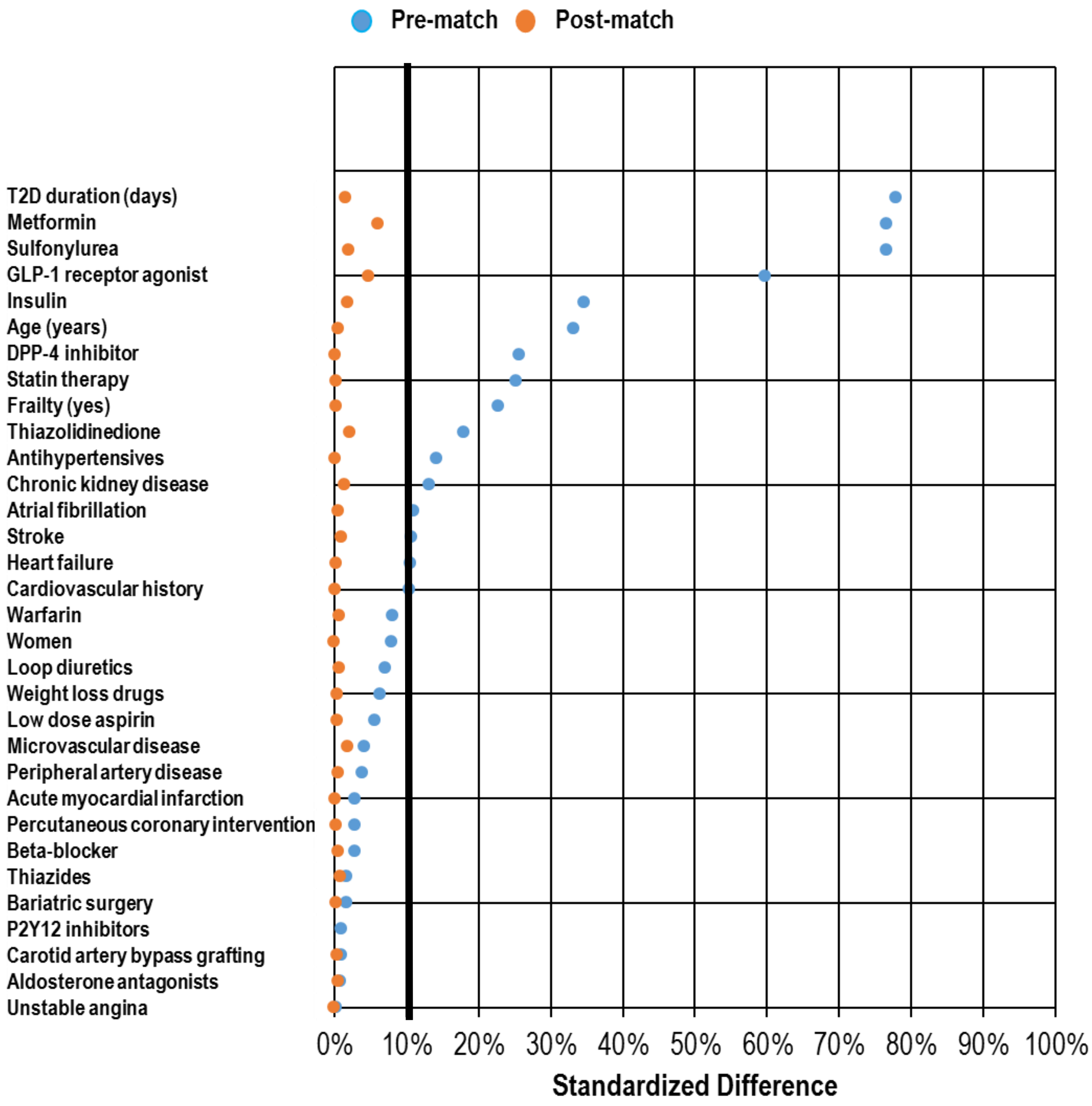

E. UK CPRD/THIN

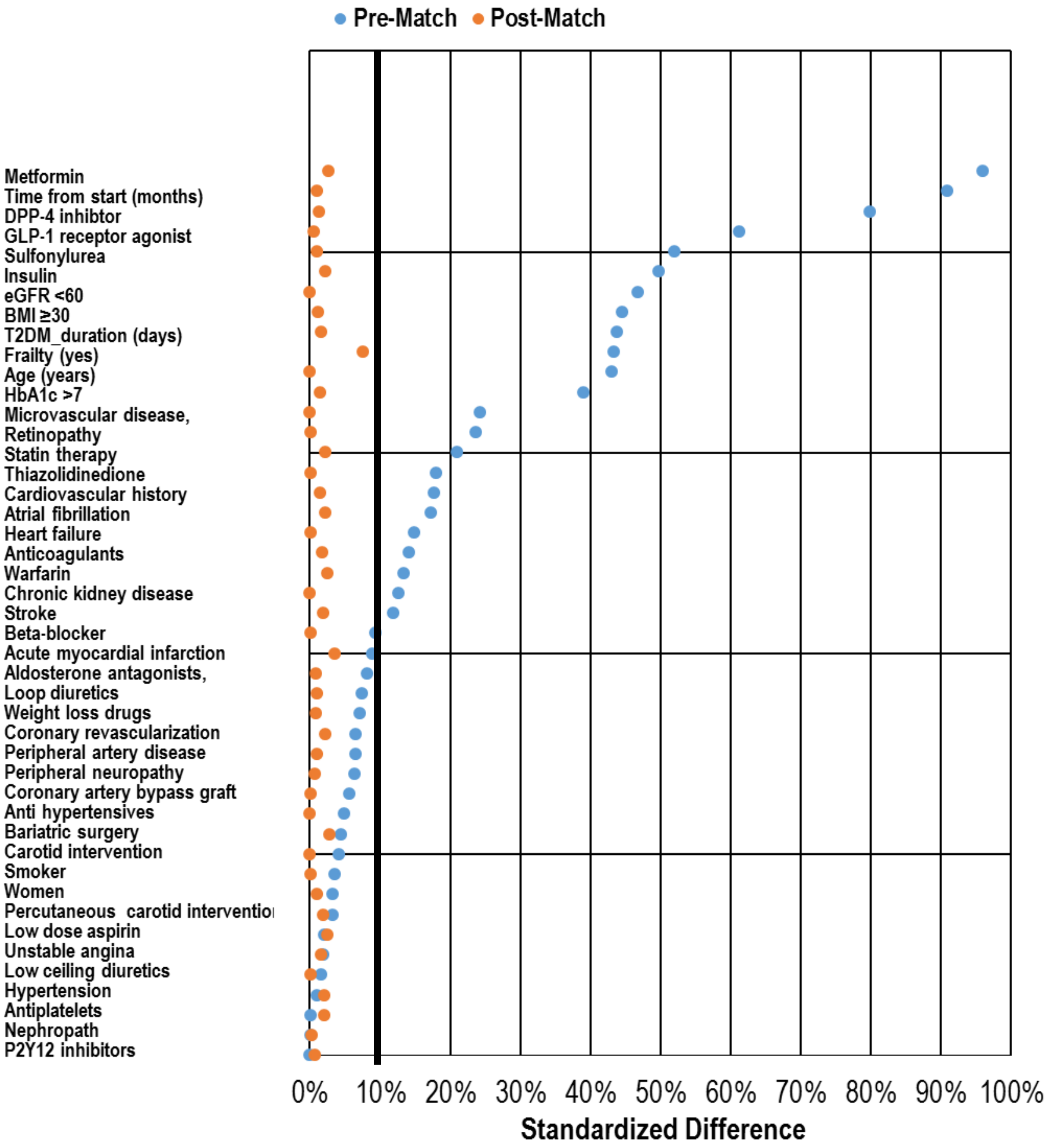

Pre-Match
Post-Match
Metformin
Time from start (months)
DPP-4 inhibtor
GLP-1 receptor agonist
Sulfonylurea
Insulin
eGFR <60
BMI ≥30
T2DM_duration (days)
Frailty (yes)
Age (years)
HbA1c >7
Microvascular disease,
Retinopathy
Statin therapy
Thiazolidinedione
Cardiovascular history
Atrial fibrillation
Heart failure
Anticoagulants
Warfarin
Chronic kidney disease
Stroke
Beta-blocker
Acute myocardial infarction
Aldosterone antagonists,
Loop diuretics
Weight loss drugs
Coronary revascularization
Peripheral artery disease
Peripheral neuropathy
Coronary artery bypass graft
Anti hypertensives
Bariatric surgery
Carotid intervention
Smoker
Women
Percutaneous carotid interventio
Low dose aspirin
Unstable angina
Low ceiling diuretics
Hypertension
Antiplatelets
Nephropath
P2Y12 inhibitors
0%
10%
20%
30%
40%
50%
60%
70%
80%
90%
100%
Standardized Difference

F. Germany DPV

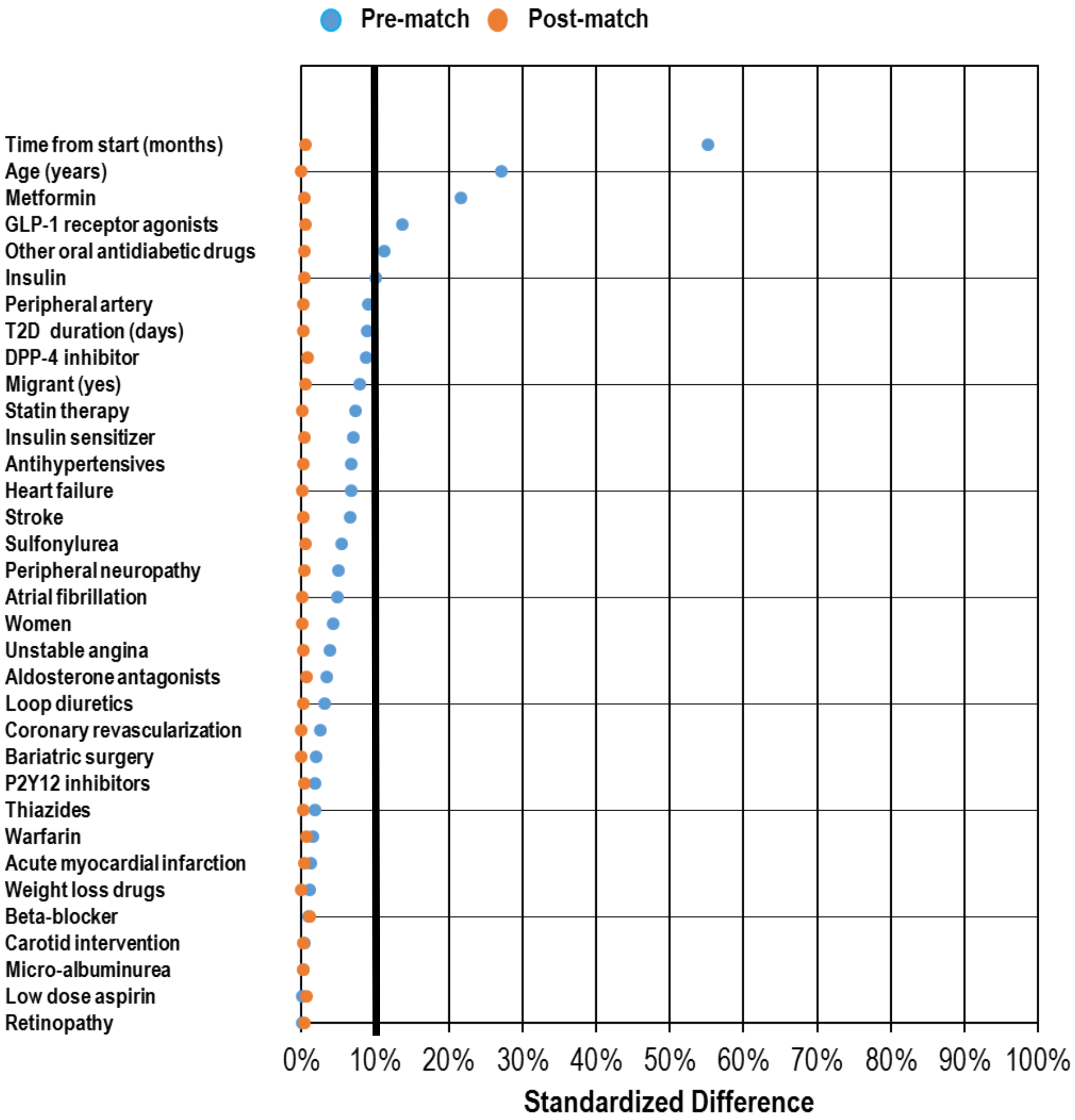

DPP-4=dipeptidyl peptidase-4; eGFR=estimated Glomerular Filtration Rate; GLP-1= Glucagon-like peptide-1; oGLD=other glucose-lowering drug; SGLT-2i=sodium-glucose cotransporter -2 inhibitor; T2D=Type 2 diabetes

Figure S5: Stepwise sensitivity analysis (sequentially removing comparators): Outcome of hospitalization for heart failure

A. On treatment, adjusted* – TZD removed

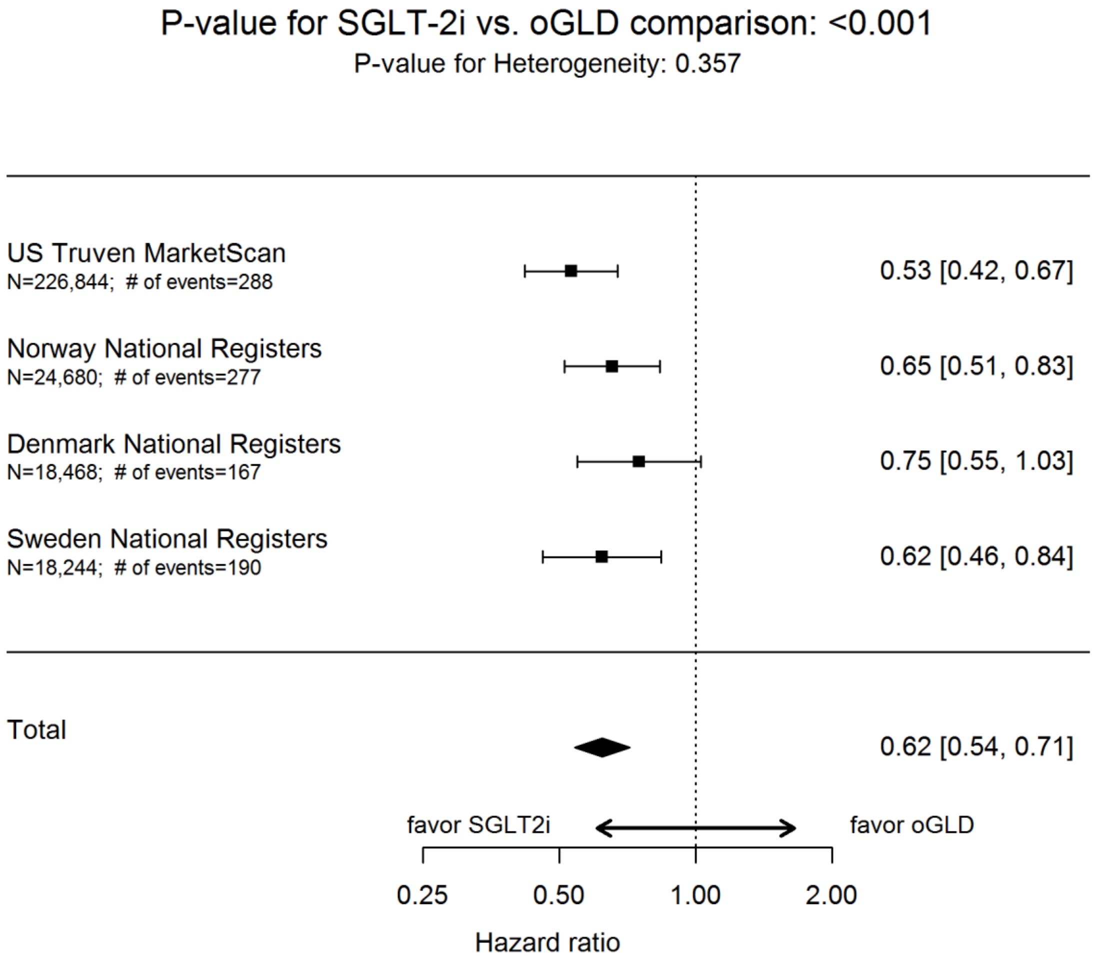

*Adjusted for history of heart failure, age, gender, frailty, history of myocardial infarction, history of atrial fibrillation, hypertension, obesity / body mass index, duration of diabetes, ACE inhibitor or ARB use; β-blocker or α-blocker use, Ca^{+}-channel blocker use, loop diuretic use, thiazide diuretic use; TZD=thiazolidinedione

B. On treatment, adjusted* – TZD and insulin removed

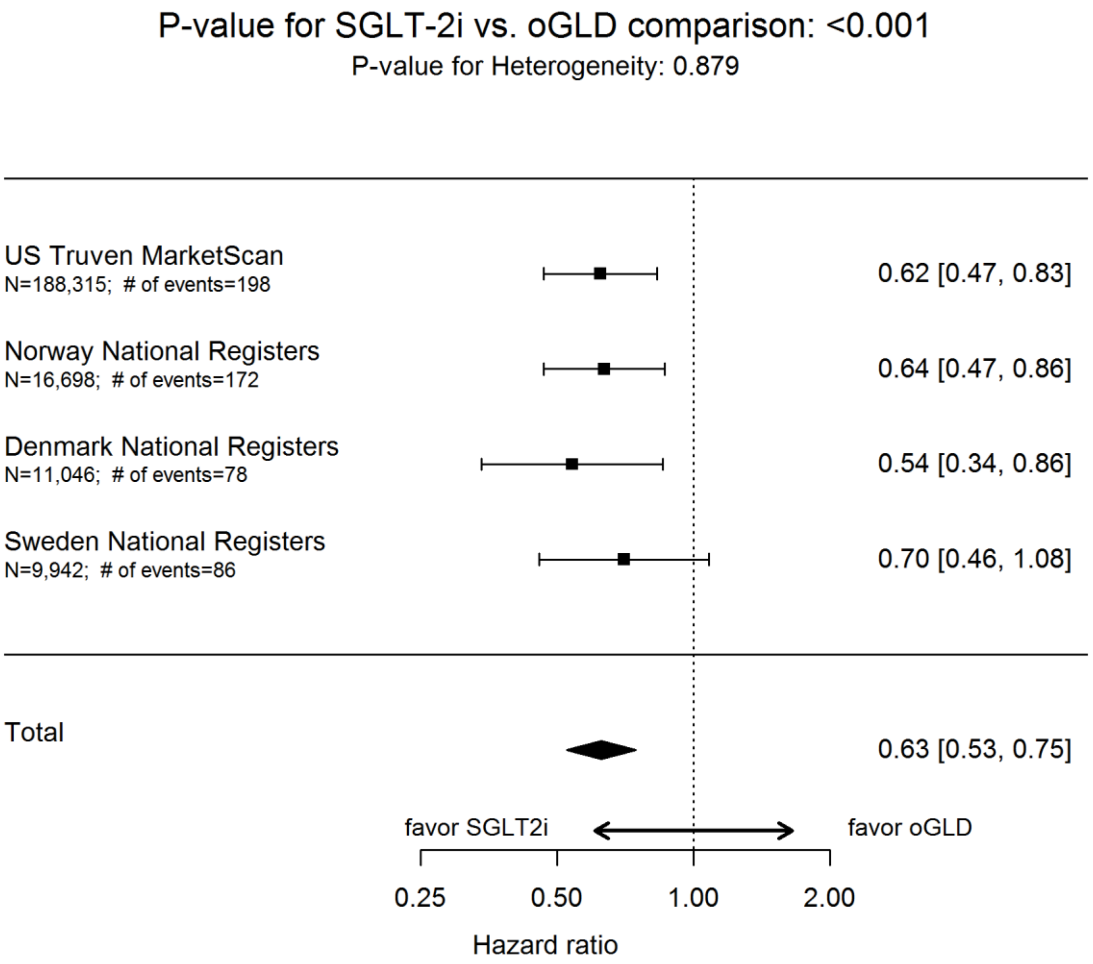

*Adjusted for history of heart failure, age, gender, frailty, history of myocardial infarction, history of atrial fibrillation, hypertension, obesity / body mass index, duration of diabetes, ACE inhibitor or ARB use; β-blocker or α-blocker use, Ca^+-channel blocker use, loop diuretic use, thiazide diuretic use; TZD= thiazolidinedione

C. On treatment, adjusted* – TZD, insulin and SU removed

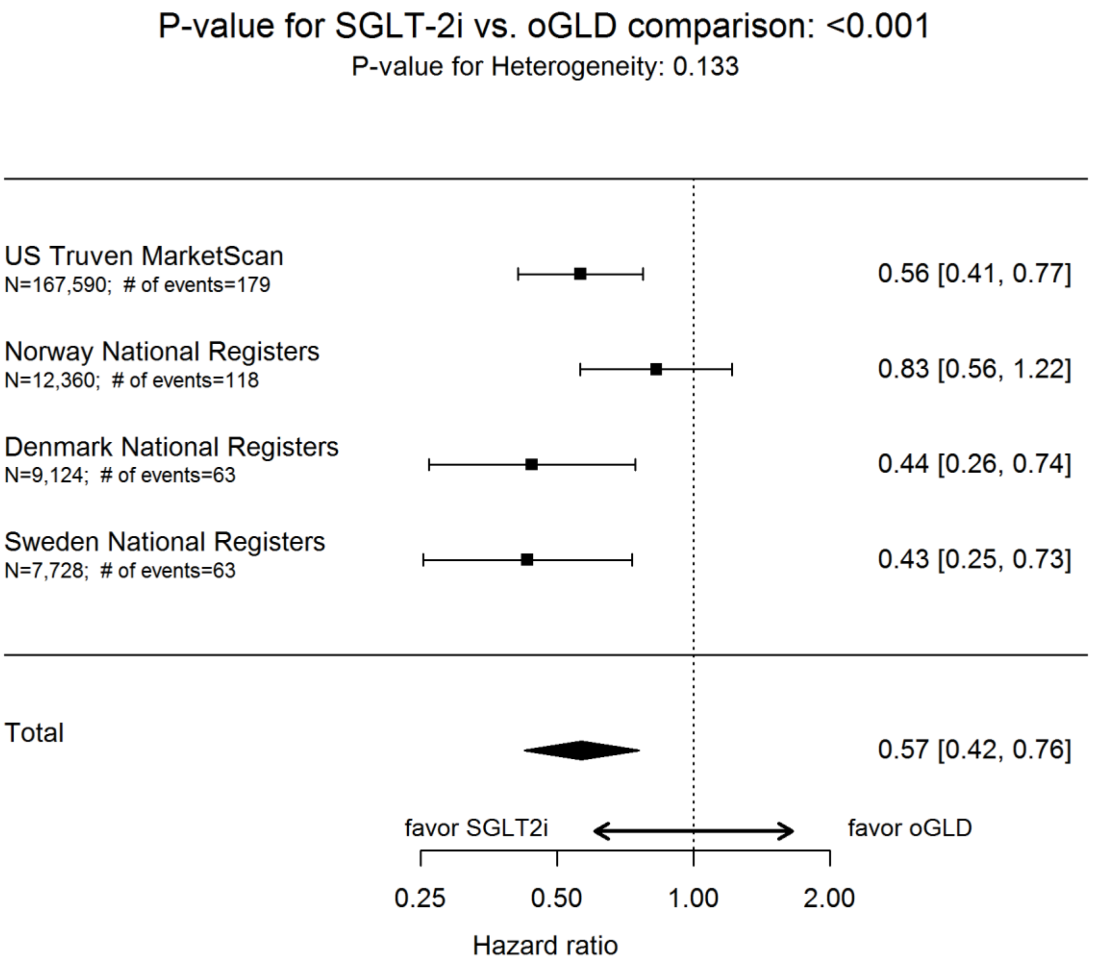

*Adjusted for history of heart failure, age, gender, frailty, history of myocardial infarction, history of atrial fibrillation, hypertension, obesity / body mass index, duration of diabetes, ACE inhibitor or ARB use; β-blocker or α-blocker use, Ca^{+}-channel blocker use, loop diuretic use, thiazide diuretic use; SU=sulfonylurea; TZD= thiazolidinedione

Figure S6: Outcome of hospitalization for heart failure excluding patients with baseline GLP-1 RAs, on treatment, adjusted*

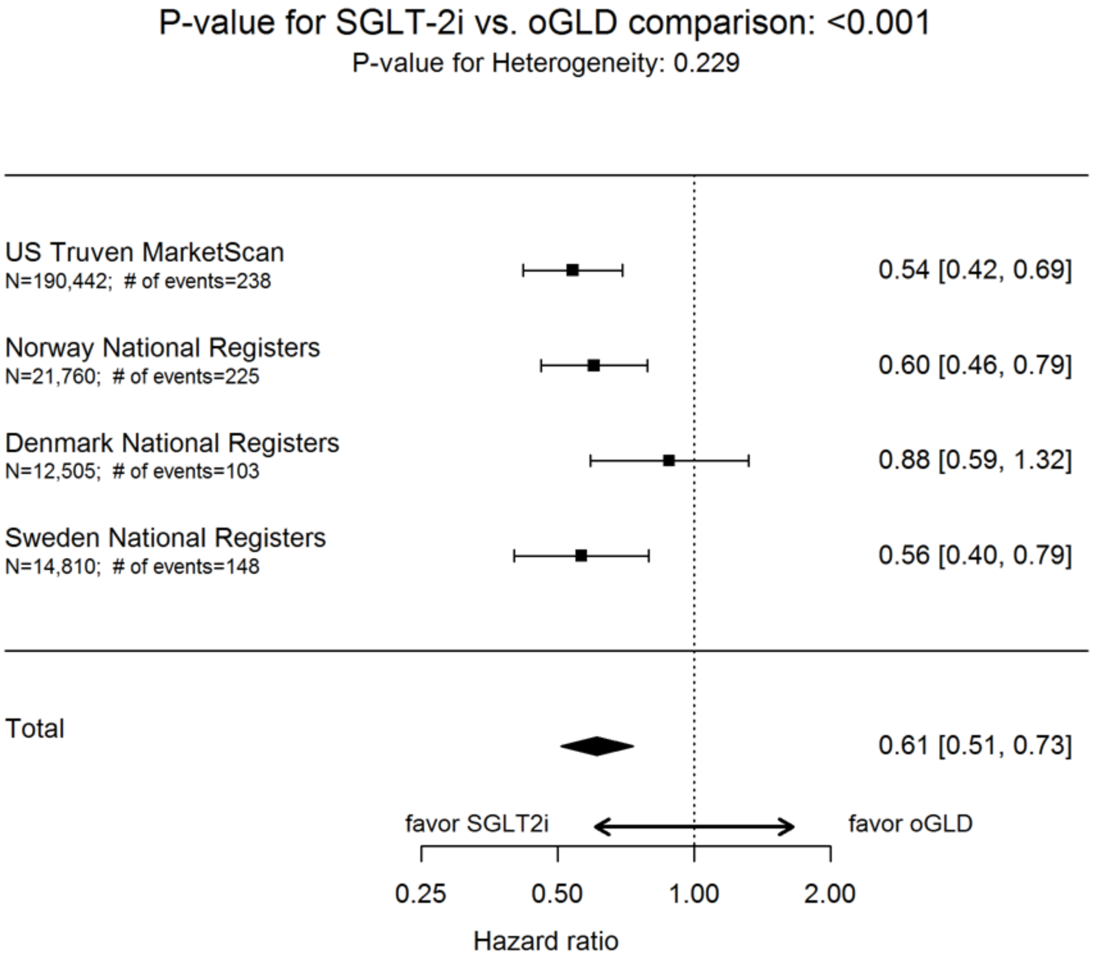

*Adjusted for history of heart failure, age, gender, frailty, history of myocardial infarction, history of atrial fibrillation, hypertension, obesity / body mass index, duration of diabetes, ACE inhibitor or ARB use; β-blocker or α-blocker use, Ca^{+}-channel blocker use, loop diuretic use, thiazide diuretic use; SU=sulfonylurea; TZD= thiazolidinedione

Data from Germany and UK not included as they contributed too few HHF events after removal of GLP1-RA class

Figure S7: Outcomes for hospitalization for heart failure, all-cause death, and hospitalization for heart failure or all-cause death for the SGLT-2 inhibitor versus other GLD treatment groups

A. US only

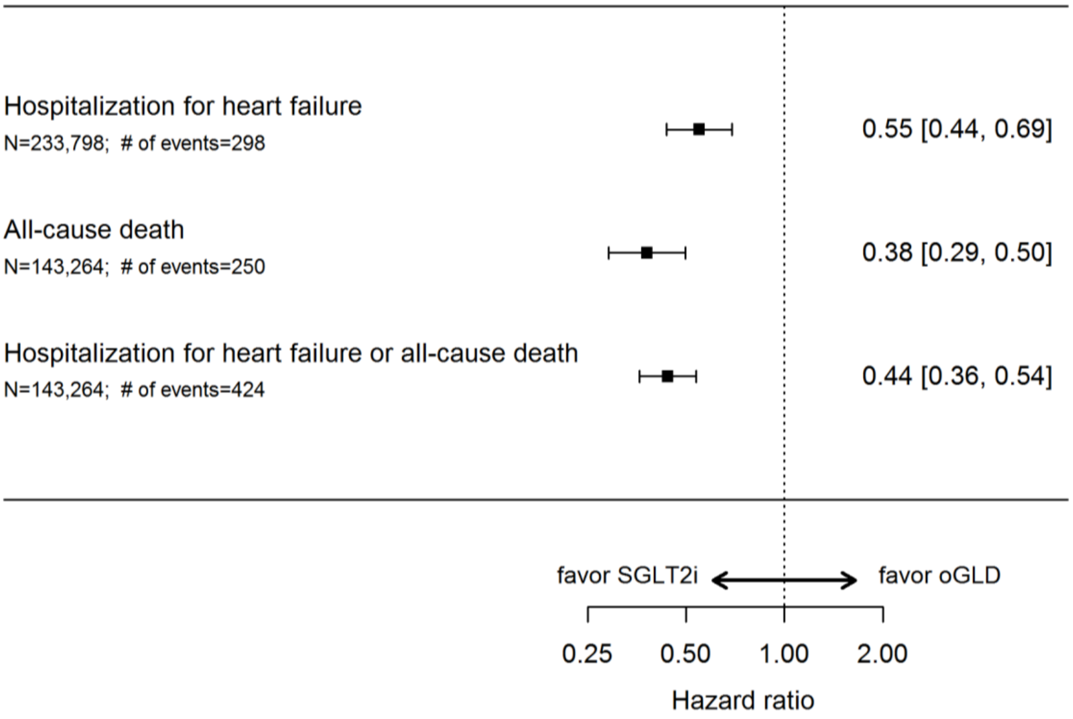

B. European countries combined

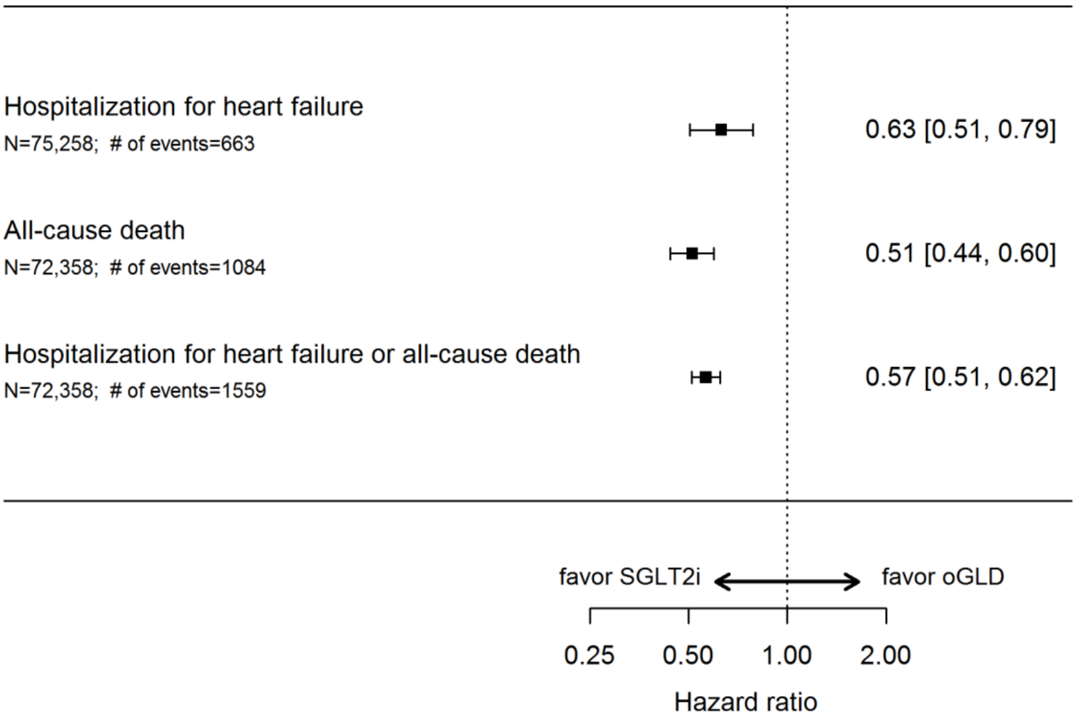

P-value for SGLT-2i versus oGLD comparison <0.001 for all endpoints; oGLD=other glucose-lowering drug;

SGLT-2i=sodium-glucose cotransporter-2; US=United States

References for the supplementary appendices

1. Hansen LG, Chang S. Health research data for the real world: The marketscan® databases. *White Paper*. *Truven Health Analytics, Ann Arbor, USA*, July 2011. http://truvenhealth.com/portals/0/assets/PH_11238_0612_TEMP_MarketScan_WP_FINAL.pdf. Last Accessed: May 12, 2017.
2. Truven Health Analytics. Comparative Effectiveness Research. *Truven Health Analytics, Ann Arbor, USA*, 2012. https://truvenhealth.com/portals/0/assets/ACRS_11228_0912_CompEffectResearch_SS_Web.pdf. Last Accessed: May 12, 2017.
3. Herrett E, Gallagher AM, Bhaskaran K, Forbes H, Mathur R, van Staa T, Smeeth L. Data resource profile: Clinical practice research datalink (cprd). *Int J Epidemiol*. 2015;44:827-836. doi:10.1093/ije/dyv098
4. Herrett E, Thomas SL, Schoonen WM, Smeeth L, Hall AJ. Validation and validity of diagnoses in the general practice research database: A systematic review. *Br J Clin Pharmacol*. 2010;69:4-14. doi:10.1111/j.1365-2125.2009.03537.x
5. Williams T, van Staa T, Puri S, Eaton S. Recent advances in the utility and use of the general practice research database as an example of a uk primary care data resource. *Ther Adv Drug Saf*. 2012;3:89-99. doi:10.1177/2042098611435911
6. Carbonari DM, Saine ME, Newcomb CW, Blak B, Roy JA, Haynes K, Wood J, Gallagher AM, Bhullar H, Cardillo S, Hennessy S, Strom BL, Lo Re V, 3rd. Use of demographic and pharmacy data to identify patients included within both the clinical practice research datalink (cprd) and the health improvement network (thin). *Pharmacoepidemiol Drug Saf*. 2015;24:999-1003. doi:10.1002/pds.3844
7. IMS Health. Statistics. *IMS Health, UK,* 2015. http://www.epic-uk.org/our-data/statistics.shtml. Last Accessed: May 12, 2017.
8. Blak BT, Thompson M, Dattani H, Bourke A. Generalisability of the health improvement network (thin) database: Demographics, chronic disease prevalence and mortality rates. *Inform Prim Care*. 2011;19:251-255.
9. Bourke A, Dattani H, Robinson M. Feasibility study and methodology to create a quality-evaluated database of primary care data. *Inform Prim Care*. 2004;12:171-177.
10. Lewis JD, Schinnar R, Bilker WB, Wang X, Strom BL. Validation studies of the health improvement network (thin) database for pharmacoepidemiology research. *Pharmacoepidemiol Drug Saf*. 2007;16:393-401. doi:10.1002/pds.1335
11. Ruigomez A, Martin-Merino E, Rodriguez LA. Validation of ischemic cerebrovascular diagnoses in the health improvement network (thin). *Pharmacoepidemiol Drug Saf*. 2010;19:579-585. doi:10.1002/pds.1919
12. Cea Soriano L, Soriano-Gabarro M, Garcia Rodriguez LA. Validity and completeness of colorectal cancer diagnoses in a primary care database in the united kingdom. *Pharmacoepidemiol Drug Saf*. 2016;25:385-391. doi:10.1002/pds.3877
13. Martin-Merino E, Ruigomez A, Johansson S, Garcia-Rodriguez LA. Hospitalised ischaemic cerebrovascular accident and risk factors in a primary care database. *Pharmacoepidemiol Drug Saf*. 2011;20:1050-1056. doi:10.1002/pds.2201
14. Vassilev ZP, Ruigomez A, Soriano-Gabarro M, Garcia Rodriguez LA. Diabetes, cardiovascular morbidity, and risk of age-related macular degeneration in a primary care population. *Invest Ophthalmol Visual Sci*. 2015;56:1585-1592. doi:10.1167/iovs.14-16271
15. Rodriguez LA, Ruigomez A, Wallander MA, Johansson S. Acid-suppressive drugs and community-acquired pneumonia. *Epidemiology*. 2009;20:800-806. doi:10.1097/EDE.0b013e3181b5f27d

16. Martin-Merino E, Ruigomez A, Johansson S, Wallander MA, Garcia-Rodriguez LA. Study of a cohort of patients newly diagnosed with depression in general practice: Prevalence, incidence, comorbidity, and treatment patterns. *Prim Care Companion J Clin Psychiatry*. 2010;12:PCC 08m00764. doi:10.4088/PCC.08m00764blu
17. Martin-Merino E, Ruigomez A, Wallander MA, Johansson S, Garcia-Rodriguez LA. Prevalence, incidence, morbidity and treatment patterns in a cohort of patients diagnosed with anxiety in uk primary care. *Fam Pract*. 2010;27:9-16. doi:10.1093/fampra/cmp071
18. Requena G, Abbing-Karahagopian V, Huerta C, De Bruin ML, Alvarez Y, Miret M, Hesse U, Gardarsdottir H, Souverein PC, Slattery J, Schneider C, Rottenkolber M, Schmiedl S, Gil M, De Groot MC, Bate A, Ruigomez A, Garcia Rodriguez LA, Johansson S, de Vries F, Montero D, Schlienger R, Reynolds R, Klungel OH, de Abajo FJ. Incidence rates and trends of hip/femur fractures in five european countries: Comparison using e-healthcare records databases. *Calcif Tissue Int*. 2014;94:580-589. doi:10.1007/s00223-014-9850-y
19. Garcia Rodriguez LA, Johansson S, Nagy P, Cea Soriano L. Use of proton pump inhibitors and the risk of coronary events in new users of low-dose acetylsalicylic acid in uk primary care. *Thromb Haemost*. 2014;111:131-139. doi:10.1160/TH13-07-0542
20. Diabetes-Patienten-Verlaufsdokumentation (DPV). Ulm University-DPV. Ed. Esther Bollow. *Ulm University, Ulm, Germany*. http://buster.zibmt.uni-ulm.de/dpv/index.php/en/. Last Accessed: May 12, 2017.
21. Heidemann C, Du Y, Paprott R, Haftenberger M, Rathmann W, Scheidt-Nave C. Temporal changes in the prevalence of diagnosed diabetes, undiagnosed diabetes and prediabetes: Findings from the german health interview and examination surveys in 1997-1999 and 2008-2011. *Diabet Med*. 2016;33:1406-1414. doi:10.1111/dme.13008
22. Norhammar A, Bodegard J, Nystrom T, Thuresson M, Eriksson JW, Nathanson D. Incidence, prevalence and mortality of type 2 diabetes requiring glucose-lowering treatment, and associated risks of cardiovascular complications: A nationwide study in sweden, 2006-2013. *Diabetologia*. 2016;59:1692-1701. doi:10.1007/s00125-016-3971-y
23. Norwegian Institute of Public Health. Norwegian Cause of Death Registry (DAPHNE database). *Norwegian Institute of Public Health, Oslo, Norway*. https://www.fhi.no/en/hn/health-registries/cause-of-death-registry/. Last Accessed: May 12, 2017.
24. Statistics Denmark. (DAFFODIL database). *Statistics Denmark, Copenhagen, Denmark*. http://www.dst.dk/en. Last Accessed: May 12, 2017.
25. The R Foundation. The R Project for Statistical Computing. https://www.R-project.org/. Last Accessed: May 12, 2017.
26. Viechtbauer W. Conducting meta-analyses in r with the metafor package. *J Stat Soft*. 2010;36:1-48.
